# NRF2 negatively regulates primary ciliogenesis and hedgehog signaling

**DOI:** 10.1371/journal.pbio.3000620

**Published:** 2020-02-13

**Authors:** Pengfei Liu, Matthew Dodson, Deyu Fang, Eli Chapman, Donna D. Zhang

**Affiliations:** 1 Department of Pharmacology and Toxicology, College of Pharmacy, University of Arizona, Tucson, Arizona, United States of America; 2 Department of Pathology, Northwestern University Feinberg School of Medicine, Chicago, Illinois, United States of America; 3 The University of Arizona Cancer Center, University of Arizona, Tucson, Arizona, United States of America; Rheinische Friedrich-Wilhelms-Universitat Bonn, GERMANY

## Abstract

Primary cilia are lost during cancer development, but the mechanism regulating cilia degeneration is not determined. While transcription factor nuclear factor-erythroid 2-like 2 (NRF2) protects cells from oxidative, proteotoxic, and metabolic stress in normal cells, hyperactivation of NRF2 is oncogenic, although the detailed molecular mechanisms by which uncontrolled NRF2 activation promotes cancer progression remain unclear. Here, we report that NRF2 suppresses hedgehog (Hh) signaling through Patched 1 (PTCH1) and primary ciliogenesis via p62/sequestosome 1 (SQSTM1). PTCH1, a negative regulator of Hh signaling, is an NRF2 target gene, and as such, hyperactivation of NRF2 impairs Hh signaling. NRF2 also suppresses primary cilia formation through p62-dependent inclusion body formation and blockage of Bardet–Biedl syndrome 4 (BBS4) entrance into cilia. Simultaneous ablation of PTCH1 and p62 completely abolishes NRF2-mediated inhibition of both primary ciliogenesis and Hh signaling. Our findings reveal a previously unidentified role of NRF2 in controlling a cellular organelle, the primary cilium, and its associated Hh signaling pathway and also uncover a mechanism by which NRF2 hyperactivation promotes tumor progression via primary cilia degeneration and aberrant Hh signaling. A better understanding of the crosstalk between NRF2 and primary cilia/Hh signaling could not only open new avenues for cancer therapeutic discovery but could also have significant implications regarding pathologies other than cancer, including developmental disorders, in which improper primary ciliogenesis and Hh signaling play a major role.

## Abstract 2

This study reveals a novel role for the transcription factor NRF2 in controlling the primary cilium and its associated Hedgehog signaling pathway and also uncovers a mechanism by which NRF2 hyperactivation promotes tumor progression.

## Introduction

Nuclear factor-erythroid 2-like 2 (NRF2) is a transcription factor that mediates cellular redox, metabolic and protein homeostasis [1,2]. Under physiological conditions, NRF2 is negatively regulated by Kelch-like ECH-associated protein 1 (KEAP1), a substrate adaptor protein of the Cullin3 (Cul3)-Ring-Box 1 (Rbx1) E3-ligase complex that targets NRF2 for ubiquitylation and degradation by the 26S proteasome [3]. KEAP1 functions as a molecular sensor through its cysteines, especially C151, controlling activation of the NRF2 pathway [4]. Upon activation, KEAP1-mediated ubiquitylation of NRF2 is blocked, allowing newly synthesized NRF2 to translocate to the nucleus, dimerize with small MAF Transcription Factor (sMAF) proteins, and activate transcription of antioxidant response element (ARE)-containing genes [3,4]. NRF2 target genes mediate myriad cellular functions, including endogenous antioxidant systems, xenobiotic/drug metabolism, iron metabolism, carbohydrate and lipid metabolism, DNA repair, transcription, apoptosis, and proteostasis [5]. Thus, NRF2 and its target genes are essential for maintaining proper cell and organelle homeostasis.

The importance of controlled regulation of NRF2 is exemplified by its dual role in cancer [6,7]. In normal cells, activation of the NRF2 pathway by synthetic or naturally occurring compounds is able to protect against toxicant or carcinogen exposure, thus providing a promising strategy for cancer prevention [7,8]. However, recent evidence has revealed an oncogenic function of NRF2. Gain-of-function mutations in NRF2 and loss-of-function mutations in KEAP1 are found in certain cancers, resulting in high constitutive levels of NRF2, an example of how cancer cells hijack the NRF2 protective response [9–12]. In lung cancer, *KEAP1* is as frequently mutated (>30%) as the tumor suppressor gene tumor protein 53 (*TP53*) [9–11]. Furthermore, mounting evidence has shown that high expression of NRF2 promotes cancer progression and resistance to treatment [13–15]. For example, increased NRF2 has been shown to suppress reactive oxygen species (ROS) formation, enhancing tumorigenesis in Ki-ras2 Kirsten rat sarcoma viral oncogene homolog (K-Ras)–, v-raf murine sarcoma viral oncogene homolog B1 (B-Raf)–, and MYC Proto-Oncogene (Myc)-driven cancers [16]. NRF2 is also critical in maintaining protein translation in pancreatic cancer cells, enhancing serine/glycine biosynthesis to increase glutathione and nucleotide production in non-small–cell lung carcinomas, and facilitating glutathione metabolism via a p21-dependent mechanism in squamous cell carcinomas [17–19]. More recently, we reported that activation of NRF2 accelerates metastasis of existing tumors in mice [20]. Clinically, high expression of NRF2 in patient tumors is strongly correlated with a poor prognosis [21].

Primary cilia are solitary microtubule-based structures that emanate from the surface of most vertebrate cell types. Primary cilia act as cellular “antennae” receiving diverse signals from the extracellular environment and relaying the signal to an intracellular signaling network [22]. It is known that primary cilia act as a brake for cell proliferation, presumably because they require the same structural components as chromosome segregation [23,24]. Therefore, primary cilia are viewed as a tumor suppressor organelle, and the development of cancer is often accompanied by the loss of primary cilia [23,24]. Restoration of primary cilia to prevent proliferation of cancer cells is regarded as a novel and promising approach for cancer therapy [25]. Primary cilia are also important coordinators of a number of relevant physiological and developmental signaling pathways, including the hedgehog (Hh) signaling pathway [26,27]. Hh signaling regulates cell proliferation, cell differentiation, and tissue patterning in the mammalian embryo. An important mediator of Hh signaling is the receptor protein Patched 1 (PTCH1). PTCH1 mediates Hh signaling through smoothened (SMO), which controls a cascade that leads to dissociation of the suppressor of fused homolog-zinc finger protein-GLI (SUFU-GLI) complex and nuclear translocation of GLI to activate transcription of Hh target genes [28–30].

Importantly, an emerging role for the dysregulation of the Hh signaling pathway in cancer progression has also been reported [31,32]. Oncogenic activation of the Hh pathway—for example, as a result of mutations in *SMO* and *SUFU*—is required for the progression of some cancers [29]. Therefore, inhibitors of the Hh pathway hold therapeutic value for cancer treatment, and the development of novel Hh pathway inhibitors has received much attention in recent years [33–35].

Here, we demonstrate that NRF2 is a critical regulator of primary ciliogenesis and the Hh signaling pathway, providing a mechanistic link between NRF2 hyperactivation and the promotion of cancer. Knockdown (KD) of NRF2 enhanced, whereas pharmacological activation or overexpression of NRF2 suppressed, primary cilia formation. PTCH1, a negative regulator of Hh signaling, was demonstrated to have a functional ARE, and increasing NRF2 prevented SMO translocation and suppressed Hh signaling in a PTCH1-dependent manner. Furthermore, NRF2 suppressed primary ciliogenesis by enhancing the expression of p62/sequestosome 1 (SQSTM1), resulting in the sequestration and mislocalization of Bardet–Biedl syndrome 4 (BBS4), a positive regulator of cilia formation. Our data not only reveal a previously unidentified role of NRF2 in controlling key cellular processes (primary ciliogenesis and Hh signaling) but also uncover a mechanism by which NRF2 hyperactivation promotes tumor progression via primary cilia degeneration and aberrant Hh signaling.

## Results

### NRF2 deletion enhances primary ciliogenesis and Hh signaling

**Fig 1:**
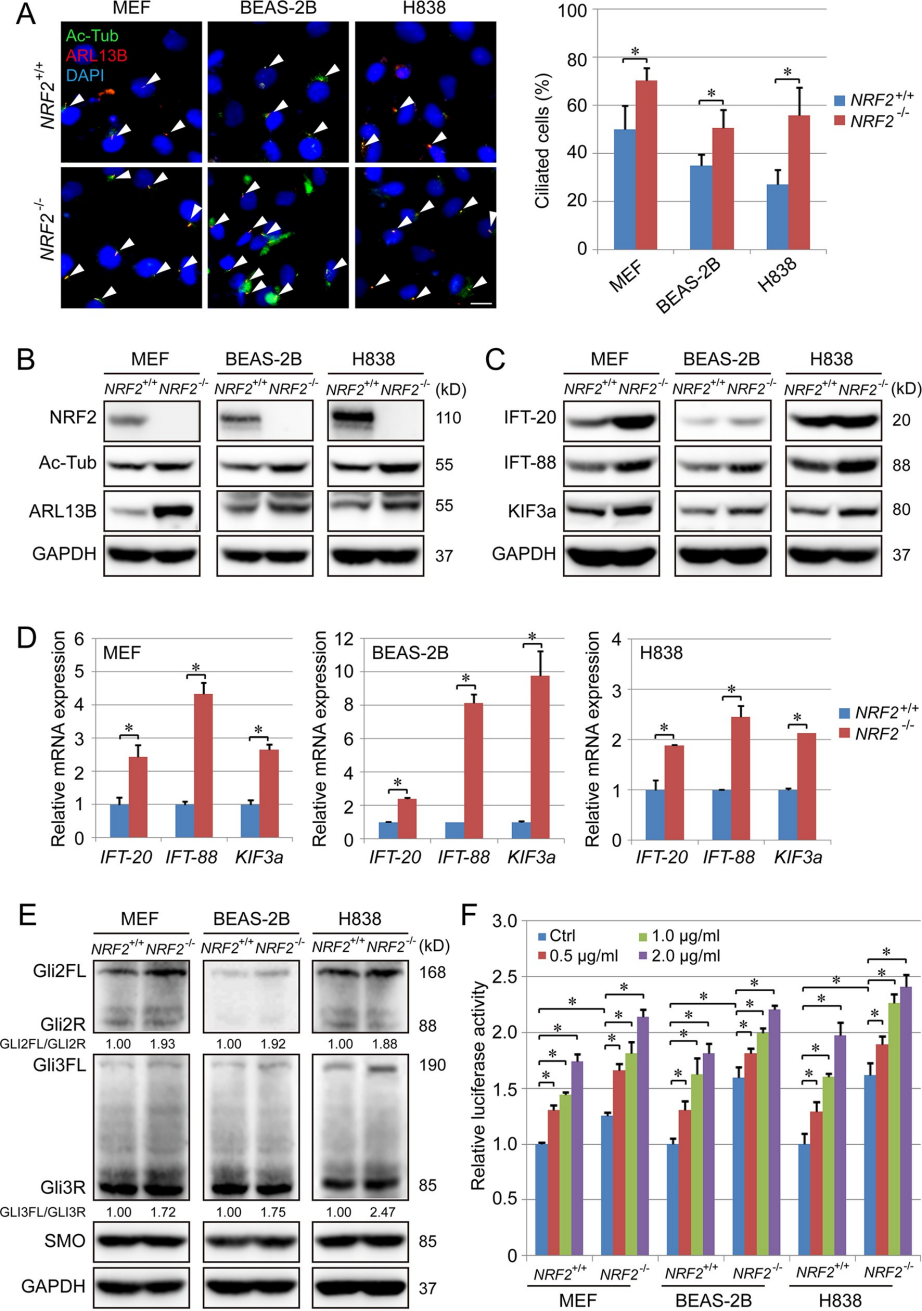
NRF2 deletion enhances ciliogenesis and Hh signaling. (A) IF for Ac-Tub (green) and ARL13B (red) in *NRF2*^+/+^ and *NRF2*^−/−^ MEFs, BEAS-2B, and H838 cell lines. Ciliated cells (%) represent the percentage of Ac-Tub/ARL13B-positive cells normalized to the total number of DAPI-positive cells in 6 random fields. (Scale bar = 10 μm.) (B) Immunoblot analysis of NRF2, Ac-Tub, and ARL13 protein levels in the indicated *NRF2*^+/+^ and *NRF2*^−/−^ cell lines. (C) Immunoblot analysis of IFT-20, IFT-88 and KIF3a protein levels in the indicated *NRF2*^+/+^ and *NRF2*^−/−^ cell lines. (D) qRT-PCR analysis of *IFT-20*, *IFT-88* and *KIF3a* in the indicated *NRF2*^+/+^ and *NRF2*^−/−^ cell lines. (E) Immunoblot analysis of GLI2, GLI3, and SMO expression in the indicated *NRF2*^+/+^ and *NRF2*^−/−^ cell lines. (F) GLI luciferase assay in *NRF2*^+/+^ and *NRF2*^−/−^ cell lines treated with 0, 0.5, 1, or 2 μg/ml Shh for 24 h. Relative quantification of immunoblot results is shown in S1A–S1C Fig. Results are expressed as mean ± SD. A *t* test was used to compare the various groups, and *p* < 0.05 was considered statistically significant. *p < 0.05 compared between the two groups. Ac-Tub, acetylated tubulin; ARL13B, ADP-ribosylation factor-like protein 13B; GAPDH, glyceradehyde-3-phosphate dehydrogenase; Hh, hedgehog; IF, immunofluorescence; *IFT*, intraflagellar transport; *KIF3a*, Kinesin Family Member 3A; MEF, mouse embryonic fibroblast; NRF2, nuclear factor-erythroid 2-like 2; qRT-PCR, Real-Time Quantitative Reverse Transcription PCR; Shh, Sonic hedgehog; SMO, smoothened.

To evaluate the potential role of NRF2 in primary cilia formation, primary cilia were examined in mouse embryonic fibroblasts (MEFs) isolated from Nrf2 wild-type (WT) (*Nrf2*^+/+^) and Nrf2 knockout (*Nrf2*^−/−^) mice. Immunofluorescence (IF) staining for acetylated tubulin (Ac-Tub) and ADP-ribosylation factor-like protein 13B (ARL13B) (two markers for primary cilia) clearly showed more ciliated cells in the *Nrf2*^−/−^ MEF cells compared to the *Nrf2*^+/+^ MEF cells (Fig 1A), as well as higher protein levels of Ac-Tub and ARL13B (Fig 1B, S1A Fig). Consistent with the MEF data, the percentage of ciliated cells and the level of Ac-Tub or ARL13B were significantly higher in *NRF2*^−/−^ BEAS-2B and *NRF2*^−/−^ H838 cell lines compared to their respective *NRF2*^+/+^ controls (Fig 1A and 1B, S1A Fig). The expression level of several genes that play a crucial role in intraflagellar transport (intraflagellar transport-20 [*IFT20*], intraflagellar transport-88 [*IFT88*], and Kinesin Family Member 3A [*KIF3a*]) were also enhanced by *NRF2* knockout (Fig 1C and 1D, S1B Fig), indicating that primary cilia are negatively regulated by NRF2. To determine if the lack of cilia was a result of decreased ciliogenesis or improper break down of primary cilia, ciliary disassembly was evaluated by measuring colocalization of the primary components (NudE Neurodevelopment Protein 1 [NDE1], oral–facial–digital syndrome 1 [OFD1], and Aurora A) of the cilium disassembly complex (CDC) with the primary cilia itself, as reported previously [36,37]. As shown in S2 Fig, the percentage of cilia that exhibited colocalization of each CDC component was similar between *NRF2*^+/+^ cells and *NRF2*^−/−^ cells. Furthermore, activation of the complex was also evaluated by measuring colocalization of Ac-Tub with active Aurora A (phospho T288); however, similar to the nonphosphorylated form, there was no significant difference in colocalization between *NRF2*^+/+^ cells and *NRF2*^−/−^ cells. While this suggests that the negative effect of NRF2 on primary cilia formation is most likely through inhibition of ciliogenesis, future studies to further clarify NRF2 regulation of primary cilium assembly/disassembly are still needed.

Because primary cilia are essential for transduction of the Hh signal, the effect of NRF2 on Hh signaling was also examined. Hh signaling is primarily mediated by the transcription factors GLI2 and GLI3, which coexist as N-terminal repressor (R) and full-length activator (FL) forms. Thus, the ratio of FL/R can be used to evaluated the activation of the Hh signal pathway [35,38]. Interestingly, the GLIFL/R ratio of both GLI2 and GLI3 was significantly increased in *NRF2*^−/−^ compared to *NRF2*^+/+^ cell lines, while the expression of *SMO* was unchanged (Fig 1E, S1C Fig). To further evaluate the effect of NRF2 on Hh signaling, GLI transcriptional activity was also assessed by GLI luciferase assay in the presence or absence of Sonic hedgehog (Shh), a known Hh pathway activator. As expected, the GLI luciferase activity was increased by Shh in a dose-dependent manner in both *NRF2*^+/+^ and *NRF2*^−/−^ cell lines; however, the basal level in *NRF2*^−/−^ cells was higher than *NRF2*^+/+^ cells (Fig 1F), and the response to Shh was slightly diminished in *NRF2*^−/−^ BEAS-2B and H838 cells compared with the WT cells (S1D Fig).

### NRF2-mediated suppression of primary ciliogenesis and Hh signaling is accompanied by enhanced PTCH1 expression and impaired ciliary entrance of SMO

**Fig 2:**
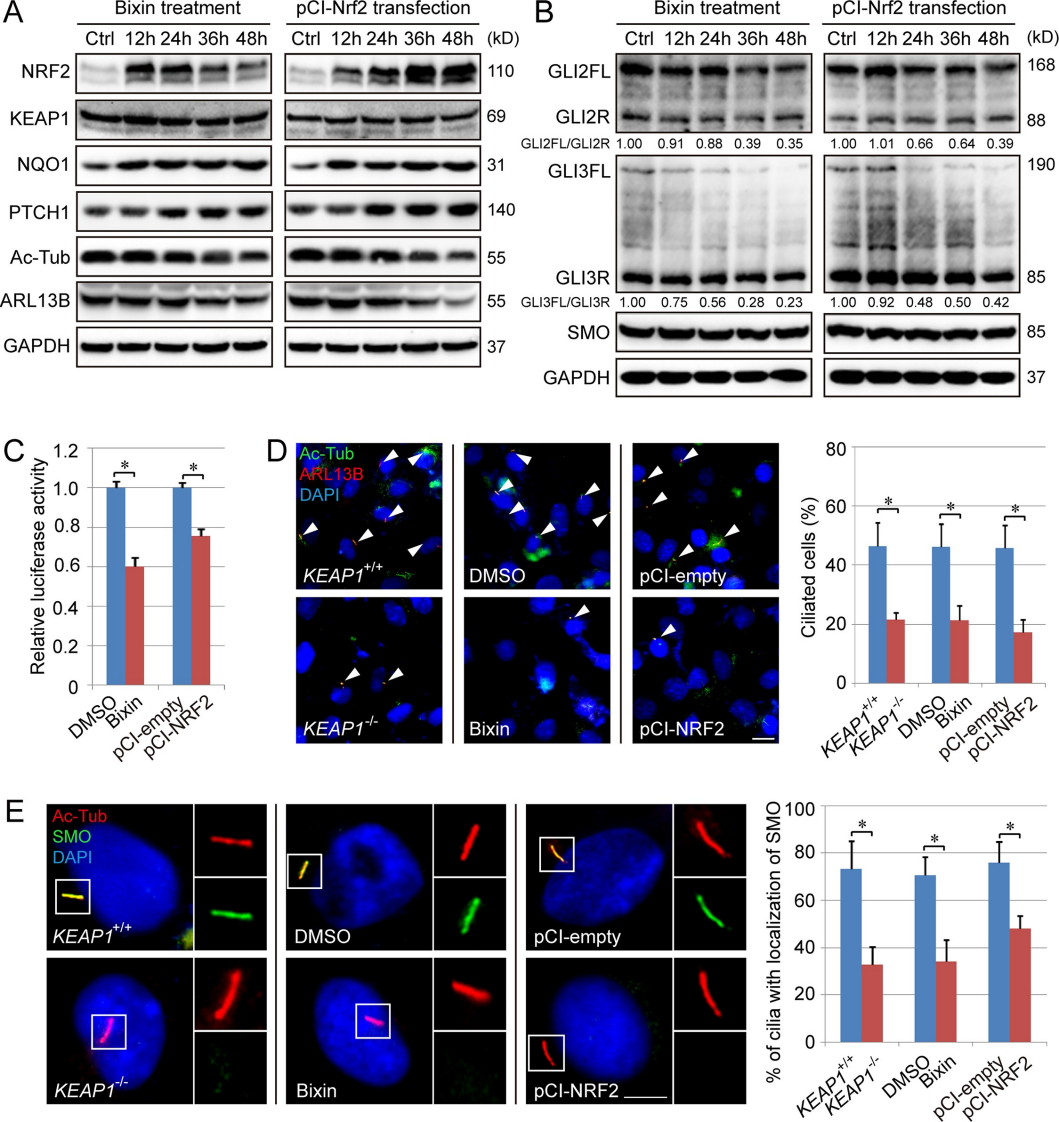
NRF2 negatively regulates Hh signaling, ciliogenesis, and ciliary translocation of SMO. (A–B) H1299 cells were treated with bixin (40 μM) or transfected with pCI-NRF2 vector for 0, 12, 24, 36, or 48 h and subjected to immunoblot analysis of key Hh and ciliary proteins. (C) GLI luciferase assay in H1299 cells treated with bixin or vector transfection for 48 h. (D–E) *KEAP1*^−/−^, bixin-treated (40 μM for 48 h), and pCI-NRF2–transfected (for 48 h) H1299 cells were subjected to IF analysis of (D) % ciliated cells or (E) colocalization of Ac-Tub (green) and SMO (red) (D: scale bar = 10 μm; E: scale bar = 5 μm, *n* = 150). Relative quantification of immunoblot results is shown in S3A and S3B Fig. Results are expressed as mean ± SD. A *t* test was used to compare the various groups, and *p* < 0.05 was considered statistically significant. *p < 0.05 compared between the two groups. Ac-Tub, acetylated tubulin; ARL13B, ADP-ribosylation factor-like protein 13B; GAPDH, glyceraldehyde-3-phosphate dehydrogenase; Hh, hedgehog; IF, immunofluorescence; KEAP1, Kelch-like ECH-associated protein 1; NQO1, NAD(P)H Quinone Dehydrogenase 1; NRF2, nuclear factor-erythroid 2-like 2; PTCH1, Patched 1; SMO, smoothened.

NRF2 up-regulation, either by bixin treatment (C151-dependent activator) or ectopic expression of NRF2, increased NAD(P)H Quinone Dehydrogenase 1 (NQO1) (a well-defined NRF2-target gene) and PTCH1 levels but had no effect on KEAP1; in contrast, the level of Ac-Tub and ARL13B gradually decreased over time (Fig 2A, S3A Fig). The ratio between GLIFL and GLIR (GLI2FL/GLI2R and GLI3FL/GLI3R), as well as GLI luciferase activity, was also reduced with bixin treatment and NRF2 overexpression, whereas there was no change in SMO protein levels (Fig 2B and 2C, S3B Fig). In addition, the percentage of ciliated cells was also significantly reduced upon NRF2 up-regulation, as seen in cells with KEAP1 knockout, bixin treatment, or NRF2 overexpression (Fig 2D). Furthermore, NRF2 up-regulation, either pharmacologically or genetically, inhibited the ciliary entrance of SMO (Fig 2E). In addition, the effect of bixin on inhibition of primary ciliogenesis and Hh signaling is NRF2-dependent, as the inhibitory effects of bixin were lost in *NRF2*^−/−^ cells as compared with the WT control (S4 Fig). To exclude the possibility that the negative effect of NRF2 on primary cilia is due to an indirect effect of NRF2 on cell proliferation, cell cycle status was further analyzed via propidium iodide (PI) staining and fluorescence-activated cell sorting (FACS). The percentage of cells in the different phases of the cell cycle were similar regardless of NRF2 status, with over 70% of the cells being in G0/G1 phase (S5 Fig).

### PTCH1 is an NRF2 target gene

**Fig 3:**
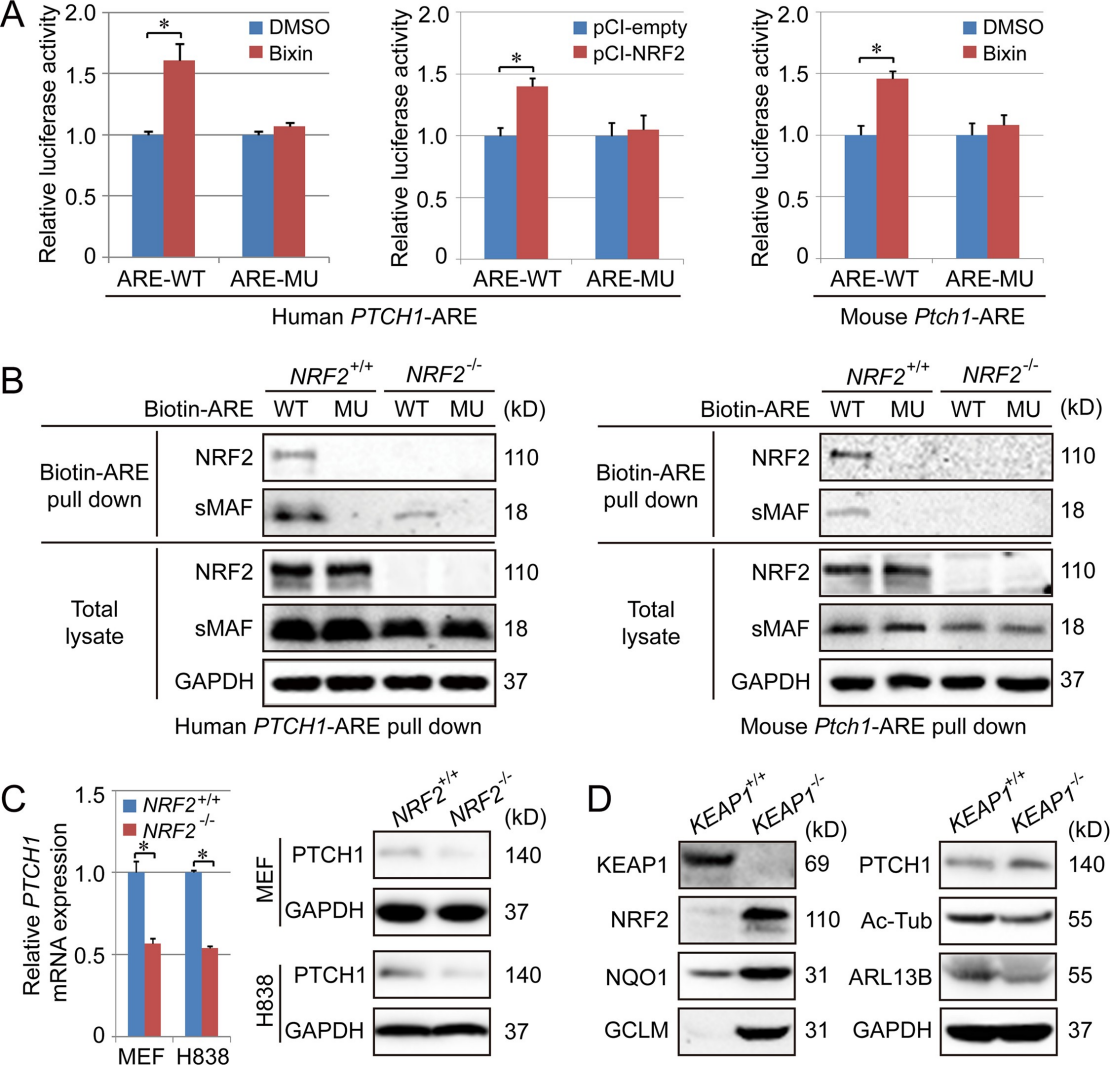
PTCH1 is an NRF2-target gene. (A) H1299 and MEF cells were transfected with pGL4.22 vector containing the promoter of human *PTCH1* or the promoter of mouse *Ptch1* and further treated with bixin (40 μM) for 16 h, followed by a dual luciferase assay. (B) Biotinylated *PTCH1*-ARE-WT and *PTCH1*-ARE-MU were incubated with whole-cell lysates from H838 (for human *PTCH1*-ARE) or MEF cells (for mouse *Ptch1*-ARE), and ARE-bound proteins were pulled down by streptavidin beads and detected by immunoblot analysis with anti-NRF2 and anti-sMAF antibodies. (C) qPCR (left panel) and immunoblot (right panel) analysis of PTCH1 levels in the indicated *NRF2*^+/+^ and *NRF2*^−/−^ cells. Relative quantification of immunoblot is shown in S6D Fig. (D) Immunoblot analysis of NRF2, NQO1, GCLM, Ac-Tub, ARL13B, and PTCH1 in *KEAP1*^+/+^ and *KEAP1*^−/−^ H1299 cell lines. Relative quantification of immunoblot results is shown in S6E Fig. Results are expressed as mean ± SD. A *t* test was used to compare the various groups, and *p* < 0.05 was considered statistically significant. *p < 0.05 compared between the two groups. Ac-Tub, acetylated tubulin; ARE, antioxidant response element; ARL13B, ADP-ribosylation factor-like protein 13B; GAPDH, glyceraldehyde-3-phosphate dehydrogenase; GCLM, Glutamate-Cysteine Ligase Modifier Subunit; KEAP1, Kelch-like ECH-associated protein 1; MEF, mouse embryonic fibroblast; MU, mutation; NQO1, NAD(P)H Quinone Dehydrogenase 1; NRF2, nuclear factor-erythroid 2-like 2; PTCH1, Patched 1; sMAF, small MAF; WT, wild type.

In silico analysis identified a putative ARE sequence in the promoter region of *PTCH1* (Human: ^-1130^-ATGACTCTGCT^-1120^; Mouse: ^-1266^ATGACTCAGAA^-1256^). To verify functionality of these AREs, a reporter gene luciferase construct containing a 41-bp putative ARE-containing sequence from mouse or human *PTCH1* (S6A Fig) was cloned into a luciferase expression vector and transfected into various NRF2 overexpressing cell types (Fig 3A, S6B and S6C Fig). Furthermore, NRF2-sMAF binding to the ARE was confirmed since only the ARE-WT (both the human and mouse ARE) was able to pull down NRF2 and sMAF in *NRF2*^+/+^ cells, but not *NRF2*^−/−^ cells (Fig 3B). Utilizing *NRF2*^+/+^ and *NRF2*^−/−^ H838 cells, *Nrf2*^+/+^ and *Nrf2*^−/−^ MEF cells (Fig 3C, S6D Fig), or *KEAP1*^+/+^ and *KEAP1*^−/−^ H1299 cells (Fig 3D, S6E Fig), NRF2-mediated positive regulation on PTCH1 at both the mRNA and protein level was confirmed. The positive correlation between NRF2 and PTCH1 expression was also confirmed in human lung cancer tissues (S6F Fig).

### PTCH1 deletion rescues the ciliary entrance of SMO but only has a partial effect on NRF2-mediated suppression of primary cilia and Hh signaling

**Fig 4:**
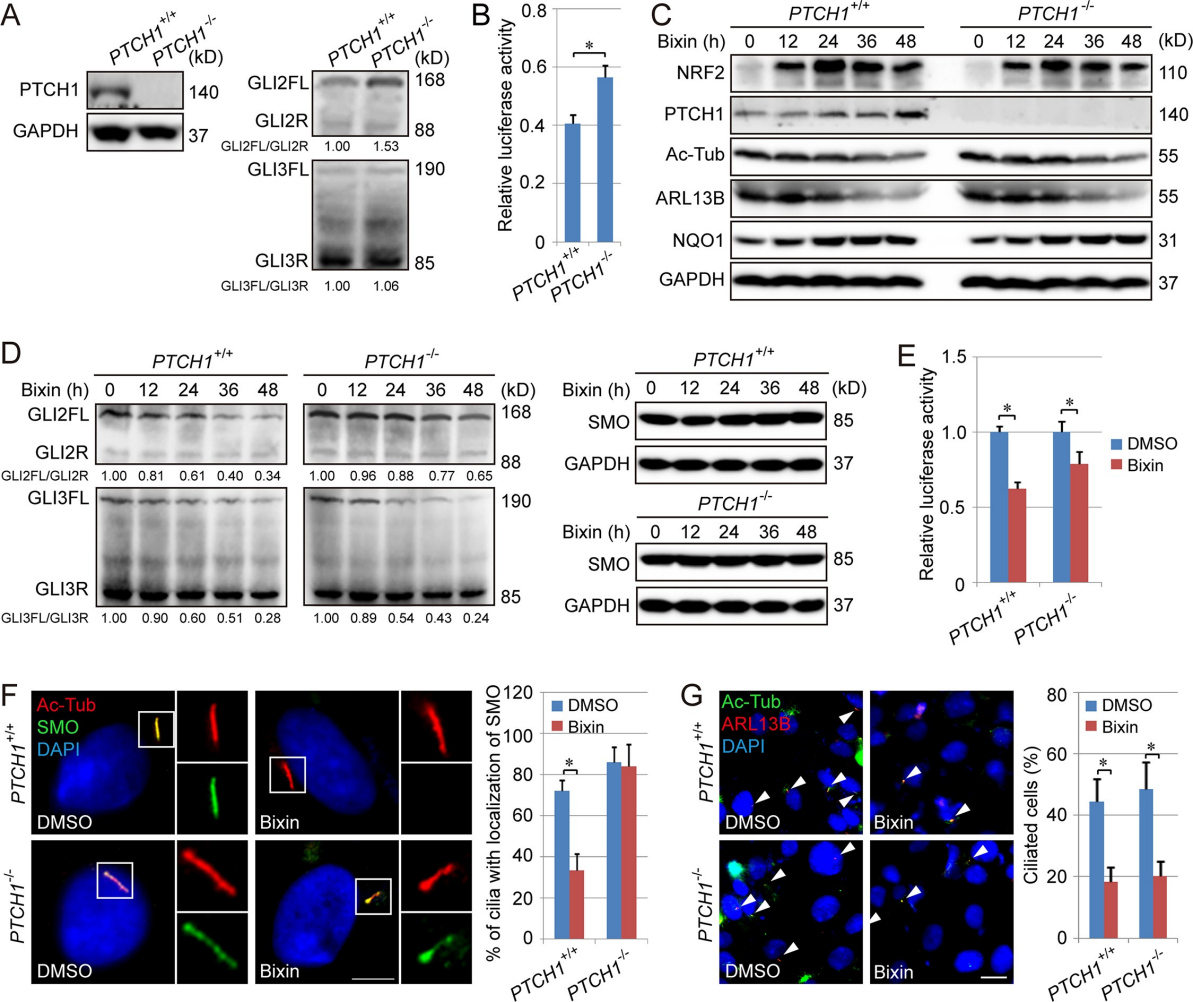
PTCH1 is required for NRF2-mediated inhibition of ciliary translocation of SMO but not the suppression of primary ciliogenesis by NRF2. (A) Immunoblot analysis of PTCH1, GLI2FL/R, and GLI3FL/R protein levels in a *PTCH1*^−/−^ H1299 cell line. Relative quantification of immunoblot results is shown in S7A Fig. (B) GLI luciferase assay in *PTCH1*^+/+^ and *PTCH1*^−/−^ H1299 cells. (C) Immunoblot analysis of PTCH1, Ac-Tub, ARL13B, and NQO1 protein levels following treatment with bixin (40 μM) for 0, 12, 24, 36, or 48 h in *PTCH1*^+/+^ and *PTCH1*^−/−^ H1299 cells. Relative quantification of immunoblot results is shown in S7B Fig. (D) Immunoblot analysis of GLI2FL/R and GLI3FL/R, as well as SMO protein levels, following treatment with bixin (40 μM) for 0, 12, 24, 36, or 48 h in *PTCH1*^+/+^ and *PTCH1*^−/−^ H1299 cells. Relative quantification of immunoblot results is shown in S7C Fig. (E) GLI luciferase assay in the cells treated with bixin for 48 h. (F) IF analysis of Ac-Tub (green) and SMO (red) colocalization in *PTCH1*^+/+^ and *PTCH1*^−/−^ H1299 cells treated with bixin (40 μM) for 48 h. (Scale bar = 5 μm.) (G) % ciliated cells in *PTCH1*^+/+^ and *PTCH1*^−/−^ H1299 cells treated with bixin (40 μM) for 48 h. (Scale bar = 10 μm.) Results are expressed as mean ± SD. A *t* test was used to compare the various groups, and *p* < 0.05 was considered statistically significant. *p < 0.05 compared between the two groups. Ac-Tub, acetylated tubulin; ARL13B, ADP-ribosylation factor-like protein 13B; FL, full-length activator; GAPDH, glyceraldehyde-3-phosphate dehydrogenase; IF, immunofluorescence; NQO1, NAD(P)H Quinone Dehydrogenase 1; NRF2, nuclear factor-erythroid 2-like 2; PTCH1, Patched 1; R, repressor; SMO, smoothened.

To explore whether PTCH1 is critical in NRF2-mediated repression of Hh signaling and primary cilia function, *PTCH1*^+/+^ and *PTCH1*^−/−^ H1299 cells were established. Deletion of *PTCH1* resulted in activation of Hh signaling, as confirmed by an increase in the ratio of GLI2FL/GLI2R and GLI luciferase activity in *PTCH1*^−/−^ cells, although no obvious difference in the ratio of GLI3FL/GLI3R was observed (Fig 4A and 4B, S7A Fig). With bixin treatment, the levels of NRF2 and NQO1 were up-regulated in both *PTCH1*^+/+^ and *PTCH1*^−/−^ cells, while the level of Ac-Tub and ARL13B gradually decreased in both cell lines (Fig 4C, S7B Fig), indicating that deletion of PTCH1 had no significant effects on NRF2-mediated repression of primary ciliogenesis. Interestingly, the ratio of GLI2FL/GLI2R and GLI luciferase activity were reduced with bixin treatment in both *PTCH1*^+/+^ and *PTCH1*^−/−^ cells, but the reduction was diminished in *PTCH1*^−/−^ cells (Fig 4D and 4E, S7C Fig). Furthermore, although *PTCH1* deletion recovered ciliary entrance of SMO in cells treated with bixin (Fig 4F), it had no detectable effects on the reduction of percentage of ciliated cells in response to bixin treatment (Fig 4G). Collectively, these results demonstrate PTCH1 is only partially responsible for NRF2-mediated suppression of Hh signaling.

### NRF2 inhibits primary ciliogenesis and Hh signaling by up-regulating p62 expression, increasing inclusion body formation, and suppressing ciliary entrance of BBS4

**Fig 5:**
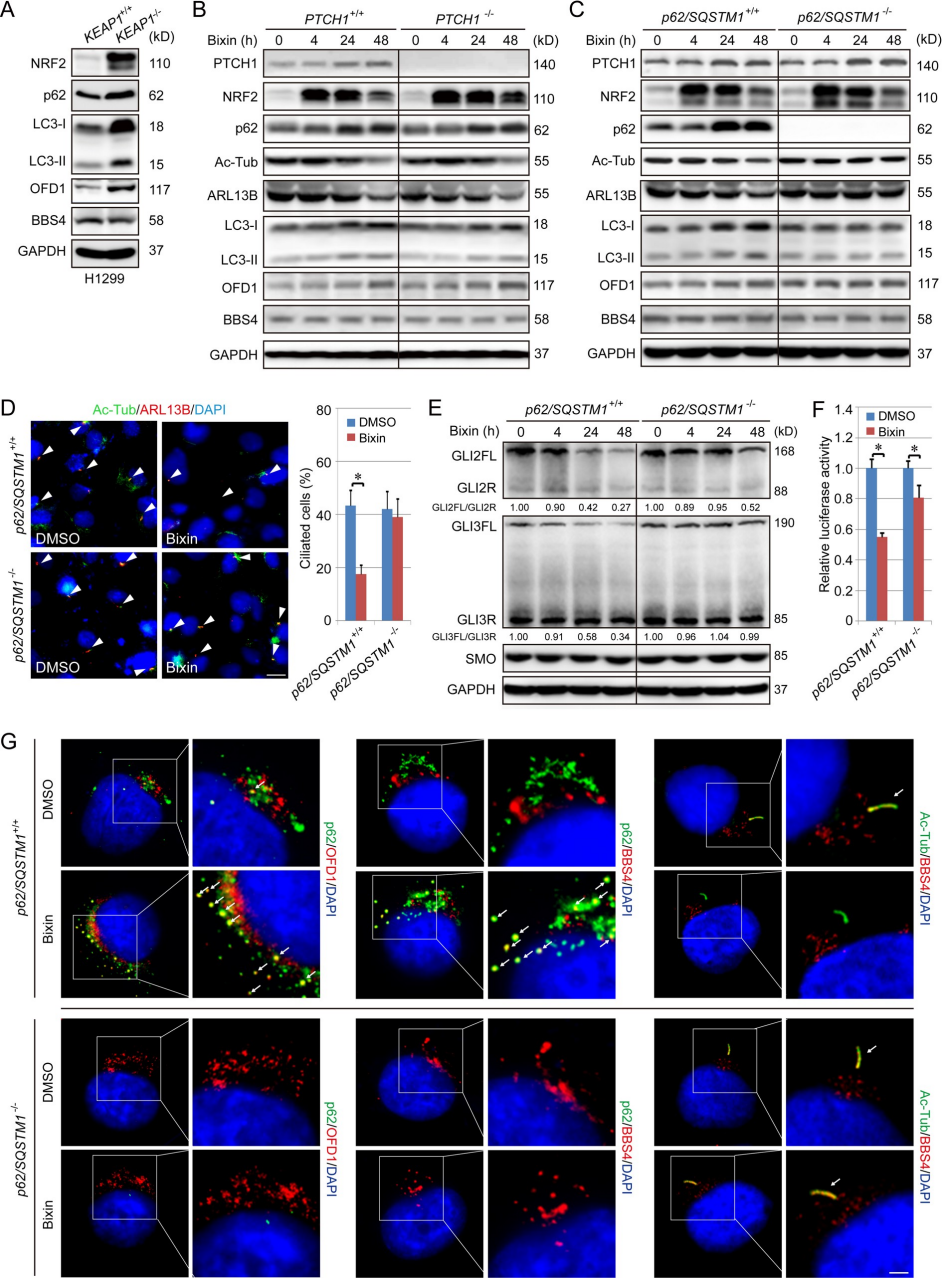
NRF2 inhibits ciliogenesis by increasing p62-dependent inclusion body formation and suppressing the ciliary entrance of BBS4. (A) Immunoblot analysis of p62, LC3-I and II, OFD1, and BBS4 in *KEAP1*^+/+^ and *KEAP1*^−/−^ H1299 cells. Relative quantification of immunoblot results is shown in S8A Fig. (B) Immunoblot analysis of NRF2, p62, Ac-Tub, ARL13B, LC3-I and II, OFD1, and BBS4 in *PTCH1*^+/+^ and *PTCH1*^−/−^ H1299 cells treated with bixin (40 μM) for 0, 4, 24, or 48 h. Relative quantification of immunoblot results is shown in S8B Fig. (C) Immunoblot analysis of NRF2, p62, Ac-Tub, ARL13B, OFD1, and BBS4 in *p62*/*SQSTM1*^+/+^ and *p62*/*SQSTM1*^−/−^ H1299 cells treated with bixin (40 μM) for 0, 4, 24, or 48 h. Relative quantification of immunoblot results is shown in S8C Fig. (D) Percent ciliated cells in *p62*/*SQSTM1*^+/+^ and *p62*/*SQSTM1*^−/−^ H1299 cells treated with bixin (40 μM) for 48 h. (Scale bar = 10 μm.) (E) Immunoblot analysis of GLI2FL/R and GLI3FL/R, as well as SMO protein levels following treatment with bixin (40 μM) for 0, 4, 24, or 48 h in *p62*/*SQSTM1*^+/+^ and *p62*/*SQSTM1*^−/−^ H1299 cells. (F) GLI luciferase assay in *p62*/*SQSTM1*^+/+^ and *p62*/*SQSTM1*^−/−^ cells treated with bixin for 48 h. Results are expressed as mean ± SD. A *t* test was used to compare the various groups, and *p* < 0.05 was considered statistically significant. *p < 0.05 compared between the two groups. (G) IF for colocalization of p62 (green)/OFD1 (red), p62 (green)/BBS4 (red), and Ac-Tub (green)/BBS4 (red), in *p62*/*SQSTM1*^+/+^ and *p62*/*SQSTM1*^−/−^ H1299 cells was performed. Areas of colocalization are indicated by white arrows (scale bar = 3 μm). Ac-Tub, acetylated tubulin; ARL13B, ADP-ribosylation factor-like protein 13B; BBS4, Bardet–Biedl syndrome 4; FL, full-length activator; GAPDH, glyceraldehyde-3-phosphate dehydrogenase; IF, immunofluorescence; KEAP1, Kelch-like ECH-associated protein 1; LC3, microtubule-associated proteins 1A/1B light chain 3B; NRF2, nuclear factor-erythroid 2-like 2; OFD1, oral–facial–digital syndrome 1; PTCH1, Patched 1; R, repressor; SMO, smoothened; SQSTM1, sequestosome 1.

It has been reported that OFD1 accumulation at centriolar satellites due to dysregulation of autophagy results in defective ciliary recruitment of BBS4 and shorter/fewer primary cilia and that OFD1 depletion promotes cilia formation [39]. Therefore, the possible connection between NRF2 and OFD1 or BBS4 was investigated. In *KEAP1*^−/−^ cells, high NRF2 levels resulted in up-regulation of p62/SQSTM1 (Fig 5A), which is consistent with p62 being an NRF2-target gene [40,41]. The protein levels of OFD1, as well as microtubule-associated proteins 1A/1B light chain 3B (LC3)-II, an indicator of autophagosome number, were also increased (Fig 5A, S8A Fig). The bixin-mediated increase in the protein levels of LC3-I, LC3-II, and OFD1 were not affected by *PTCH1* deletion (Fig 5B, S8B Fig) but did depend on p62 and NRF2 (Fig 5C, S8C and S8D Fig). Next, the importance of p62 in NRF2-mediated suppression of primary ciliogenesis and Hh signaling was tested. As shown in Fig 5D, p62 is required for the reduction of ciliated cells upon NRF2 up-regulation by bixin. Furthermore, bixin partially lost its effect on the ratio of GLIFL/GLIR (especially GLI3FL/GLI3R) and GLI luciferase activity in *p62*/*SQSTM1*^−/−^ cells (Fig 5E and 5F, S8E Fig).

Since p62 is a critical protein required for formation of protein aggregates, and increased levels of p62 can sequester its interacting partners (for example, LC3) into protein/ubiquitination (Ub)-containing inclusion bodies, a monomeric red fluorescent protein (mRFP)–green fluorescent protein (GFP)–LC3 reporter construct was utilized to test the requirement of PTCH1 and p62 in mediating inclusion body formation. Bixin treatment resulted in an accumulation of LC3 positive puncta, which are p62-, but not PTCH1-, dependent (S9A and S9B Fig). Next, whether increased OFD1, which has also been shown to interact with LC3, was sequestering BBS4 into the p62-positive inclusion bodies was tested. Indirect IF revealed that bixin increased the colocalization of p62/OFD1 and p62/BBS4, as well as decreased the ciliary entrance of BBS4 in *p62*/*SQSTM1*^+/+^ cells treated with bixin (Fig 5G). However, in *p62*/*SQSTM1*^−/−^ cells, bixin treatment had no effect on OFD1 expression/localization (Fig 5C, S8C Fig) or the ciliary entry of BBS4 (Fig 5G). Taken together, these results indicate that NRF2 inhibits primary ciliogenesis and Hh signaling by increasing the expression of p62, resulting in sequestration of OFD1 and BBS4 into inclusion bodies at centriolar satellites, blockage of cilia entry of BBS4, and thus the subsequent suppression of primary cilium formation.

### Simultaneous ablation of PTCH1 and p62 abolishes NRF2-mediated effects on both primary ciliogenesis and Hh signaling

**Fig 6:**
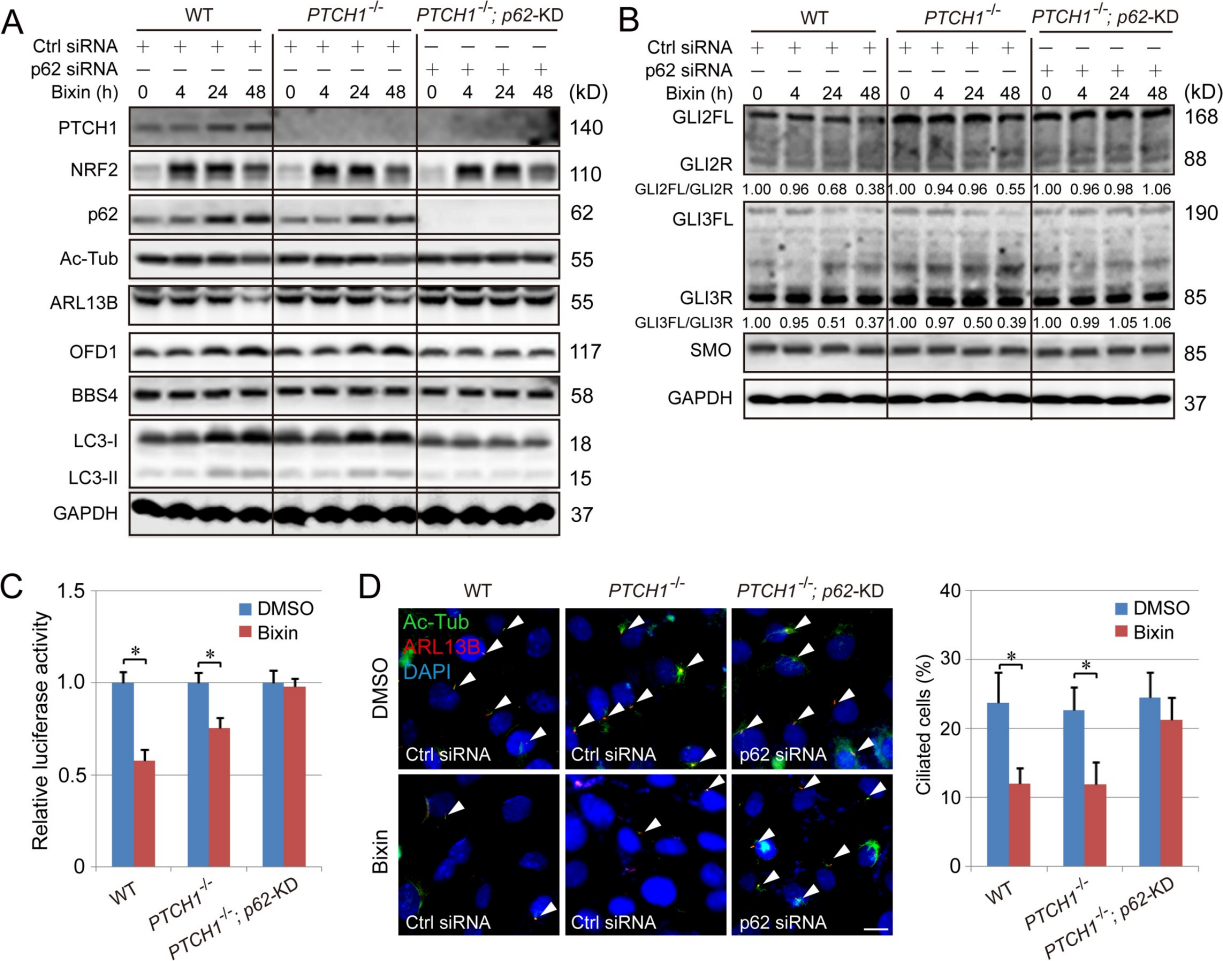
Hh signaling cannot be regulated by NRF2 in *PTCH1*^−/−^;*p62*-knockdown cells. (A) Immunoblot analysis of PTCH1, NRF2, p62, Ac-Tub, ARL13B, LC3-I and II, OFD1, and BBS4 in WT, *PTCH1*^−/−^, and *PTCH1*^−/−^;*p62*-knockdown H1299 cells treated with bixin (40 μM) for 0, 4, 24, or 48 h. Relative quantification of immunoblot results is shown in S10A Fig. (B) Immunoblot analysis of GLI2FL/R and GLI3FL/R, as well as SMO protein levels in WT, *PTCH1*^−/−^ and *PTCH1*^−/−^;*p62*-knockdown H1299 cells treated with bixin (40 μM) for 0, 4, 24, or 48 h. Relative quantification of immunoblot results is shown in S10B Fig. (C) GLI luciferase assay in WT, *PTCH1*^−/−^, and *PTCH1*^−/−^;*p62*-knockdown H1299 cells treated with bixin for 48 h. (D) Percent ciliated cells in WT, *PTCH1*^−/−^, and *PTCH1*^−/−^;*p62*-knockdown H1299 cells treated with bixin (40 μM) for 48 hr. (Scale bar = 10 μm.) Results are expressed as mean ± SD. A *t* test was used to compare the various groups, and *p* < 0.05 was considered statistically significant. *p < 0.05 compared between the two groups. Ac-Tub, acetylated tubulin; ARL13B, ADP-ribosylation factor-like protein 13B; BBS4, Bardet–Biedl syndrome 4; Hh, hedgehog; KD, knockdown; LC3, microtubule-associated proteins 1A/1B light chain 3B; NRF2, nuclear factor-erythroid 2-like 2; OFD1, oral–facial–digital syndrome 1; PTCH1, Patched 1; siRNA, small interfering RNA; SMO, smoothened; WT, wild type.

Next, we tested whether depletion of PTCH1 and p62 is sufficient to block NRF2-mediated negative regulation on both Hh signaling and primary ciliogenesis. CRISPR knockout of p62/SQSTM1 in *PTCH1*^−/−^ cells or knockout of PTCH1 in *p62*/*SQSTM1*^+/+^ cells failed to generate any viable clones. Therefore, p62-small interfering RNA (siRNA) was used to transiently KD p62. The NRF2-mediated increase in the protein levels of LC3-I, LC3-II, and OFD1 was observed in WT and *PTCH1*^−/−^ cells, but not in *PTCH1*^−/−^; *p62*-KD cells (Fig 6A, S10A Fig). Importantly, the reduction in the ratio of GLI2FL/GLI2R and GLI3FL/GLI3R, as well as GLI luciferase activity, observed in WT and *PTCH1*^−/−^ cells in response to bixin was completely abolished in the *PTCH1*^−/−^;*p62*-KD cells (Fig 6B and 6C, S10B Fig). Similarly, NRF2-mediated reduction of ciliated cells was also lost in *PTCH1*^−/−^;*p62*-KD cells (Fig 6D). These results demonstrate that NRF2 negatively regulates Hh signaling and primary ciliogenesis through its target genes *PTCH1* and *p62*.

### NRF2 is required for HPI-4–mediated inhibition of ciliogenesis and Hh signaling

**Fig 7:**
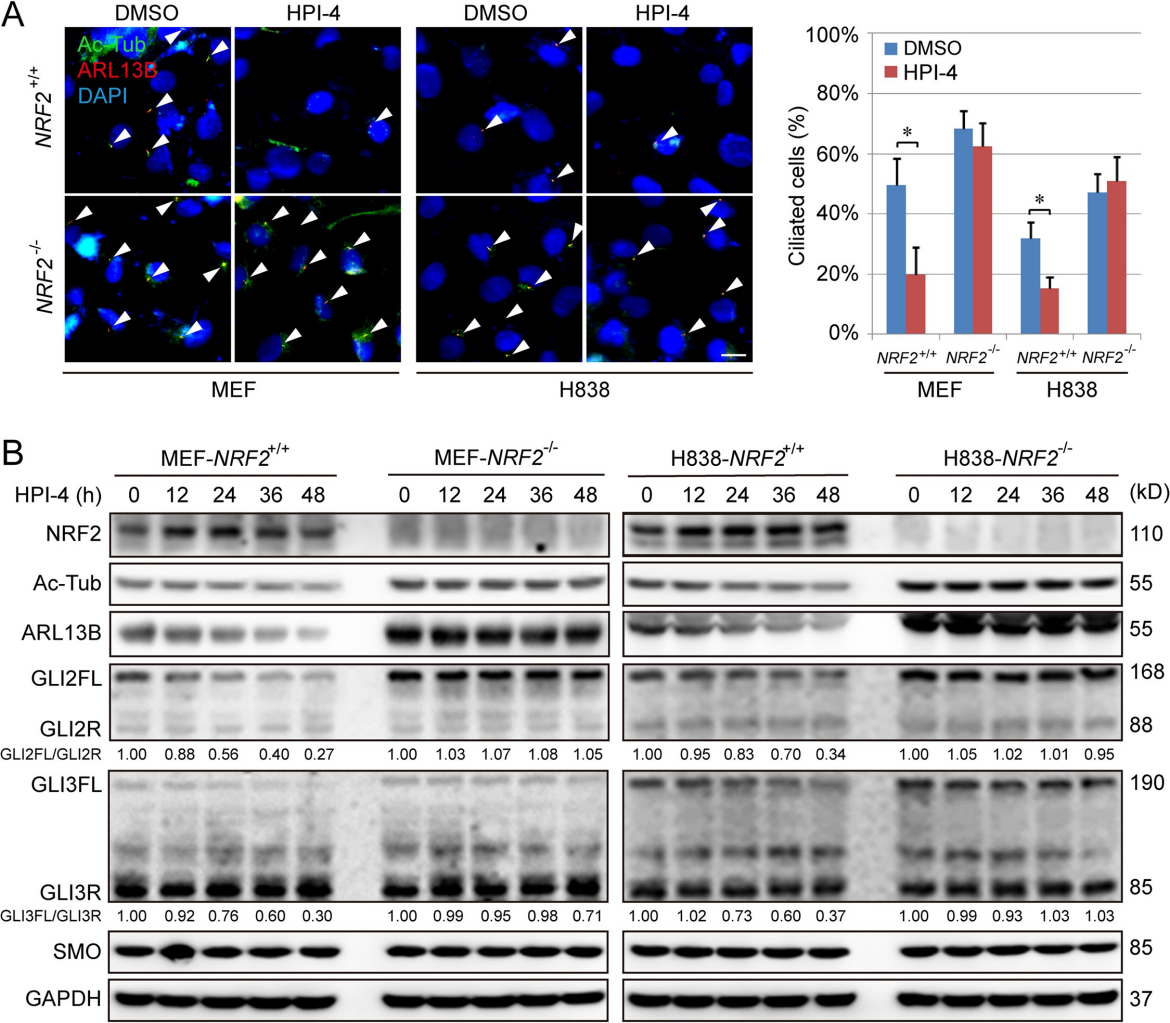
HPI-4 inhibits the formation of primary cilia in an NRF2-dependent manner. (A) IF for Ac-Tub (green) and ARL13B (red) in MEF and H838 cell lines treated with HPI-4 (20 μM) for 48 h. Both *NRF2*^+/+^ and *NRF2*^−/−^ cell lines were analyzed, the percentage of Ac-Tub/ARL13B-positive cells in 6 random fields was summarized, and total cell number was determined by DAPI stain. (Scale bar = 10 μm.) (B) Immunoblot analysis of NRF2, Ac-Tub, ARL13B, and Hh signaling pathway expression in different *NRF2*^+/+^ and *NRF2*^−/−^ cell lines treated with HPI-4. Similar results were obtained in at least 3 independent experiments. Results are expressed as mean ± SD. A *t* test was used to compare the various groups, and *p* < 0.05 was considered statistically significant. *p < 0.05 compared between the two groups. Ac-Tub, acetylated tubulin; ARL13B, ADP-ribosylation factor-like protein 13B; GAPDH, glyceraldehyde-3-phosphate dehydrogenase; Hh, hedgehog; HPI-4, hedgehog pathway inhibitor-4; IF, immunofluorescence; MEF, mouse embryonic fibroblast; NRF2, nuclear factor-erythroid 2-like 2; SMO, smoothened.

Hedgehog pathway inhibitor-4 (HPI-4, ciliobrevin A) is a small molecule that was discovered during a high-throughput screen for Hh pathway antagonists and later reported to disrupt ciliogenesis and inhibit Hh signaling [35,42]. Thus, we next aimed to determine whether HPI-4 effects were NRF2-dependent. HPI-4 induced NRF2 and NQO1 in a KEAP1-dependent manner (S11A Fig). In addition, HPI-4 activation of the NRF2 pathway required KEAP1-C151 (S11B Fig), indicating that HPI-4 is a canonical NRF2 inducer similar to bixin. Moreover, the effect of HPI-4 on inhibiting primary cilia and Hh signaling requires NRF2, as evidenced by the fact that HPI-4 significantly reduced percentage of ciliated cells in both *Nrf2*^+/+^ MEF and *NRF2*^+/+^ H838 cells; however, no significant difference in primary cilia formation was found when *NRF2*^−/−^ cells were treated with HPI-4, as detected by IF for Ac-Tub and ARL13B (Fig 7A) or immunoblotting for Ac-Tub and ARL13B protein levels (Fig 7B, S12A and S12B Fig). HPI-4–mediated inhibition of Hh signaling is also NRF2-dependent because the ratio of GLIFL/GLIR and GLI luciferase activity were reduced only in *NRF2*^+/+^ cells, not *NRF2*^−/−^ cells (Fig 7B, S12A–S12D Fig). Collectively, these results demonstrate that HPI-4 is a canonical NRF2 inducer, and NRF2 activation is required for HPI-4–mediated suppression of primary ciliogenesis and Hh signaling.

## Discussion

Tumor progression is associated with a shift from normal homeostasis to a protumorigenic phenotype centered on rapid proliferation, metabolism, and survival under harsh conditions. As such, transformation and subsequent cancer cell survival is often associated with hyper- or hypoactivation of key cell signaling cascades, whose controlled activation is integral to normal cellular function. A key example of this phenomenon is hyperactivation of the NRF2 signaling pathway. Controlled activation of NRF2, as a result of increased oxidative or xenobiotic stress, is critical in restoring the redox, proteostatic, and metabolic balance in the cell under stressed conditions. However, multiple cancer types have been found to have hyperactivation of the NRF2 response, conferring not only a growth and survival advantage over their noncancerous counterparts but also resistance to both chemo- and radiotherapies [6]. Thus, understanding the key signaling cascades linking hyperactivation of NRF2 to tumor formation is fundamental in cancer biology.

The Hh signaling pathway controls cell fate and self-renewal and plays a key role in mediating developmental processes. Uncontrolled activation of Hh signaling has been implicated in tumor initiation and progression. SMO and SUFU mutations have been reported in a variety of cancers. Therefore, inhibitors targeting the Hh signaling pathway have been developed, but so far, the clinical outcomes are not promising [43]. Hh signaling is intimately linked to the primary cilium, which is important in regulating cell proliferation, migration, and differentiation. Primary cilia function as a tumor suppressor organelle, and a reciprocal relationship between primary ciliogenesis and cell cycle progression has been reported [44]. In fact, many tumor types are associated with a loss of primary cilia, as well as aberrant Hh signaling [45,46]. Therefore, restoration of primary cilia and activation of Hh signaling should be a viable approach for cancer treatment.

**Fig 8:**
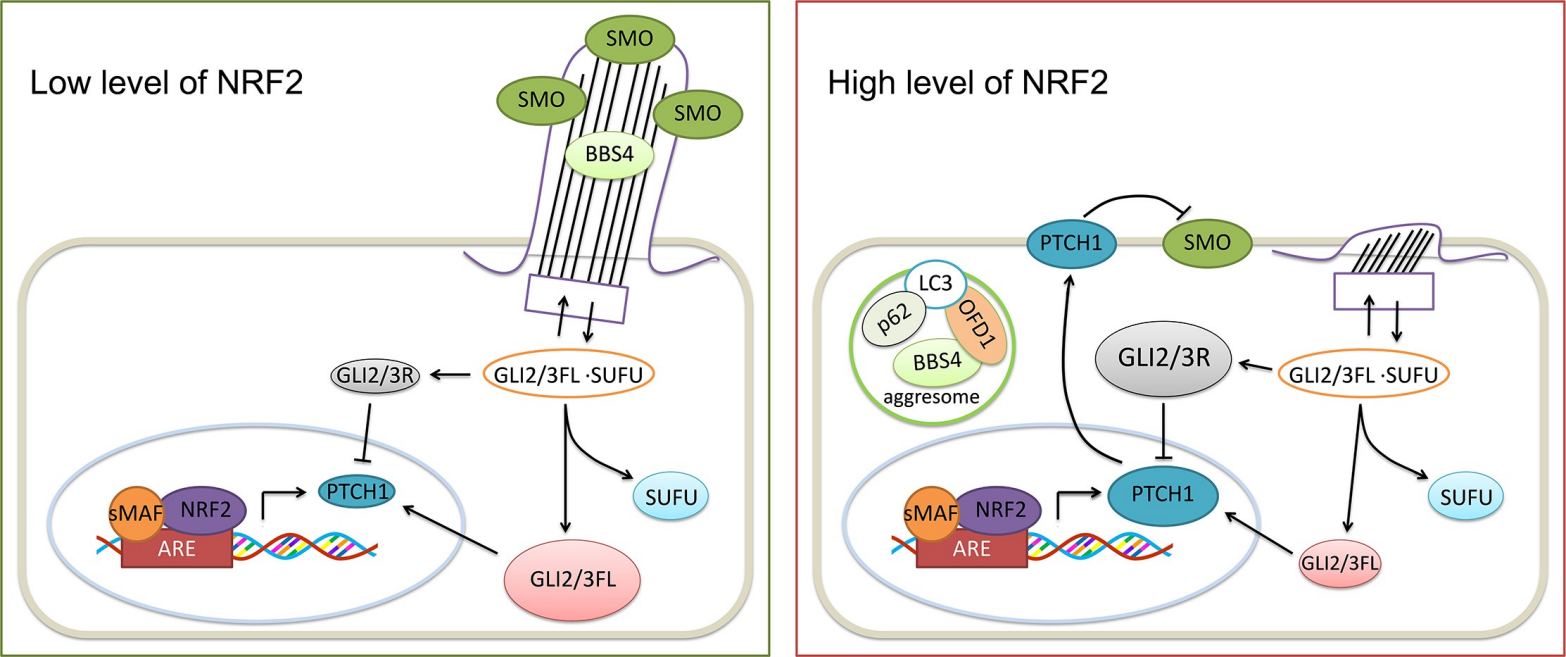
NRF2 suppresses Hh signaling through PTCH1 and primary ciliogenesis via p62. Deletion of NRF2 down-regulated PTCH1, increased primary cilia formation, and activated Hh signaling, all of which can be reversed by NRF2 up-regulation. Moreover, NRF2 suppressed primary cilia formation through p62-dependent aggresome formation and blockage of BBS4 ciliary entrance. ARE, antioxidant response element; BBS4, Bardet–Biedl syndrome 4; FL, full-length activator; Hh, hedgehog; LC3, microtubule-associated proteins 1A/1B light chain 3B; NRF2, nuclear factor-erythroid 2-like 2; OFD1, oral–facial–digital syndrome 1; PTCH1, Patched 1; R, repressor; sMAF, small MAF Transcription Factor; SMO, smoothened; SUFU, suppressor of fused homolog.

In this study, we identified a previously unrecognized role of NRF2 in controlling two key cellular processes, primary ciliogenesis and the Hh signaling pathway, via two distinct mechanisms: (1) PTCH1, a critical negative regulator of Hh signaling, was found to contain a functional ARE, indicating that NRF2 can negatively control Hh signaling through transcriptional up-regulation of PTCH1; and (2) increased expression of p62/SQSTM1, a key autophagy adaptor protein that was previously identified as an NRF2-target gene, promotes aggregation and mislocalization of key proteins controlling ciliogenesis such as OFD1 and BBS4 (Fig 8). Importantly, only simultaneous ablation of both PTCH1 and p62, but neither one alone, was sufficient to prevent the observed suppressive effects of NRF2 on primary cilia and Hh signaling. Since both p62 and PTCH1 are NRF2 target genes, and many cancers have been associated with a loss of primary cilia and aberrant Hh signaling, our data reveal a mechanism by which hyperactivation of NRF2, as seen in lung cancer as well as head and neck cancer, promotes tumor progression via dysregulation of a fundamental cellular organelle: the primary cilium and its associated Hh signaling. This was also confirmed in human lung tumor tissues in which high NRF2 expression strongly correlates with high levels of PTCH1, implicating down-regulation of Hh signaling as a possible mediator of NRF2’s oncogenic effects.

The mechanistic details illustrated here that link NRF2 to primary cilia/Hh signaling are significant for designing cancer therapeutic drugs with a defined mode of action. A small molecule, HPI-4, was originally identified as an Hh pathway inhibitor that acts downstream of SMO in the Hh signaling cascade and interferes with GLI processing and stability [42]. Later on, HPI-4, renamed ciliobrevin A, was reported to disrupt primary cilium formation and GLI-dependent Hh signaling by inhibiting cytoplasmic dynein, an AAA+ ATPase motor protein that is critical in regulating many cellular processes, including ciliary protein trafficking, mitotic spindle formation, and organelle transport through microtubule gliding [35]. Interestingly, we demonstrated here that HPI-4 is a canonical NRF2 inducer, inducing NRF2 in a KEAP1-C151–dependent manner. Moreover, HPI-4–mediated inhibition of primary ciliogenesis and Hh signaling required NRF2 because HPI-4 (ciliobrevin A) had no effect on the percentage of ciliated cells or Hh signaling in *NRF2*^−/−^ cells in our study. Therefore, activation of NRF2 by targeting KEAP1-C151 may be the major effect of HPI-4 on inhibition of primary ciliogenesis and Hh signaling.

There are a few studies that have reported the connection between NRF2 and general ciliation processes. For example, Jang and colleagues reported that increased primary ciliation can induce autophagy, which somehow results in the inactivation of NRF2 and subsequent promotion of neuroectoderm derivation in human embryonic stem cells [47]. Other studies have also indicated that cigarette-smoke–induced mucociliary clearance is preserved in mice treated with the chemical chaperone 4-phenylbutyric acid but that the protective effect was disrupted in mice lacking NRF2, suggesting a key role for NRF2-mediated protection against oxidative stress and altered proteostasis in maintaining proper mucociliary clearance [48]. Moreover, treatment with Manganese(III) (Mn(III)) tetrakis(1-methyl-4-pyridyl) porphyrin, an antioxidant, accelerated the normalization of cilia length concomitant with a decrease of oxidative stress and morphological recovery in the recovery process of damaged kidneys [49]. Therefore, both autophagy and oxidative stress could be the key factors linking NRF2 and ciliation. During the course of generating this publication, a study reporting the opposite of our findings—i.e., that NRF2 positively regulates primary cilia—was also published [50]. In fact, some of their findings support our own work, including the presence of an ARE in PTCH1, as well as NRF2 regulation of ciliogenesis occurring independently of Hh signaling. However, our data indicate negative regulation of ciliogenesis and Hh signaling by NRF2, which is fully supported by our results utilizing pharmacological and genetic manipulation of NRF2 in multiple lung cancer cell lines as well as MEFs. Therefore, it is clear that a relationship among NRF2, ciliogenesis, and Hh signaling exists and still needs further investigation.

Lastly, a better understanding of the crosstalk between NRF2 and primary cilia/Hh signaling not only opens new avenues for cancer therapeutic discovery but also has significant implications regarding pathologies other than cancer, including developmental disorders, in which improper function of these pathways plays a major role. Intriguingly, *Nrf2*^−/−^ mice develop normally, except that these mice are more sensitive to chemical carcinogens, whereas *Keap1*^−/−^ mice die shortly after birth because of hyperkeratosis of the forestomach and esophagus as a result of hyperactivation of the NRF2 pathway [51,52]. Thus, a more detailed analysis of NRF2 expression, ciliation, and the Hh signaling pathway at the different stages of life, from embryogenesis to adulthood, particularly with regards to how this crosstalk changes from ensuring proper development and orchestration of the antistress response to more malignant programming in certain disease contexts, is of critical importance.

## Materials and methods

### Ethics statement

All mice were handled according to the Guide for the Care and Use of Laboratory Animals, and the protocols were approved by the University of Arizona Institutional Animal Care and Use Committee (approval number: 11–287). Our protocols comply with (1) the Animal Welfare Act/Animal Welfare Regulations (AWA/AWRs) and other applicable federal regulations such as GLP for covered species and activities; (2) The National Research Council Guide for the Care and Use of Laboratory Animals, 8th Edition (Guide), for all vertebrate animals used in biomedical research; and (3) The Guide for Care and Use of Agricultural Animals in Research and Teaching, 3rd Edition (Ag Guide), for production farm animal research and teaching. In addition, this study was approved by the Committee on the Ethics of Human Subject Research at the University of Arizona. In this study, the patients who donated their cancer tissues for our research have provided their written informed consent.

### Chemicals and cell culture

HPI-4, bixin, and sodium arsenite (As(III)) were purchased from Sigma-Aldrich (St. Louis, MO, USA), and sulforaphane (SF) was obtained from Santa Cruz Biotechnology (Dallas, TX, USA). Recombinant Human Shh was purchased from PeproTech (Rocky Hill, NJ, USA). MEFs were isolated from *Nrf2* WT (*Nrf2*^+/+^) and knockout (*Nrf2*^−/−^) mice and cultured with DMEM (Corning, Corning, NY, USA) supplemented with 10% FBS (Atlanta Biologicals, Bio-Techne, Minneapolis, MN, USA), 1% L-glutamine (Invitrogen, Carlsbad, CA, USA), 1% Nonessential Amino Acids (Invitrogen), 0.1% beta-mercaptoethanol (Thermo Fisher Scientific, Waltham, MA, USA), and 1% penicillin/streptomycin (Invitrogen). H838 and H1299 were grown in DMEM supplemented with 10% FBS and 1% penicillin/streptomycin. BEAS-2B cells were cultured in Ham’s F-12 medium supplemented with 1% bovine hypothalamus extract (PromoCell, Heidelberg, Germany), insulin (2 mg/ml; Sigma-Aldrich), epidermal growth factor (10 μg/ml; Millipore, Burlington, MA, USA), transferrin (2.5 mg/ml; Sigma-Aldrich), cholera toxin (10 μg/ml; List Biological Laboratories, Inc., Campbell, CA, USA), and dexamethasone (0.05 mM; Sigma-Aldrich). For primary cilia analysis, cells were cultured with DMEM containing 0.5% FBS and 1% penicillin/streptomycin for 48 h. All cells were cultured at 37°C in a humidified incubator containing 5% CO2.

### Generation of *NRF2*^−/−^, *KEAP1*^−/−^, *PTCH1*^−/−^, and *p62*/*SQSTM1*^−/−^ cells

NRF2 knockout (*NRF2*^−/−^), KEAP1 knockout (*KEAP1*^−/−^), PTCH1 knockout (*PTCH*1^−/−^), and p62/SQSTM1 knockout (*p62*/*SQSTM1*^−/−^) cells were generated using CRISPR-Cas9–mediated gene editing [53–55]. A pair of single guide RNA (sgRNA) sequences was used to target coding sequences near the promoter region of each gene of interest. The sgRNA sequences are as follows:

NRF2: sgRNA-A 5′-TATTTGACTTCAGTCAGCGA-3′

        sgRNA-B 5′-TAGTTGTAACTGAGCGAAAA-3′

KEAP1: sgRNA-A 5′-AGCGTGCCCCGTAACCGCAT-3′

        sgRNA-B 5′-GATCTACACCGCGGGCGGCT-3′

PTCH1: sgRNA-A 5′-TGCACTCCGCCGAAAGCCTC-3′

        sgRNA-B 5′-AGCGAACCTCGAGACCAACG-3′

p62: sgRNA-A 5′-AATGGCCATGTCCTACGTGA-3′

        sgRNA-B 5′-CGACTTGTGTAGCGTCTGCG-3′

Each sgRNA pair was annealed and then ligated into the pSpCas9(BB)-2A-GFP plasmid. Cells were then cotransfected with 1 μg of the pSpCas9(BB)-2A-GFP plasmid carrying sgRNA-A and 1 μg of the pSpCas9(BB)-2A-GFP plasmid carrying sgRNA-B. GFP-positive cells were isolated using FACS and subsequently plated at a low confluence for colony formation and isolation. Once colonies were obtained, individual clones were expanded, and the successful homozygous knockout of the target genes of interest was confirmed by western blot (WB). Finally, generation of *NRF2*^−/−^, *KEAP1*^−/−^, *PTCH1*^−/−^ and *p62*/*SQSTM1*^−/−^ cell lines was confirmed by detecting loss of protein expression via immunoblotting.

### Construction of recombinant DNA molecules

The NRF2 overexpression vector was constructed by cloning a PCR-generated portion of the NRF2 coding sequence into the pCI vector (Promega, Madison, WI, USA). For the dual luciferase assay, the portion of the human *PTCH1* promoter and mouse *Ptch1* promoter containing the putative ARE sequence was then amplified by PCR, and the amplified fragments were cloned into the pGL4.22 vector (Promega). The pGL3-8XGliBS:Luc, ptf-LC3 (mRFP-GFP-LC3), pcDNA-KEAP1-WT, and pcDNA-KEAP1-C151S plasmids were generated as described previously [56,57].

### Transfection of cDNA and luciferase reporter assay

Transfection of cDNA was performed using the Lipofectamine 3000 reagent (Invitrogen) according to the manufacturer’s instructions. Luciferase activity was measured using the dual luciferase reporter assay system (Promega). For relative luciferase activity analysis, the value of Firefly-luciferase was normalized to the value of Renilla luciferase. The experiment was repeated 3 times. The data are expressed as means ± SD.

### mRNA extraction and real-time qRT-PCR

Total mRNA was extracted using TRIzol (Invitrogen) according to the manufacturer’s instructions. cDNA was then synthesized using 2 μg of mRNA and a Transcriptor first-strand cDNA synthesis kit (Promega). Real-time qPCR was then performed as previously described [55]. β-actin was used for qPCR normalization, and all experiments were measured in triplicate. Primer sequences (5′-3′) are as follows:

Mouse-*Ift20*-Forward 5′-AGAAGCAGAGAACGAGAAGATG-3′

Mouse-*Ift20-*Reverse 5′-CACAAAGCTTCATATTCAACCCG-3′

Mouse-*Ift88*-Forward 5′-TGAGGACGACCTTTACTCTGG-3′

Mouse-*Ift88*-Reverse 5′-CTGCCATGACTGGTTCTCACT-3′

Mouse-*Kif3a*-Forward 5′-ATGCCGATCAATAAGTCGGAGA -3′

Mouse-*Kif3a*-Reverse 5′-GTTCCCCTCATTTCATCCACG-3′

Mouse-*Ptch1*-Forward 5′-CCGTTCAGCTCCGCACAGA-3′

Mouse-*Ptch1*-Reverse 5′-CTCACTCGGGTGGTCCCATAAA-3′

Mouse-*β-actin*-Forward 5′-AAGGCCAACCGTGAAAAGAT-3′

Mouse-*β-actin*-Reverse 5’-GTGGTACGACCAGAGGCATAC-3’

Human-*IFT20*-Forward 5′-GCAGCAACTTCAAGCCCTAAT-3′

Human-*IFT20*-Reverse 5′-ACGCCACCTCTTGTGACATAG-3′

Human-*IFT88*-Forward 5′-GCCGAAGCACTTAACACTTAT-3′

Human-*IFT88*-Reverse 5′-GTCTAATGCCATTCGGTAGAA-3′

Human-*KIF3a*-Forward 5′-GAGGAGAGTCTGCGTCAGTCT-3′

Human-*KIF3a*-Reverse 5′-CAGGCTTTGCAGAACGCTTTC-3′

Human-*PTCH1*-Forward 5′-CCAGAAAGTATATGCACTGGCA-3′

Human-*PTCH1*-Reverse 5′-GTGCTCGTACATTTGCTTGGG-3′

Human-*β-actin*-Forward 5′-CCCAGAGCAAGAGAGG-3′

Human-*β-actin*-Reverse 5′-GTCCAGACGCAGGATG-3′

### WB, IF, and immunohistochemical (IHC) analysis

WB, IF, and IHC were performed as previously described [55,58,59]. Primary antibodies against NRF2 (1:1,000 for WB, 1:200 for IF and IHC, Cat# sc-13032), SMO (1:1,000 for WB, 1:200 for IF, Cat# sc-13943), PTCH1 (1:1,000 for WB, 1:200 for IHC, Cat# sc-9016), KEAP1 (1:1,000 for WB, Cat# sc-15246), Glutamate-Cysteine Ligase Modifier Subunit (GCLM) (1:1,000 for WB, Cat# sc-55586), GLI2 (1:1,000 for WB, Cat# sc-28674), IFT-20 (1:1,000 for WB, Cat# sc-51718), KIF3a (1:1,000 for WB, Cat# sc-376680), and glyceraldehyde-3-phosphate dehydrogenase (GAPDH) (1:3,000 for WB, Cat# sc-32233), as well as horseradish peroxidase (HRP)-conjugated secondary antibodies (1:3,000 for WB, Cat# sc-2350, sc-2004, sc-2005), were purchased from Santa Cruz Biotechnology. The antibody against Ac-Tub (1:3,000 for WB, 1:800 for IF, Cat# T7451) was purchased from Sigma-Aldrich. The antibodies against ARL13B (1:3,000 for WB, 1:500 for IF, Cat# 17711-1-AP), NDE1 (1:100 for IF, Cat# 10233-1-AP), and IFT-88 (1:3,000 for WB, Cat# 13967-1-AP) were purchased from Proteintech (Rosemont, IL, USA). The antibody against Aurora A (1:100 for IF, Cat# 12100S) and Phospho-Aurora A (Thr288) (1:100 for IF, Cat# C39D8) was purchased from Cell Signaling Technology (Danver, MA, USA). The antibody against GLI3 (1:3,000 for WB, Cat# AF3690) was purchased from R&D (Bio-Techne). The antibody against OFD1 (1:4,000 for WB, 1:1,000 for IHC, Cat# ab222837) and BBS4 (1:1,000 for WB, 1:200 for IHC, Cat# ab188364) was purchased from Abcam (Cambridge, UK). The Alexa-Fluor-488–conjugated secondary antibody (1:2,000 for IF, Cat# A10254 and A32731) and Alexa-Fluor-594–conjugated secondary antibody (1:2,000 for IF, Cat# A11037 and A32742) were obtained from Invitrogen. All of uncropped blots throughout the paper were shown in S1 Raw images.

### Biotin-DNA pull-down

Biotin-DNA pull-down was performed as reported previously [60]. In brief, cells were lysed in RIPA buffer containing 1 mM DTT, 1 mM phenylmethylsulfonyl fluoride (PMSF), and 1% protease inhibitor cocktail (Sigma-Aldrich). The cell lysates were precleared with streptavidin beads and incubated with 2 μg biotinylated DNA probes that spanned the ARE-containing sequences in the promoter regions of both human *PTCH1* and mouse *Ptch1*. The DNA–protein complexes were further pulled down by streptavidin beads, and complexes were washed 3 times, resolved on an SDS-PAGE gel, and subjected to immunoblot analysis. The sequences of the 41-bp biotinylated DNA probes used are as follows:

Human WT PTCH1-ARE probe:

5′-TTCTGGAAACTCAAATGACTCTGCTCAAGAATGGCTACGTC-3′

Human mutant PTCH1-ARE probe:

5′-TTCTGGAAACTCAAAACTCTCTCGTCAAGAATGGCTACGTC-3′

Mouse WT PTCH1-ARE probe:

5′-TCTTTTCTTCAGTTATGACTCAGAATCCAGTGTTTGGCTAA-3′

Mouse mutant PTCH1-ARE probe:

5′-TCTTTTCTTCAGTTAACTCTCACTATCCAGTGTTTGGCTAA-3′

### Statistical analysis

Results are presented as means ± SD for at least 3 independent experiments. Statistical analysis was performed using SPSS 17.0. Unpaired Student *t* tests were applied to compare the means for two groups. One-way ANOVA with Bonferroni’s correction was used to compare the means of 3 or more groups. *p* < 0.05 was considered statistically significant. The values for the data used to create the graphs throughout the paper are shown in S1 Data.

## Supporting information

S1 Fig NRF2 deletion enhances ciliogenesis and Hh signaling (related to Fig 1). (A–C) Relative quantification of immunoblot results in Fig 1A, 1B and 1E. (D) The normalized result of Fig 1F. The level of relative luciferase activity in all control groups (both *NRF2*^+/+^ cells and *NRF*2^−/−^ cells) was considered as “1.” Results are expressed as mean ± SD. A *t* test was used to compare the various groups, and *p* < 0.05 was considered statistically significant. *p < 0.05 compared between the two groups. Hh, hedgehog; NRF2, nuclear factor-erythroid 2-like 2
(PDF)

S2 Fig Effect of NRF2 deletion on CDC components. IF for Ac-Tub (red)/NDE1 (green), Ac-Tub (red)/OFD1 (green), ARL13B (red)/Aurora A (green), and Ac-Tub (red)/pAurora A (T288) (green) in *NRF2*^+/+^ and *NRF2*^−/−^ MEFs, BEAS-2B and H838 cell lines. The percentage of cilia with localization of CDC components was calculated in the different groups. (Scale bar = 5 μm, *n* = 150.) Results are expressed as mean ± SD. A *t* test was used to compare the various groups, and *p* < 0.05 was considered statistically significant. *p < 0.05 compared between the two groups. Ac-Tub, acetylated tubulin; ARL13B, ADP-ribosylation factor-like protein 13B; CDC, cilium disassembly complex; IF, immunofluorescence; MEF, mouse embryonic fibroblast; NDE1, NudE Neurodevelopment Protein 1; NRF2, nuclear factor-erythroid 2-like 2
(PDF)

S3 Fig NRF2 activation inhibits Hh signaling, ciliogenesis, and ciliary translocation of SMO (related to Fig 2). (A–B) Relative quantification of immunoblot results in Fig 2A and 2B. Results are expressed as mean ± SD. A *t* test was used to compare the various groups, and *p* < 0.05 was considered statistically significant. *p < 0.05 compared with the control group. Hh, hedgehog; NRF2, nuclear factor-erythroid 2-like 2; SMO, smoothened.
(PDF)

S4 Fig Effect of bixin treatment in *NRF2*^+/+^ and *NRF2*^−/−^ cells. (A–B) *NRF2*^+/+^ and *NRF2*^−/−^ H1299 cells were treated with bixin (40 μM) for 0, 12, 24, 36, or 48 h and subjected to immunoblot analysis of key Hh and ciliary proteins. Relative quantification of immunoblot results is shown in S4C and S4D Fig. (E) GLI luciferase assay in *NRF2*^+/+^ and *NRF2*^−/−^ H1299 cells treated with bixin for 48 h. (F–G) Bixin-treated (40 μM for 48 h) *NRF2*^+/+^ and *NRF2*^−/−^ H1299 cells were subjected to IF analysis of (F) percent ciliated cells or (G) colocalization of Ac-Tub (green) and SMO (red) (D: scale bar = 10 μm; E: scale bar = 5 μm). Results are expressed as mean ± SD. A *t* test was used to compare the various groups, and *p* < 0.05 was considered statistically significant. *p < 0.05 compared with the control group. Ac-Tub, acetylated tubulin; Hh, hedgehog; IF, immunofluorescence; NRF2, nuclear factor-erythroid 2-like 2; SMO, smoothened.
(PDF)

S5 Fig Effect of NRF2 overexpression on cell cycle. *KEAP1*^−/−^, bixin-treated (40 μM for 48 h), and pCI-NRF2–transfected (for 48 h) H1299 cells were subjected to PI staining and FACS. The percentage of G0/G1, S, and G2/M phase cells was calculated. Results are expressed as mean ± SD. A *t* test was used to compare the various groups, and *p* < 0.05 was considered statistically significant. *p < 0.05 compared with the control group. FACS, fluorescence-activated cell sorting; KEAP1, Kelch-like ECH-associated protein 1; NRF2, nuclear factor-erythroid 2-like 2; PI, propidium iodide.
(PDF)

S6 Fig PTCH1 is a target gene of NRF2 (related to Fig 3). (A) 41-bp sequence containing ARE and flanking regions in human and mouse PTCH1. The ARE sequence is underlined with critical conserved nucleotides indicated in red. (B–C) *PTCH1*-ARE luciferase assay in *NRF2*^+/+^ and *NRF2*^−/−^ H838 (B) and MEF (C) cells. (D–E) Relative quantification of immunoblot results in Fig 3C and 3D, respectively. (F) Representative IHC images and a linear regression analysis indicating the correlation between NRF2 and PTCH1 expression in human lung cancer tissues (scale bar = 30 μm). Results are expressed as mean ± SD. A *t* test was used to compare the various groups, and *p* < 0.05 was considered statistically significant. *p < 0.05 compared between the two groups. ARE, antioxidant response element; IHC, immunohistochemical; MEF, mouse embryonic fibroblast; NRF2, nuclear factor-erythroid 2-like 2; PTCH1, Patched 1
(PDF)

S7 Fig PTCH1 is required for NRF2-mediated inhibition of ciliary translocation of SMO, but not the suppression of primary ciliogenesis by NRF2 (related to Fig 4). (A–C) Relative quantification of immunoblot results in Fig 4A, 4C and 4D. Results are expressed as mean ± SD. A *t* test was used to compare the various groups, and *p* < 0.05 was considered statistically significant. *p < 0.05 compared with the control group. NRF2, nuclear factor-erythroid 2-like 2; PTCH1, Patched 1; SMO, smoothened.
(PDF)

S8 Fig NRF2 inhibits primary ciliogenesis by increasing p62-dependent inclusion body formation and suppressing the ciliary entrance of BBS4 (related to Fig 5). (A–C) Relative quantification of immunoblot results in Fig 5A, 5B and 5C. (D). Effect of bixin treatment in *NRF2*^+/+^ and *NRF2*^−/−^ H1299 cells. (E) Relative quantification of immunoblot results in Fig 5E. Results are expressed as mean ± SD. A *t* test was used to compare the various groups, and *p* < 0.05 was considered statistically significant. *p < 0.05 compared with the control group. BBS4, Bardet–Biedl syndrome 4; NRF2, nuclear factor-erythroid 2-like 2.
(PDF)

S9 Fig Bixin enhances inclusion body formation in a p62-dependent manner. (A) *PTCH1*^+/+^ and *PTCH1*^−/−^ H1299 cells were transfected with mRFP-GFP-LC3 for 24 h and then treated with bixin (40 μM) for 4, 24, and 48 h and imaged. (B) *p62*/*SQSTM1*^+/+^ and *p62*/*SQSTM1*^−/−^ H1299 cells were transfected with mRFP-GFP-LC3 for 24 h and then treated with bixin (40 μM) for 4, 24, and 48 h and imaged. Yellow puncta = LC3-positive autophagosomes/inclusion bodies. (Scale bar = 5 μm.) GFP, green fluorescent protein; LC3, microtubule-associated proteins 1A/1B light chain 3B; mRFP, monomeric red fluorescent protein; PTCH1, Patched 1; SQSTM1, sequestosome 1.
(PDF)

S10 Fig Hh signaling is not regulated by NRF2 in *PTCH1*^−/−^;p62-KD cells (related to Fig 6). (A–B) Relative quantification of immunoblot results in Fig 6A and 6B. Results are expressed as mean ± SD. A *t* test was used to compare the various groups, and *p* < 0.05 was considered statistically significant. *p < 0.05 compared with the control group. Hh, hedgehog; KD, knockdown; NRF2, nuclear factor-erythroid 2-like 2; PTCH1, Patched 1.
(PDF)

S11 Fig HPI-4 induces NRF2 through the canonical pathway. (A) Immunoblot analysis of the effect of HPI-4 treatment on H1299 *KEAP1*^+/+^ and H1299 *KEAP1*^−/−^ cells treated with HPI-4 for 0, 12, 24, 36, or 48 h. (B) An H1299 *KEAP1*^−/−^ cell line was transfected with plasmids encoding mGST-ARE-luciferase and TK-Renilla luciferase, along with a plasmid for KEAP1-WT or KEAP1-C151S. Following transfection for 48 h, cells were treated with 20 μM HPI-4, 40 μM bixin, or 1 μM sodium arsenite (As) for 16 h and harvested for luciferase activity detection and immunoblot assay. Results are expressed as mean ± SD. A *t* test was used to compare the various groups, and *p* < 0.05 was considered statistically significant. *p < 0.05 compared with the control group. ARE, antioxidant response element; HPI-4, hedgehog pathway inhibitor-4; KEAP1, Kelch-like ECH-asosciated protein 1; mGST, mouse glutathione S-transferase; NRF2, nuclear factor-erythroid 2-like 2; TK, thymidine kinase; WT, wild type.
(PDF)

S12 Fig HPI-4 inhibits the formation of primary cilia in an NRF2-dependent manner (related to Fig 7). (A–B) Relative quantification of immunoblot results in Fig 7B. (C–D) GLI luciferase assay in *NRF2*^+/+^ and *NRF2*^−/−^ MEF (C) and H838 (D) cells treated with HPI-4 for 48 h. Results are expressed as mean ± SD. A *t* test was used to compare the various groups, and *p* < 0.05 was considered statistically significant. *p < 0.05 compared with the control group. HPI-4, hedgehog pathway inhibitor-4; MEF, mouse embryonic fibroblast; NRF2, nuclear factor-erythroid 2-like 2.
(PDF)

S1 Raw Images Uncropped blots shown throughout the paper. (PDF)

S1 Data Values for all data used to create the graphs throughout the paper. (XLSX)

## Abbreviations

Ac-Tub: acetylated tubulin
ARE: antioxidant response element
ARL13B: ADP-ribosylation factor-like protein 13B
AS(III): sodium arsenite
AWA/AWR: Animal Welfare Act/Animal Welfare Regulations
BBS4: Bardet–Biedl syndrome 4
B-Raf: v-raf murine sarcoma viral oncogene homolog B1
CDC: cilium disassembly complex
Cul3: Cullin 3
FACS: fluorescence-activated cell sorting
FL: full-length activator
GAPDH: glyceraldehyde-3-phosphate dehydrogenase
GFP: green fluorescent protein
GCLM: Glutamate-Cysteine Ligase Modifier Subunit
Hh: hedgehog
HPI-4: hedgehog pathway inhibitor-4
HRP: horseradish peroxidase
IF: immunofluorescence
IFT: intraflagellar transport
KD: knockdown
KEAP1: Kelch-like ECH-associated protein 1
KIF3a: Kinesin Family Member 3A
K-Ras: Ki-ras2 Kirsten rat sarcoma viral oncogene homolog
LC3: microtubule-associated proteins 1A/1B light chain 3B
MEF: mouse embryonic fibroblast
Mn(III): Manganese(III)
mRFP: monomeric red fluorescent protein
MU: mutation
Myc: MYC Proto-Oncogene
NDE1: NudE Neurodevelopment Protein 1
NQO1: NAD(P)H Quinone Dehydrogenase 1
NRF2: nuclear factor-erythroid 2-like 2
OFD1: oral–facial–digital syndrome 1
PI: propidium iodide
PMSF: phenylmethylsulfonyl fluoride
PTCH1: Patched 1
qRT-PCR: Real-Time Quantitative Reverse Transcription PCR
R: repressor
Rbx1: Ring-Box 1
ROS: reactive oxygen species
SF: sulforaphane
sgRNA: single guide RNA
Shh: Sonic hedgehog
siRNA: small interfering RNA
sMAF: small MAF Transcription Factor
SMO: smoothened
SQSTM1: sequestosome 1
SUFU-GLI: suppressor of fused homolog-zinc finger protein-GLI
TP53: tumor protein 53
Ub: ubiquitination
WT: wild type
