# Supplementary figures and images for: NRF2 negatively regulates primary ciliogenesis and hedgehog signaling
Source: PLoS Biol. 2020 Feb 13;18(2):e3000620. doi: 10.1371/journal.pbio.3000620 (PMC7043785; doi:10.1371/journal.pbio.3000620)

S1 Fig

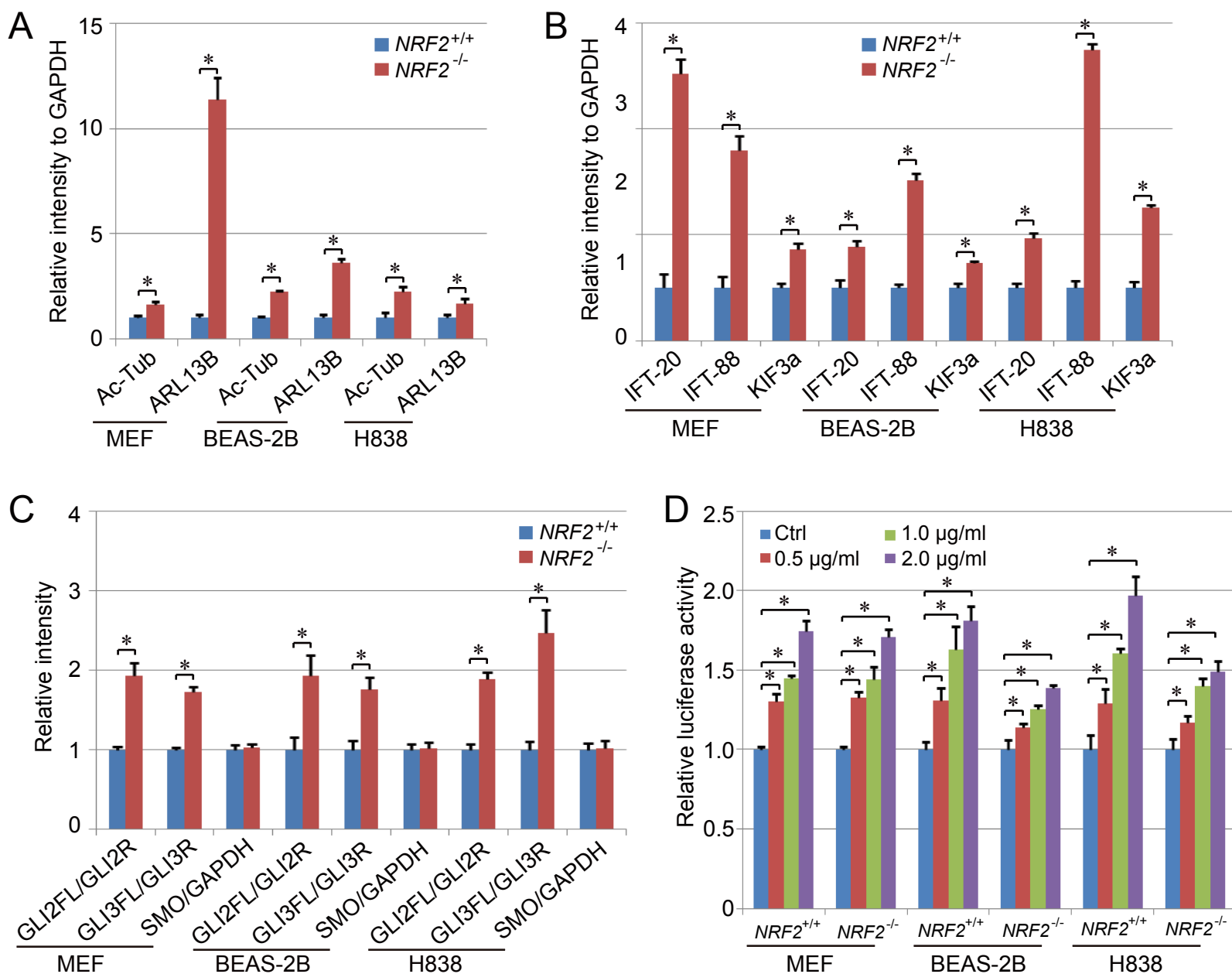

Supplement: S1 Fig — (A–C) Relative quantification of immunoblot results in Fig 1A, 1B and 1E. (D) The normalized result of Fig 1F. The level of relative luciferase activity in all control groups (both NRF2+/+ cells and NRF2−/− cells) was considered as “1.” Results are expressed as mean ± SD. A t test was used to compare the various groups, and p < 0.05 was considered statistically significant. *p < 0.05 compared between the two groups. Hh, hedgehog; NRF2, nuclear factor-erythroid 2-like 2 (PDF) [file pbio.3000620.s001.pdf]

S2 Fig

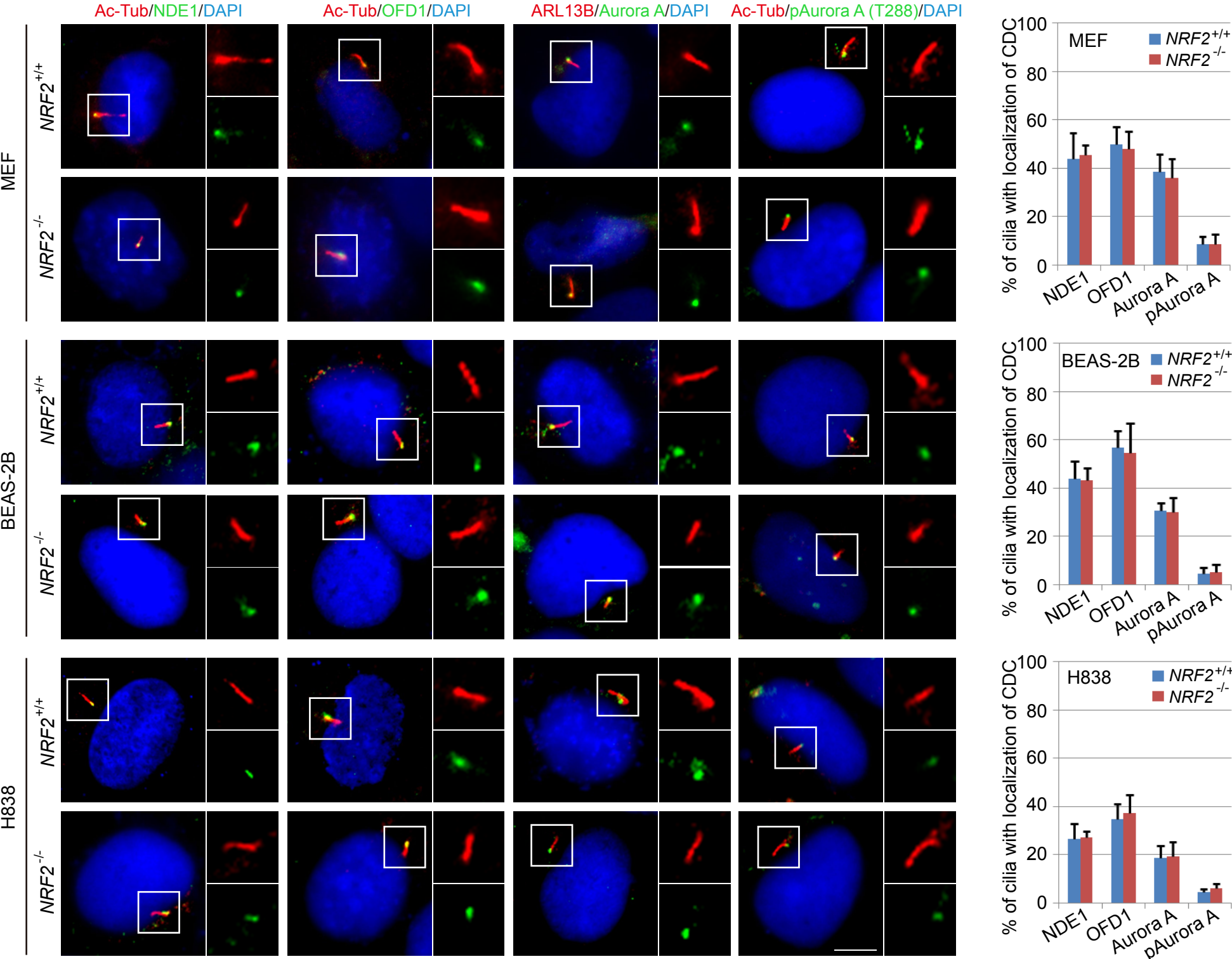

Supplement: S2 Fig — IF for Ac-Tub (red)/NDE1 (green), Ac-Tub (red)/OFD1 (green), ARL13B (red)/Aurora A (green), and Ac-Tub (red)/pAurora A (T288) (green) in NRF2+/+ and NRF2−/− MEFs, BEAS-2B and H838 cell lines. The percentage of cilia with localization of CDC components was calculated in the different groups. (Scale bar = 5 μm, n = 150.) Results are expressed as mean ± SD. A t test was used to compare the various groups, and p < 0.05 was considered statistically significant. *p < 0.05 compared between the two groups. Ac-Tub, acetylated tubulin; ARL13B, ADP-ribosylation factor-like protein 13B; CDC, cilium disassembly complex; IF, immunofluorescence; MEF, mouse embryonic fibroblast; NDE1, NudE Neurodevelopment Protein 1; NRF2, nuclear factor-erythroid 2-like 2 (PDF) [file pbio.3000620.s002.pdf]

# S3 Fig

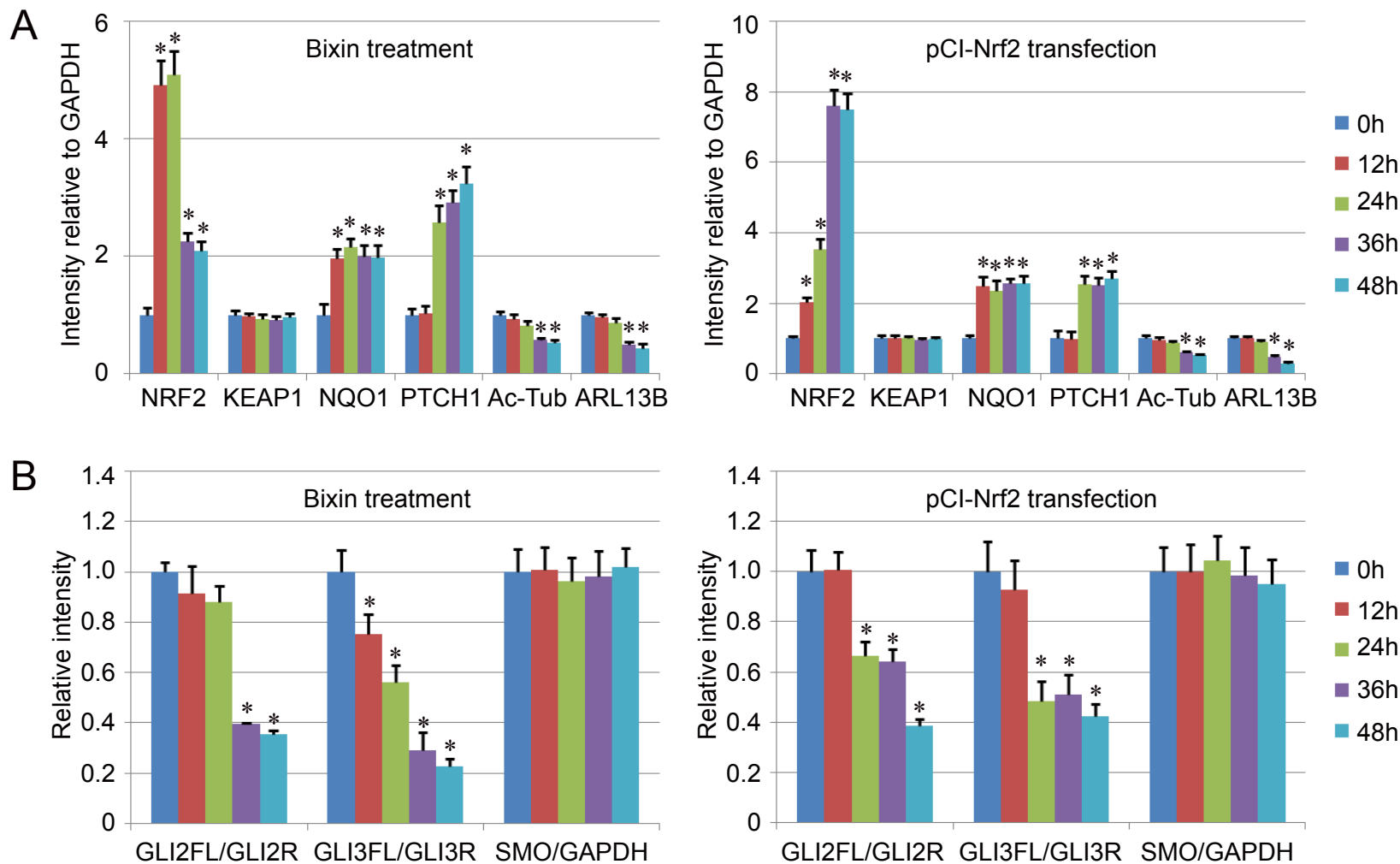

Supplement: S3 Fig — (A–B) Relative quantification of immunoblot results in Fig 2A and 2B. Results are expressed as mean ± SD. A t test was used to compare the various groups, and p < 0.05 was considered statistically significant. *p < 0.05 compared with the control group. Hh, hedgehog; NRF2, nuclear factor-erythroid 2-like 2; SMO, smoothened. (PDF) [file pbio.3000620.s003.pdf]

S4 Fig

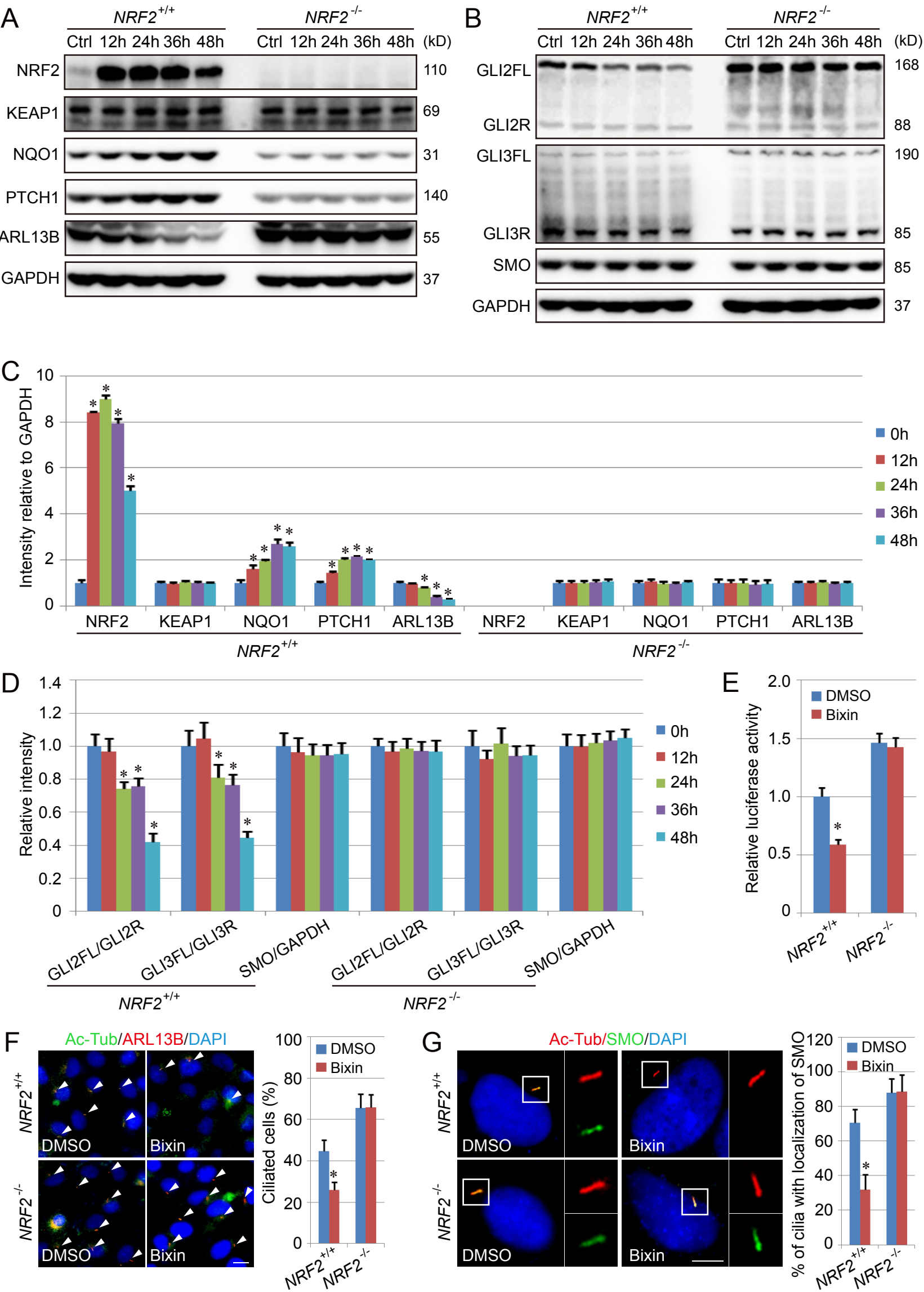

Supplement: S4 Fig — (A–B) NRF2+/+ and NRF2−/− H1299 cells were treated with bixin (40 μM) for 0, 12, 24, 36, or 48 h and subjected to immunoblot analysis of key Hh and ciliary proteins. Relative quantification of immunoblot results is shown in S4C and S4D Fig. (E) GLI luciferase assay in NRF2+/+ and NRF2−/− H1299 cells treated with bixin for 48 h. (F–G) Bixin-treated (40 μM for 48 h) NRF2+/+ and NRF2−/− H1299 cells were subjected to IF analysis of (F) percent ciliated cells or (G) colocalization of Ac-Tub (green) and SMO (red) (D: scale bar = 10 μm; E: scale bar = 5 μm). Results are expressed as mean ± SD. A t test was used to compare the various groups, and p < 0.05 was considered statistically significant. *p < 0.05 compared with the control group. Ac-Tub, acetylated tubulin; Hh, hedgehog; IF, immunofluorescence; NRF2, nuclear factor-erythroid 2-like 2; SMO, smoothened. (PDF) [file pbio.3000620.s004.pdf]

S5 Fig

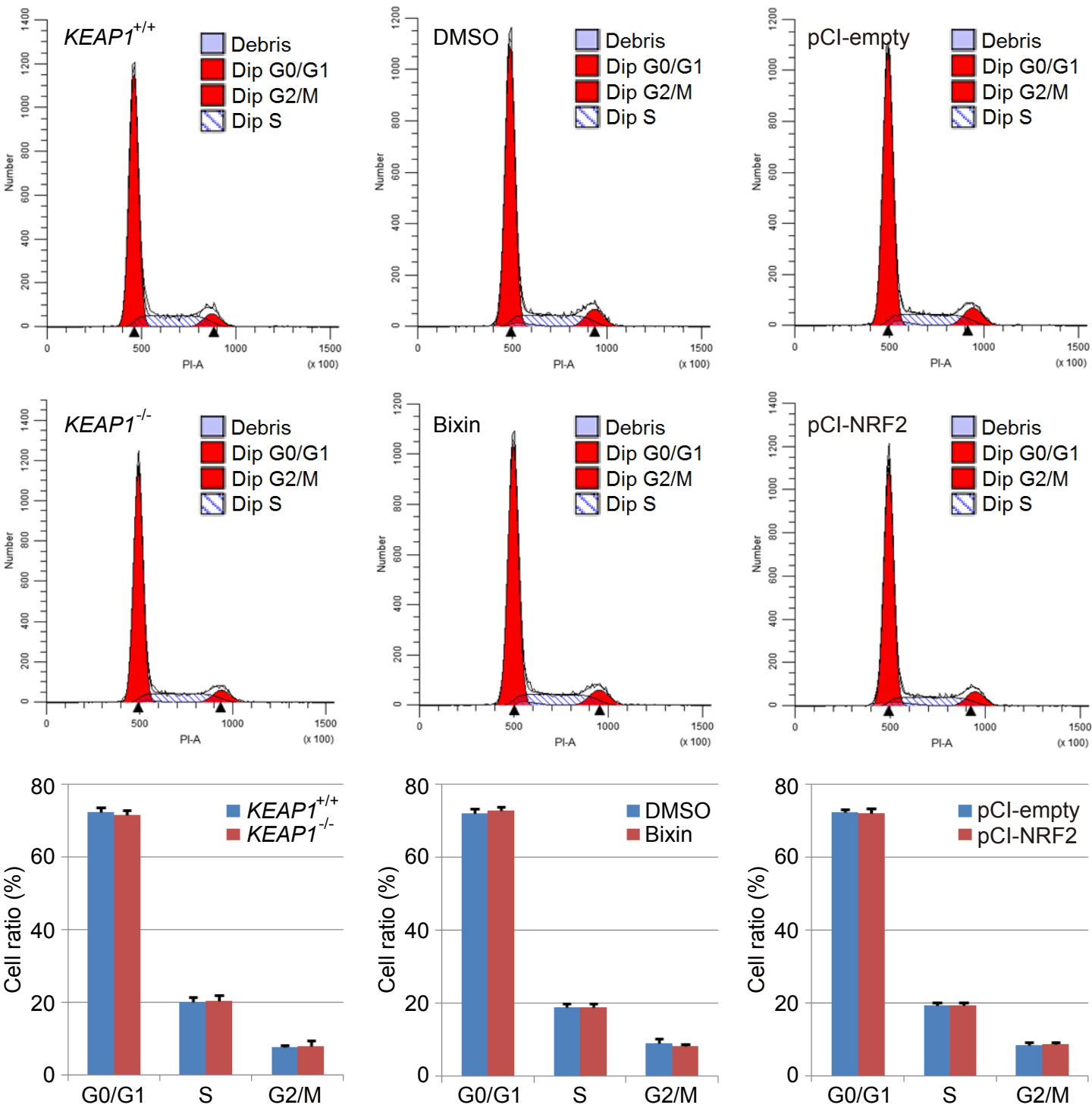

Supplement: S5 Fig — KEAP1−/−, bixin-treated (40 μM for 48 h), and pCI-NRF2–transfected (for 48 h) H1299 cells were subjected to PI staining and FACS. The percentage of G0/G1, S, and G2/M phase cells was calculated. Results are expressed as mean ± SD. A t test was used to compare the various groups, and p < 0.05 was considered statistically significant. *p < 0.05 compared with the control group. FACS, fluorescence-activated cell sorting; KEAP1, Kelch-like ECH-associated protein 1; NRF2, nuclear factor-erythroid 2-like 2; PI, propidium iodide. (PDF) [file pbio.3000620.s005.pdf]

S6 Fig

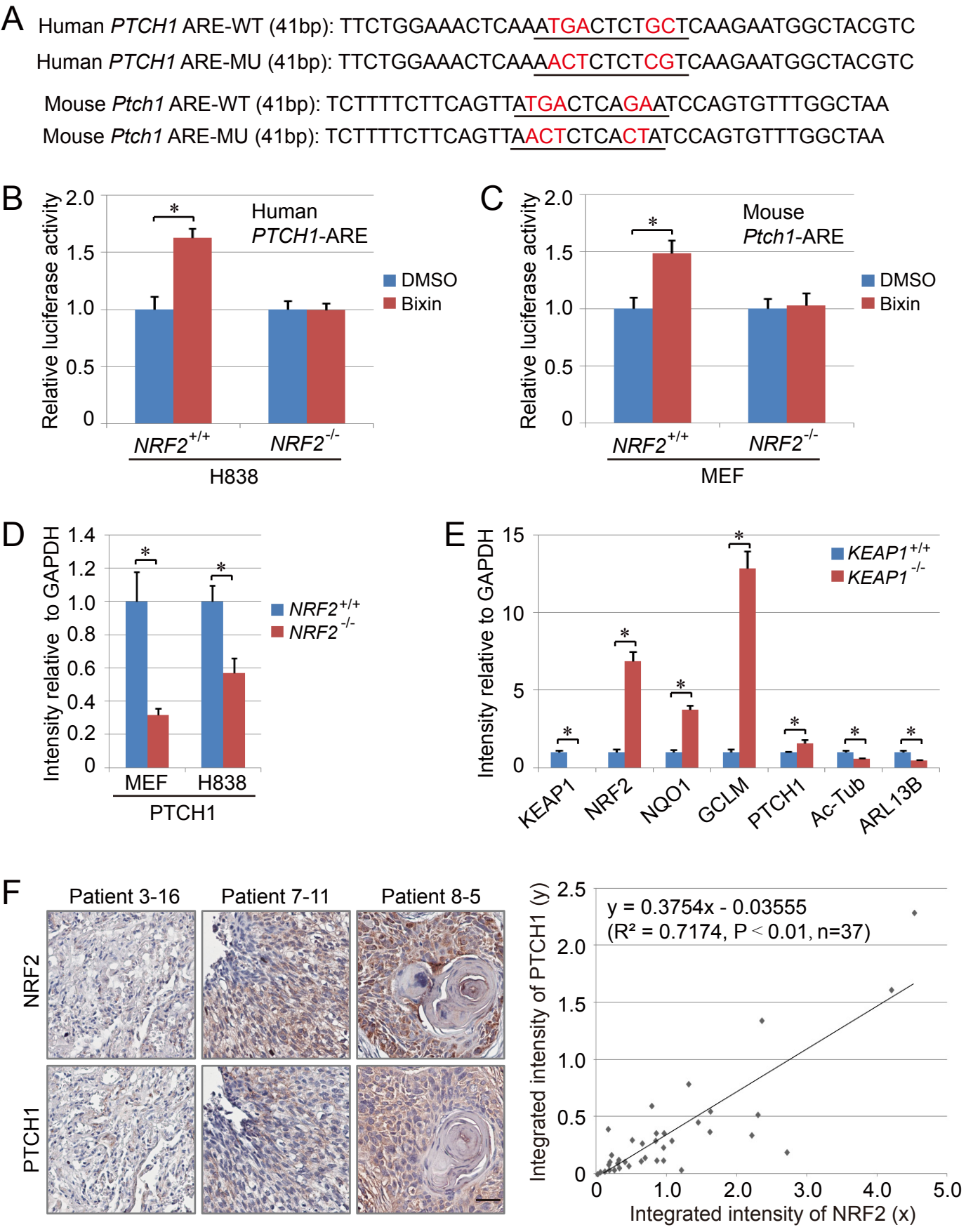

Supplement: S6 Fig — (A) 41-bp sequence containing ARE and flanking regions in human and mouse PTCH1. The ARE sequence is underlined with critical conserved nucleotides indicated in red. (B–C) PTCH1-ARE luciferase assay in NRF2+/+ and NRF2−/− H838 (B) and MEF (C) cells. (D–E) Relative quantification of immunoblot results in Fig 3C and 3D, respectively. (F) Representative IHC images and a linear regression analysis indicating the correlation between NRF2 and PTCH1 expression in human lung cancer tissues (scale bar = 30 μm). Results are expressed as mean ± SD. A t test was used to compare the various groups, and p < 0.05 was considered statistically significant. *p < 0.05 compared between the two groups. ARE, antioxidant response element; IHC, immunohistochemical; MEF, mouse embryonic fibroblast; NRF2, nuclear factor-erythroid 2-like 2; PTCH1, Patched 1 (PDF) [file pbio.3000620.s006.pdf]

S7 Fig

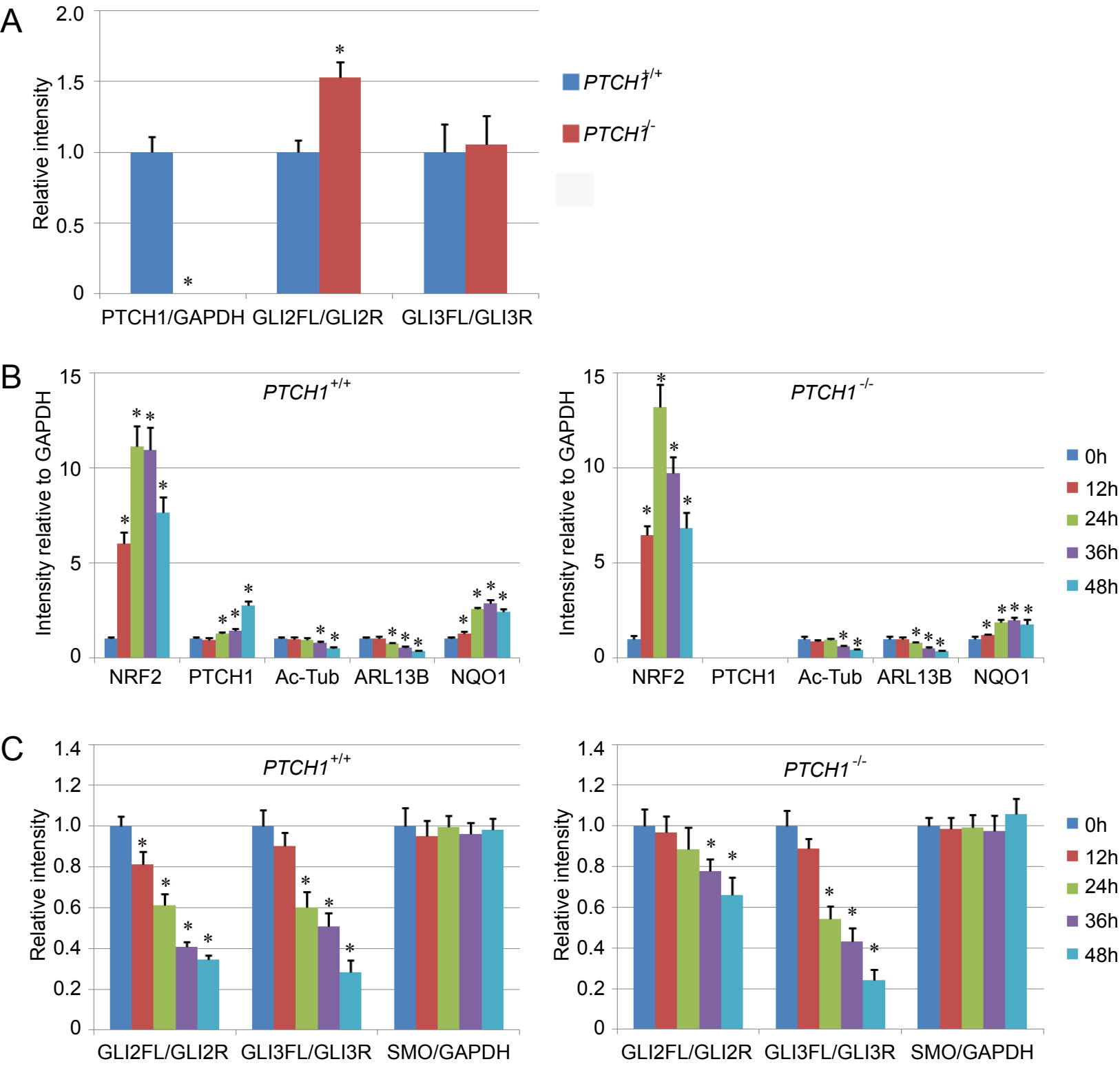

Supplement: S7 Fig — (A–C) Relative quantification of immunoblot results in Fig 4A, 4C and 4D. Results are expressed as mean ± SD. A t test was used to compare the various groups, and p < 0.05 was considered statistically significant. *p < 0.05 compared with the control group. NRF2, nuclear factor-erythroid 2-like 2; PTCH1, Patched 1; SMO, smoothened. (PDF) [file pbio.3000620.s007.pdf]

S8 Fig

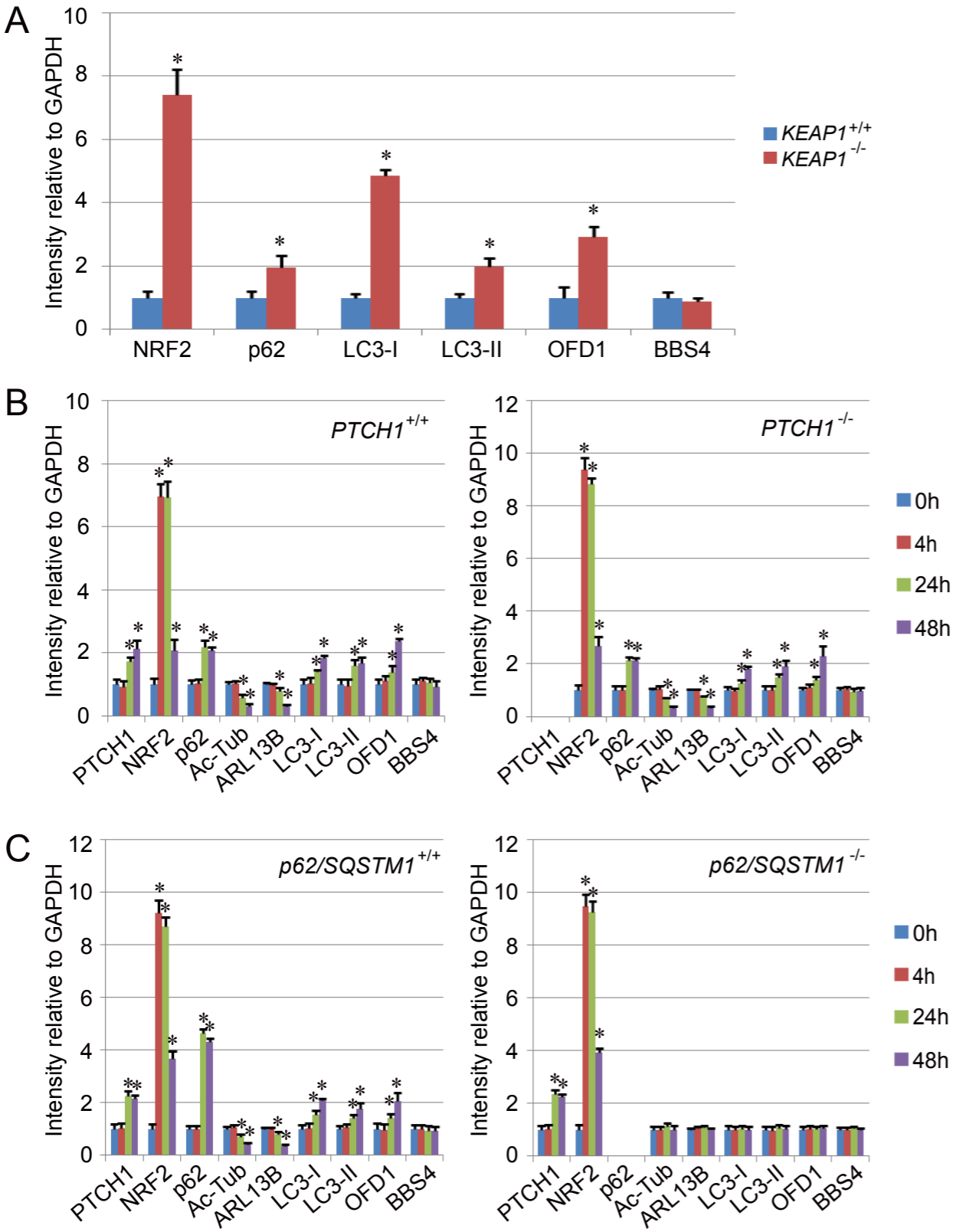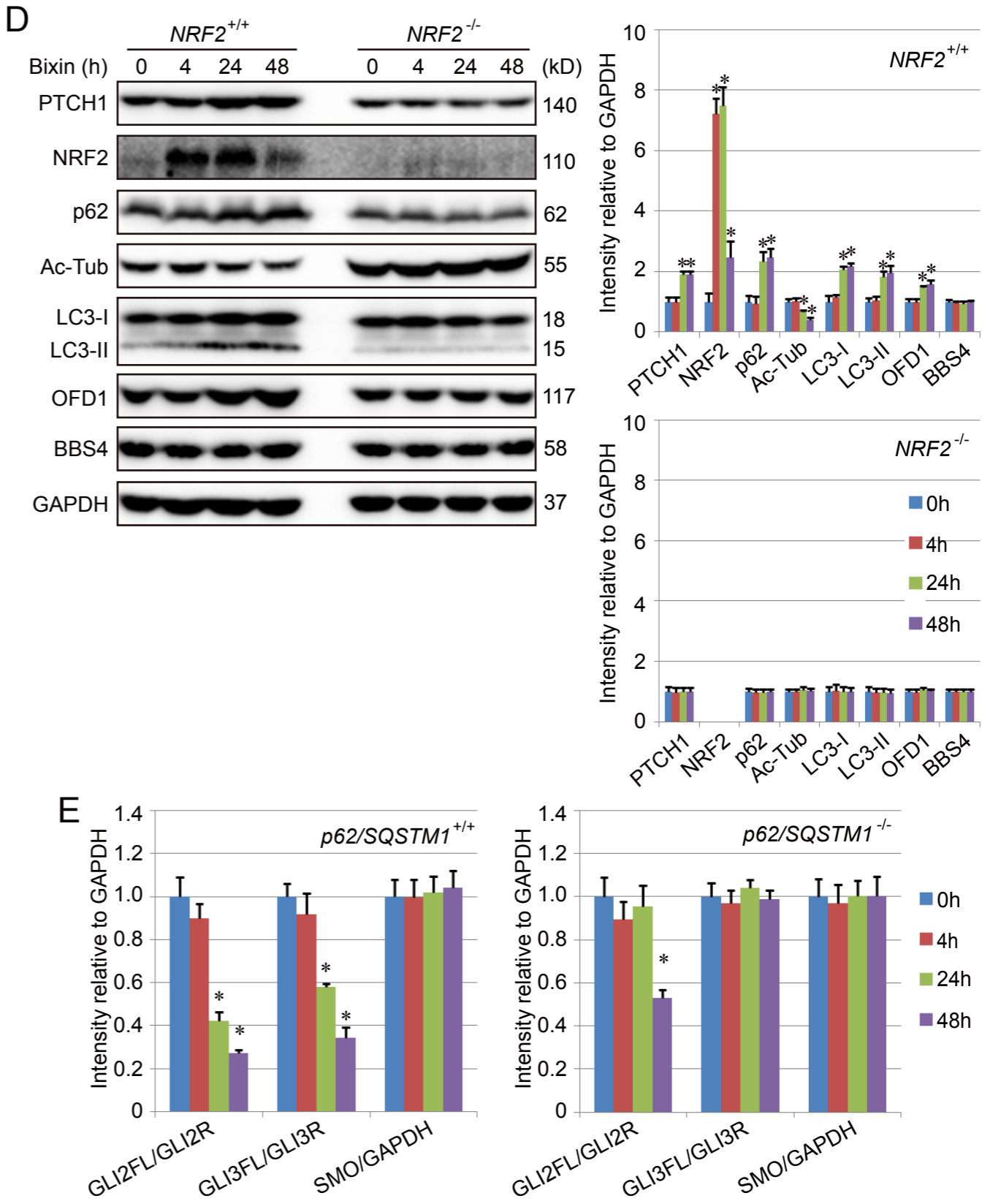

Supplement: S8 Fig — (A–C) Relative quantification of immunoblot results in Fig 5A, 5B and 5C. (D). Effect of bixin treatment in NRF2+/+ and NRF2−/− H1299 cells. (E) Relative quantification of immunoblot results in Fig 5E. Results are expressed as mean ± SD. A t test was used to compare the various groups, and p < 0.05 was considered statistically significant. *p < 0.05 compared with the control group. BBS4, Bardet–Biedl syndrome 4; NRF2, nuclear factor-erythroid 2-like 2. (PDF) [file pbio.3000620.s008.pdf]

S9 Fig

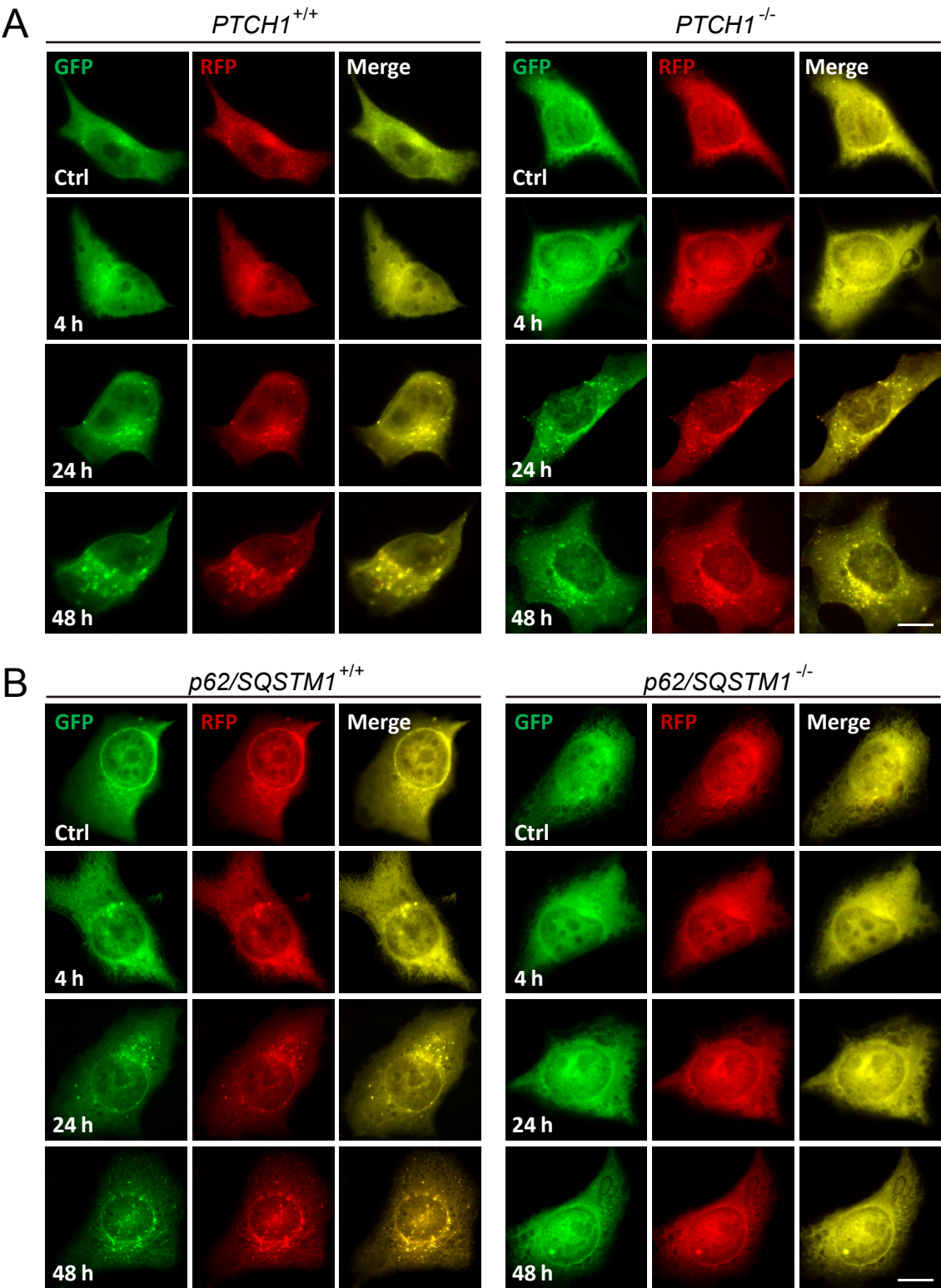

Supplement: S9 Fig — (A) PTCH1+/+ and PTCH1−/− H1299 cells were transfected with mRFP-GFP-LC3 for 24 h and then treated with bixin (40 μM) for 4, 24, and 48 h and imaged. (B) p62/SQSTM1+/+ and p62/SQSTM1−/− H1299 cells were transfected with mRFP-GFP-LC3 for 24 h and then treated with bixin (40 μM) for 4, 24, and 48 h and imaged. Yellow puncta = LC3-positive autophagosomes/inclusion bodies. (Scale bar = 5 μm.) GFP, green fluorescent protein; LC3, microtubule-associated proteins 1A/1B light chain 3B; mRFP, monomeric red fluorescent protein; PTCH1, Patched 1; SQSTM1, sequestosome 1. (PDF) [file pbio.3000620.s009.pdf]

S10 Fig

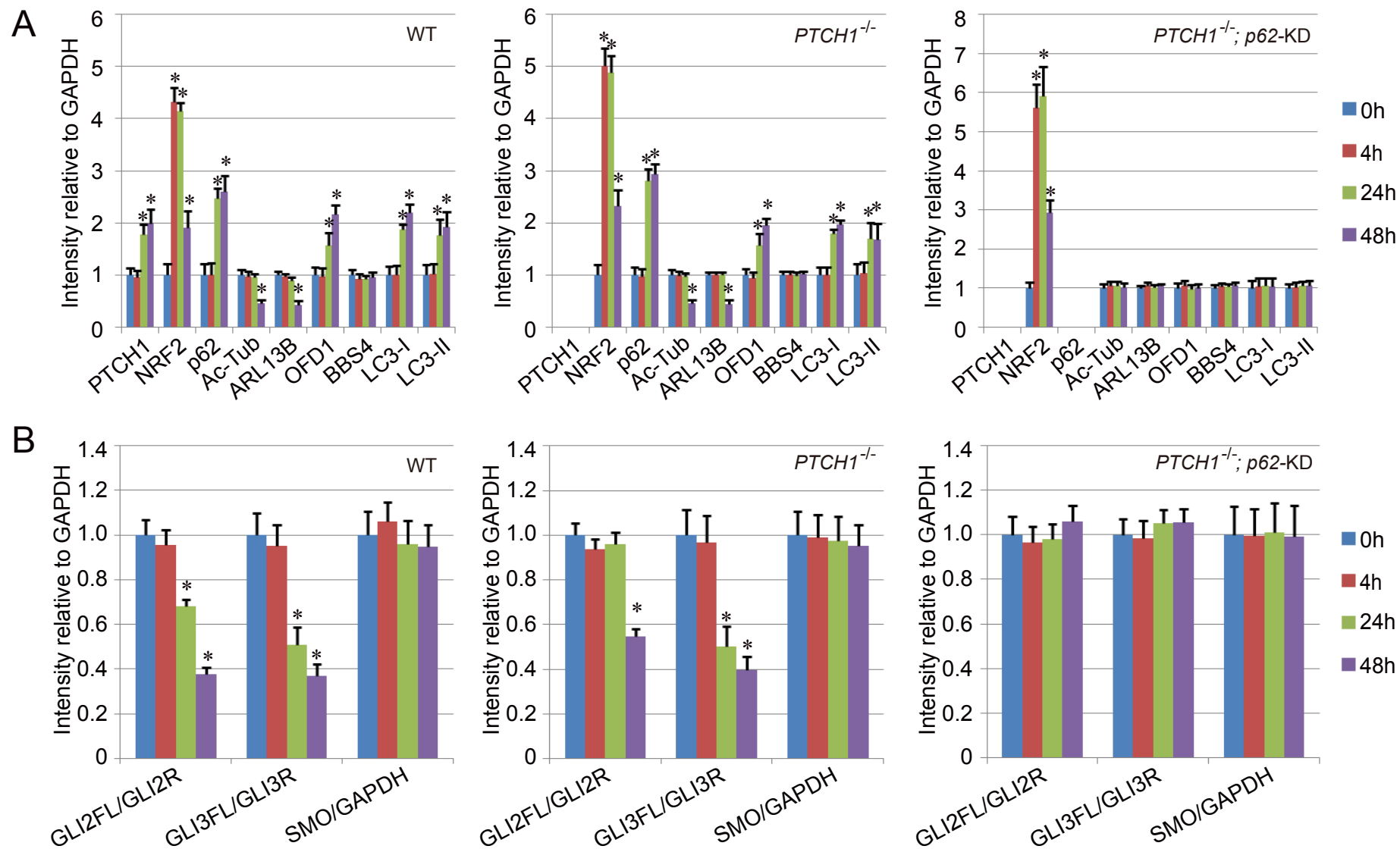

Supplement: S10 Fig — (A–B) Relative quantification of immunoblot results in Fig 6A and 6B. Results are expressed as mean ± SD. A t test was used to compare the various groups, and p < 0.05 was considered statistically significant. *p < 0.05 compared with the control group. Hh, hedgehog; KD, knockdown; NRF2, nuclear factor-erythroid 2-like 2; PTCH1, Patched 1. (PDF) [file pbio.3000620.s010.pdf]

S11 Fig

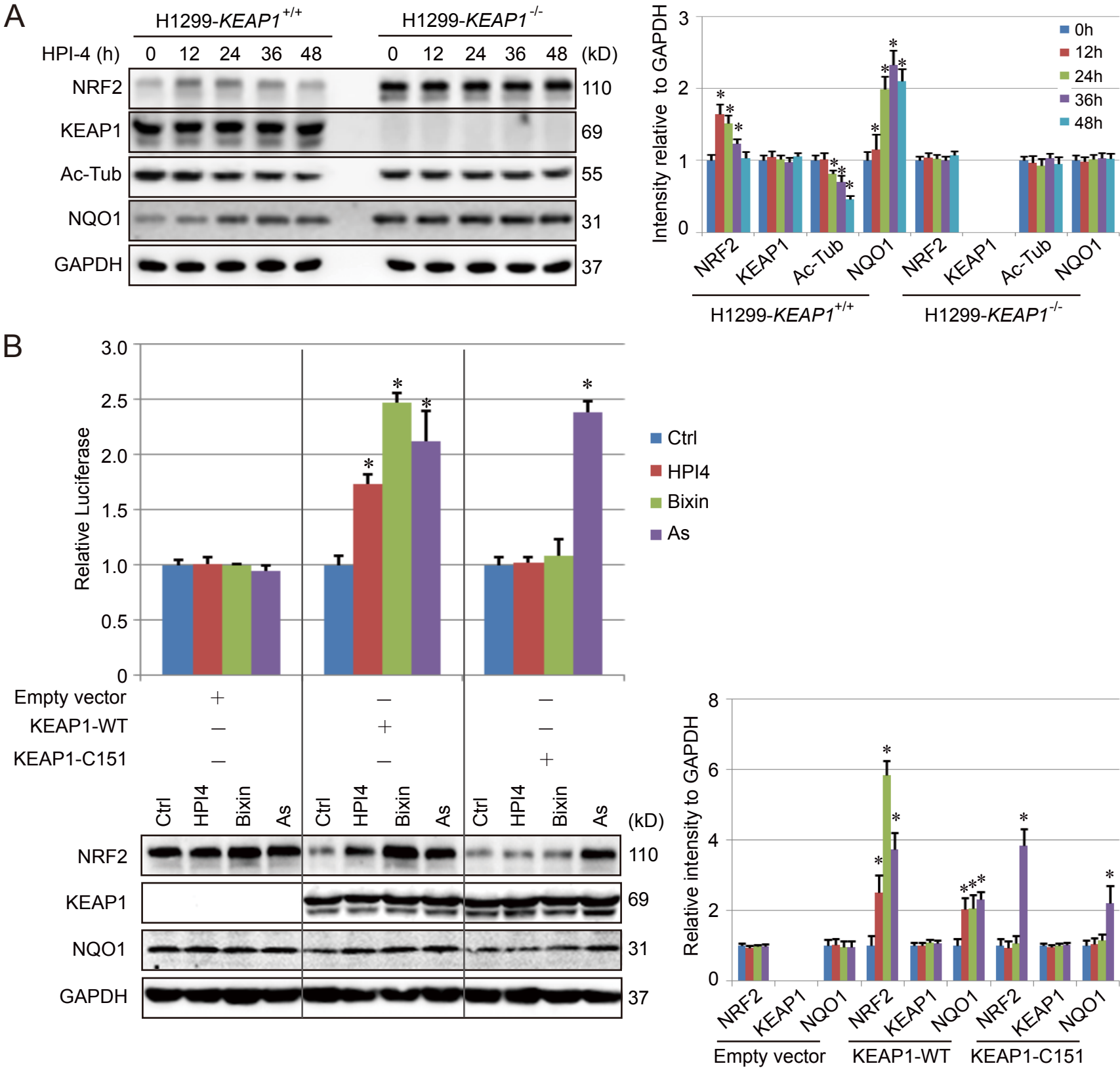

Supplement: S11 Fig — (A) Immunoblot analysis of the effect of HPI-4 treatment on H1299 KEAP1+/+ and H1299 KEAP1−/− cells treated with HPI-4 for 0, 12, 24, 36, or 48 h. (B) An H1299 KEAP1−/− cell line was transfected with plasmids encoding mGST-ARE-luciferase and TK-Renilla luciferase, along with a plasmid for KEAP1-WT or KEAP1-C151S. Following transfection for 48 h, cells were treated with 20 μM HPI-4, 40 μM bixin, or 1 μM sodium arsenite (As) for 16 h and harvested for luciferase activity detection and immunoblot assay. Results are expressed as mean ± SD. A t test was used to compare the various groups, and p < 0.05 was considered statistically significant. *p < 0.05 compared with the control group. ARE, antioxidant response element; HPI-4, hedgehog pathway inhibitor-4; KEAP1, Kelch-like ECH-asosciated protein 1; mGST, mouse glutathione S-transferase; NRF2, nuclear factor-erythroid 2-like 2; TK, thymidine kinase; WT, wild type. (PDF) [file pbio.3000620.s011.pdf]

S12 Fig

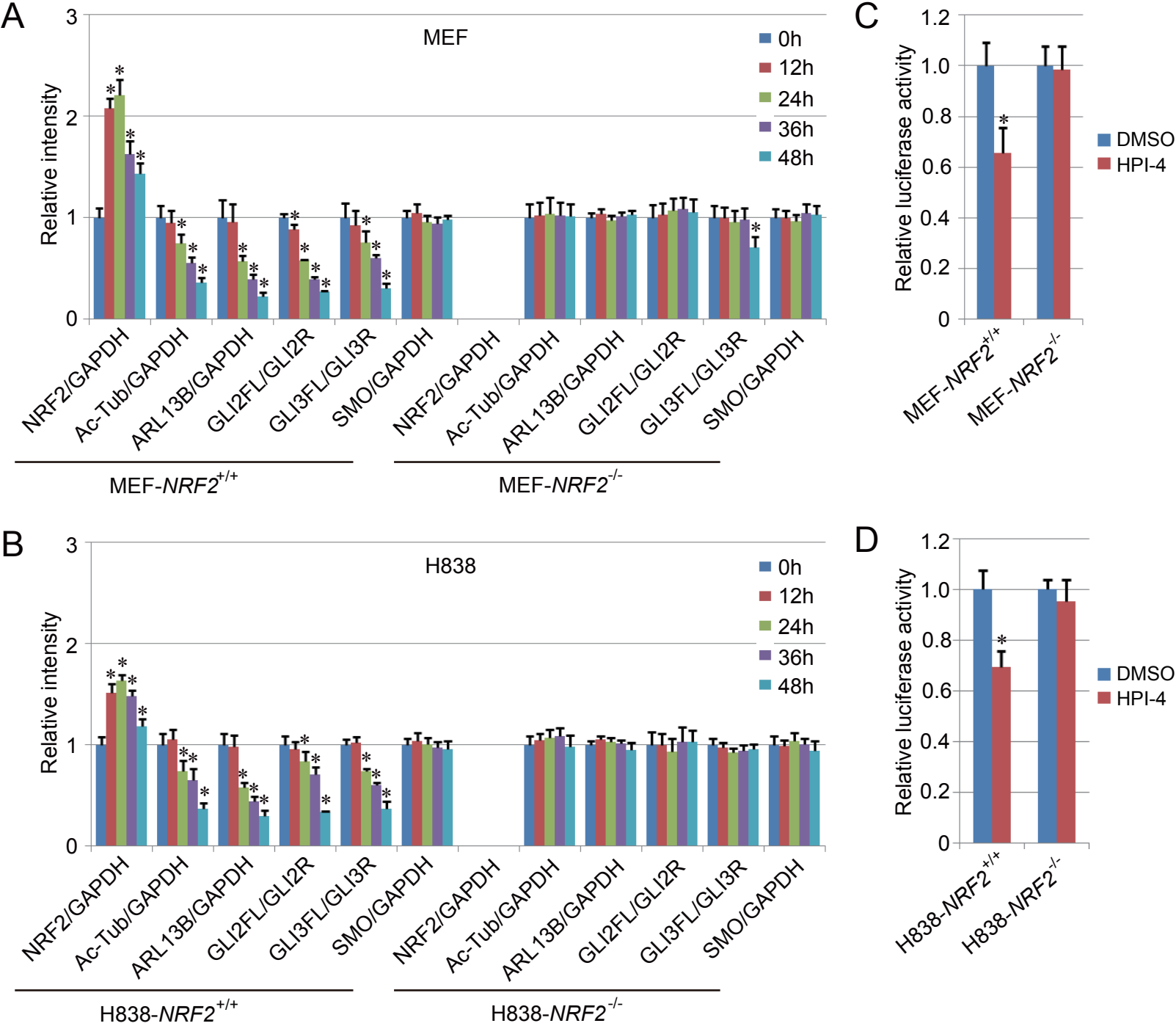

Supplement: S12 Fig — (A–B) Relative quantification of immunoblot results in Fig 7B. (C–D) GLI luciferase assay in NRF2+/+ and NRF2−/− MEF (C) and H838 (D) cells treated with HPI-4 for 48 h. Results are expressed as mean ± SD. A t test was used to compare the various groups, and p < 0.05 was considered statistically significant. *p < 0.05 compared with the control group. HPI-4, hedgehog pathway inhibitor-4; MEF, mouse embryonic fibroblast; NRF2, nuclear factor-erythroid 2-like 2. (PDF) [file pbio.3000620.s012.pdf]
