# Supplementary material for: NRF2 negatively regulates primary ciliogenesis and hedgehog signaling
Source: PLoS Biol. 2020 Feb 13;18(2):e3000620. doi: 10.1371/journal.pbio.3000620 (PMC7043785; doi:10.1371/journal.pbio.3000620)

Fig. 1B MEF-NRF2

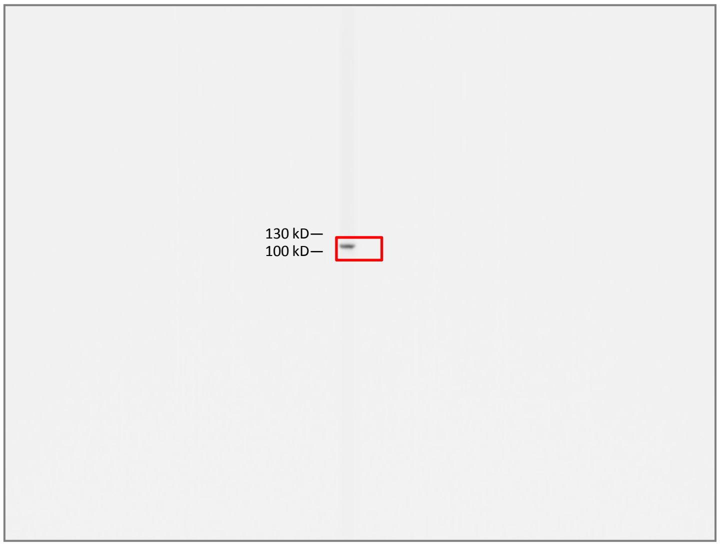

Fig. 1B MEF-Ac-Tub

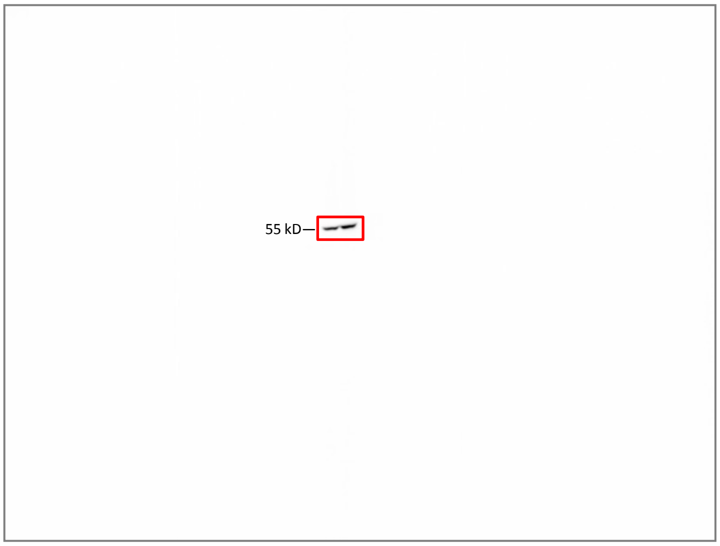

Fig. 1B MEF-ARL13B

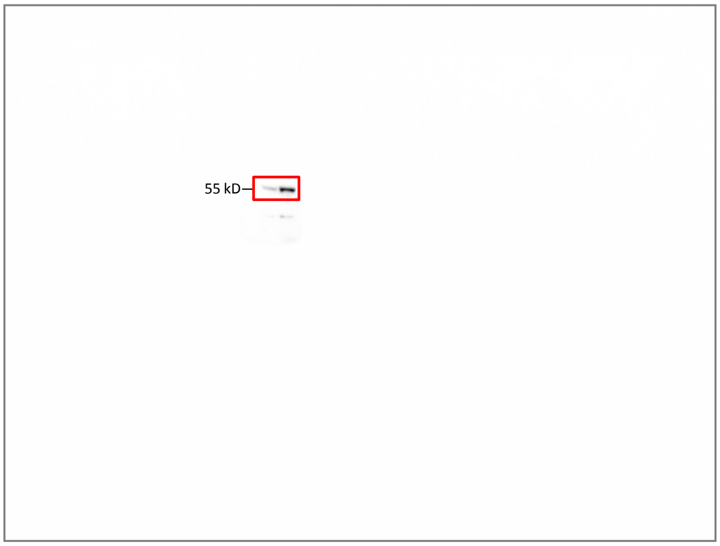

Fig. 1B BEAS-2B-NRF2

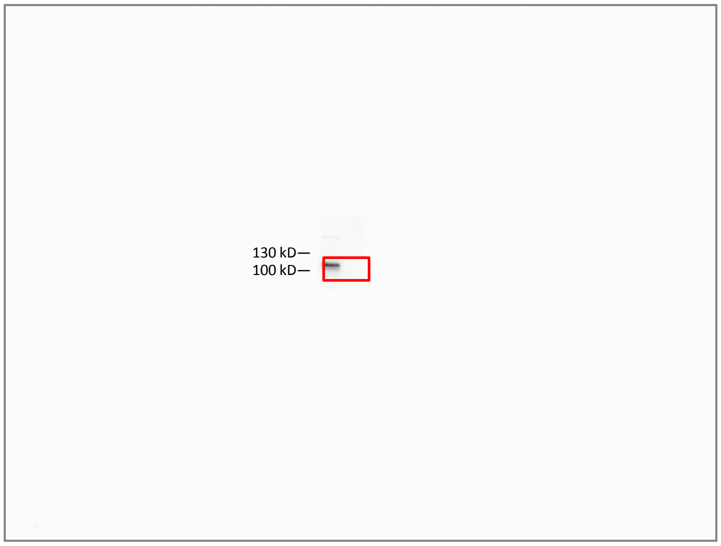

Fig. 1B BEAS-2B-Ac-Tub

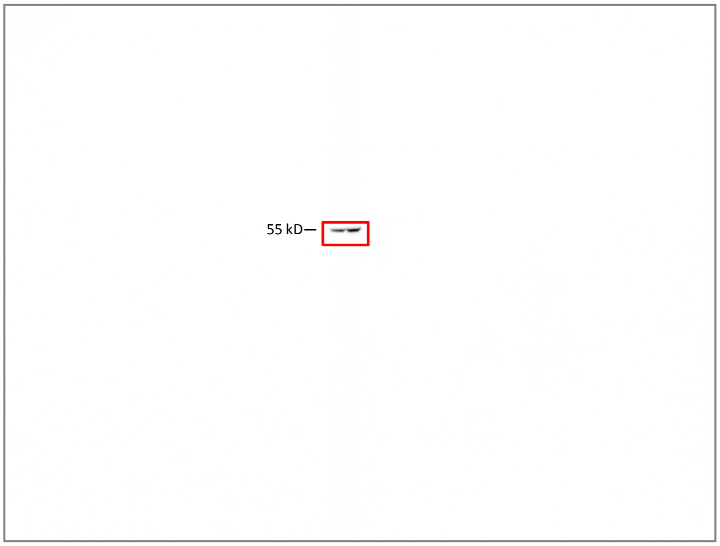

Fig. 1B BEAS-2B-ARL13B

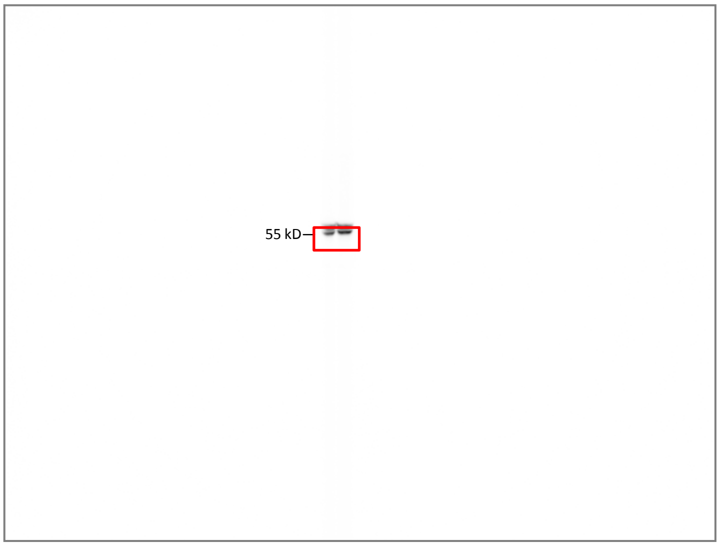

Fig. 1B H838-NRF2

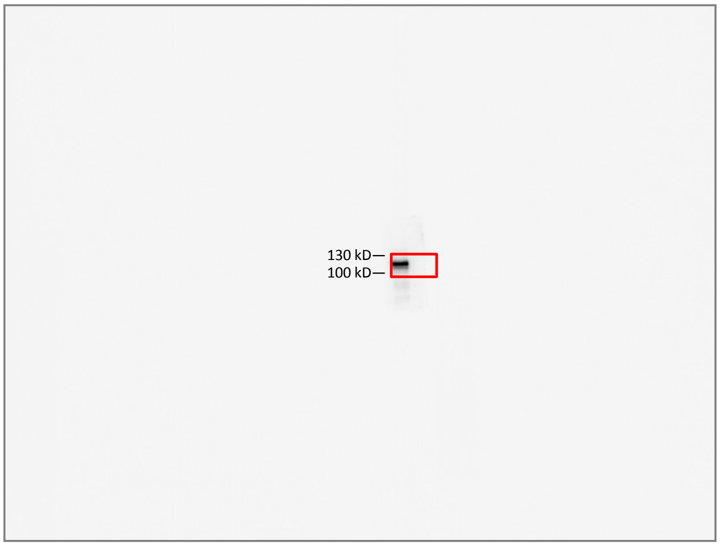

Fig. 1B H838-Ac-Tub

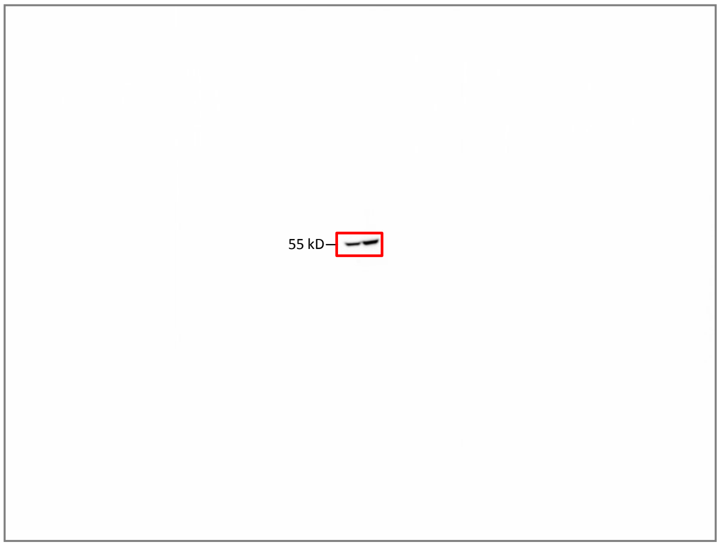

Fig. 1B H838-ARL13B

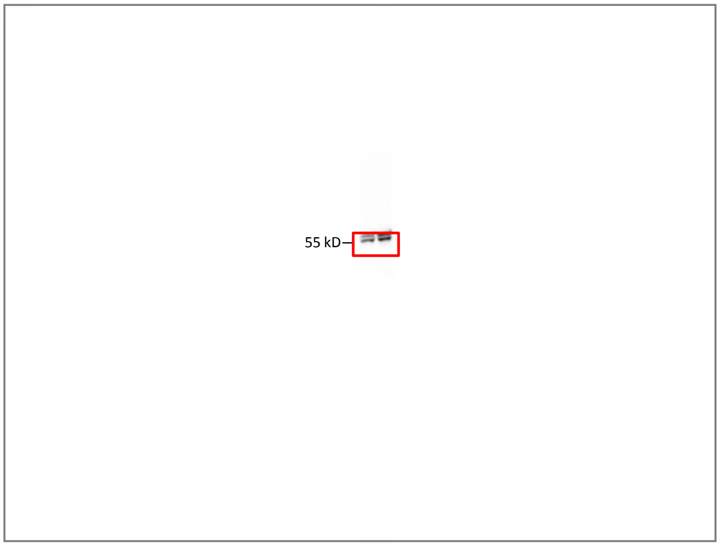

Fig. 1B MEF, BEAS-2B, H838-GAPDH

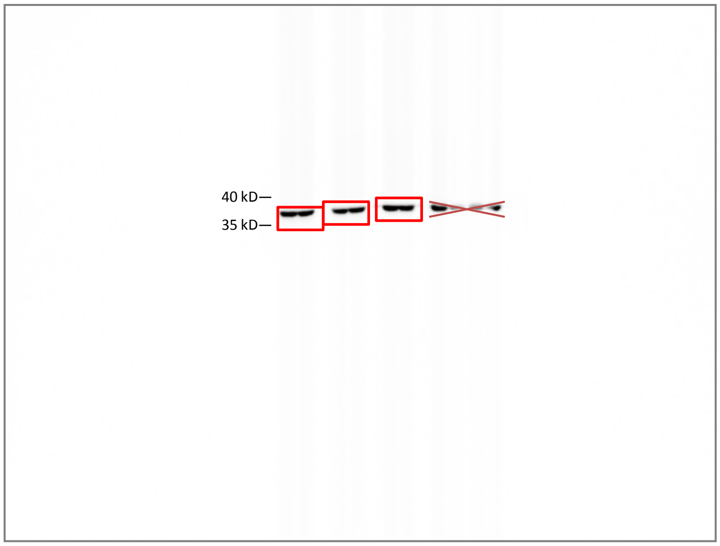

Fig. 1C MEF, BEAS-2B, H838-IFT-20

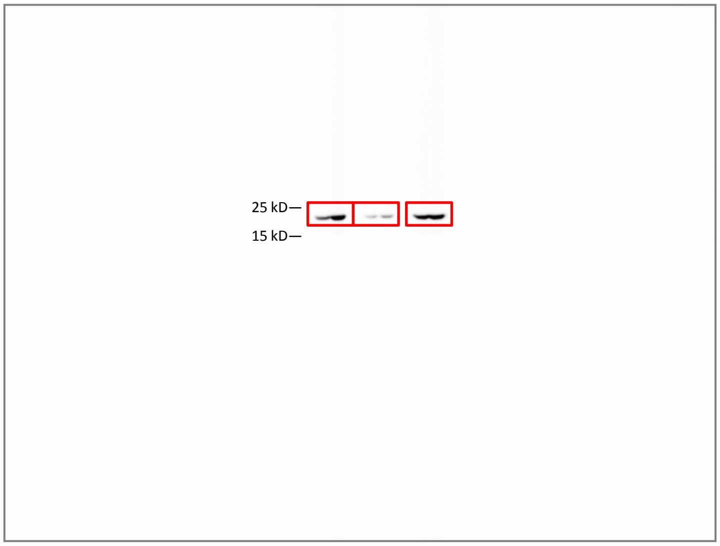

Fig. 1C MEF, BEAS-2B, H838-IFT-88

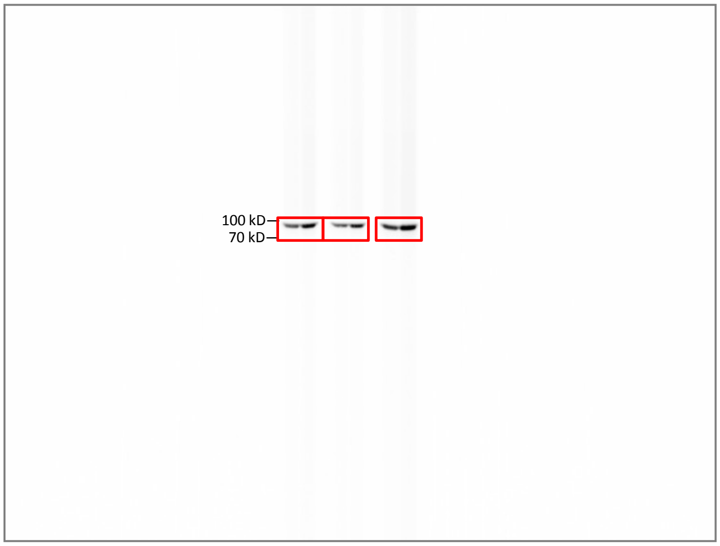

Fig. 1C MEF, BEAS-2B, H838-KIF3a

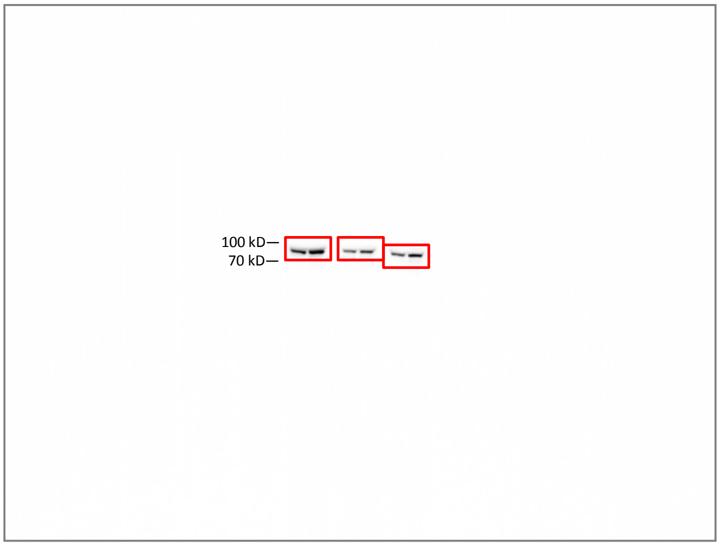

Fig. 1C MEF, BEAS-2B, H838-GAPDH

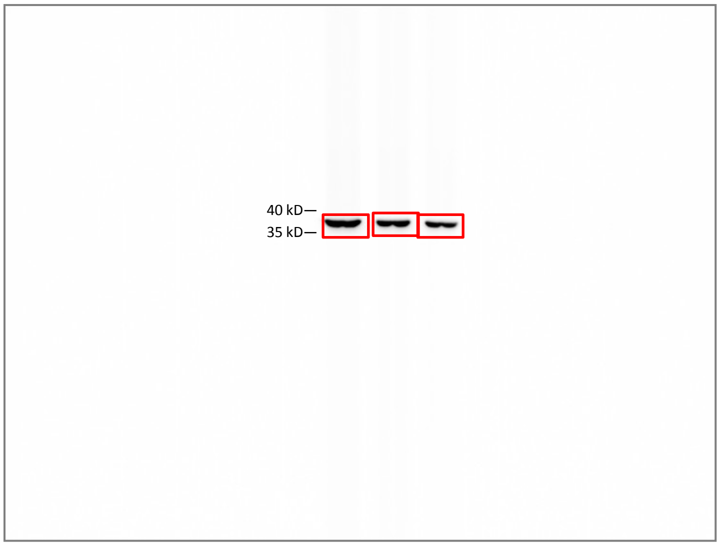

Fig. 1E MEF-GLI2

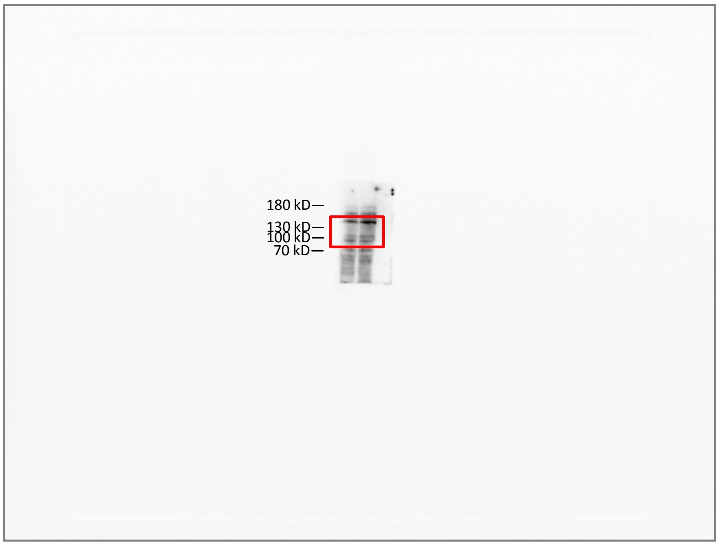

Fig. 1E MEF-GLI3

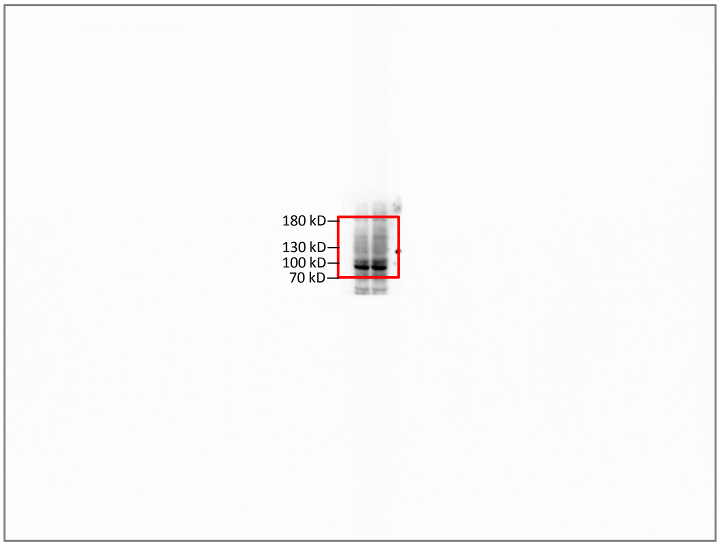

Fig. 1E BEAS-2B-GLI2

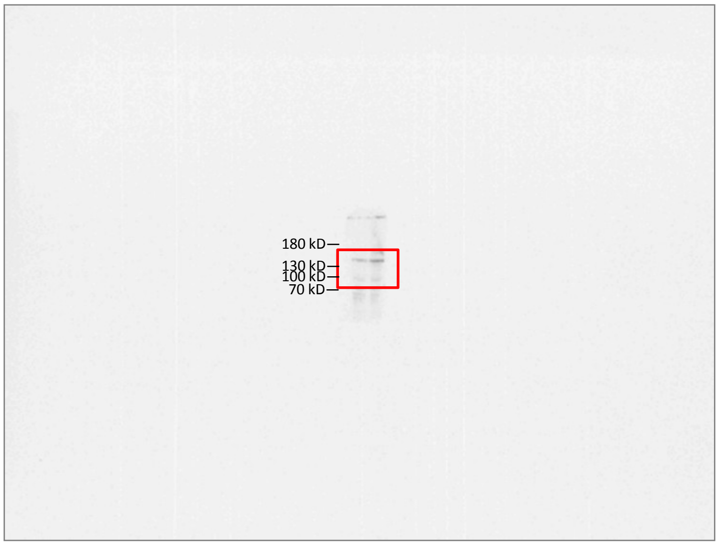

Fig. 1E BEAS-2B-GLI3

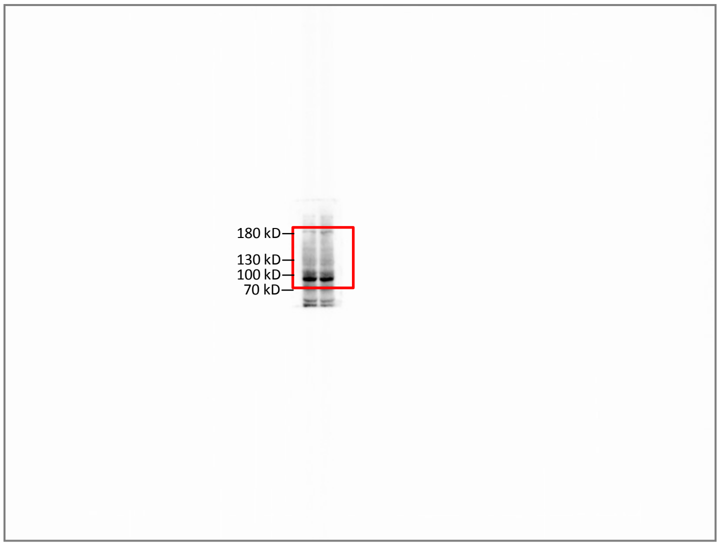

Fig. 1E H838-GLI2

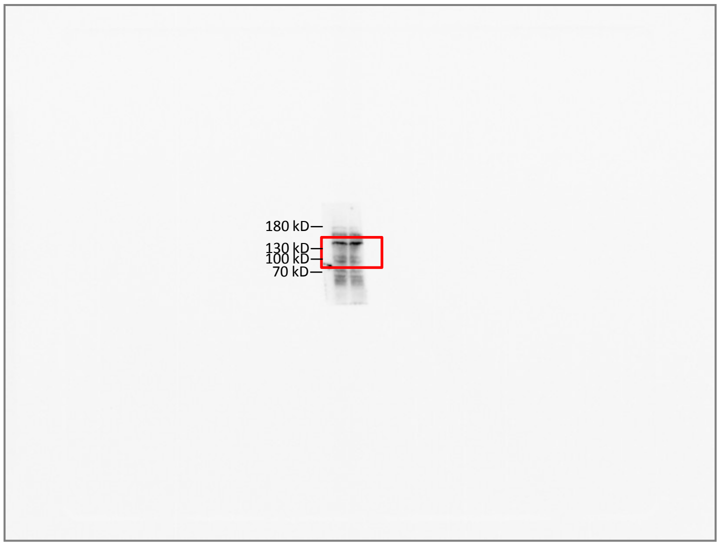

Fig. 1E H838-GLI3

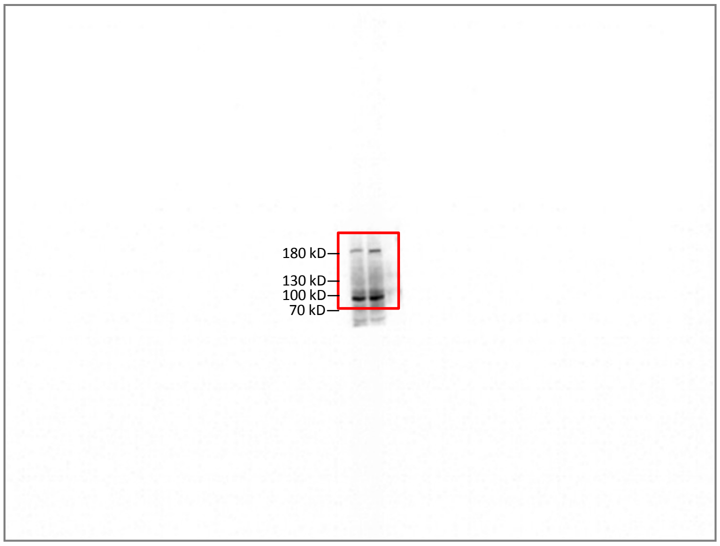

Fig. 1E MEF, BEAS-2B, H838-SMO

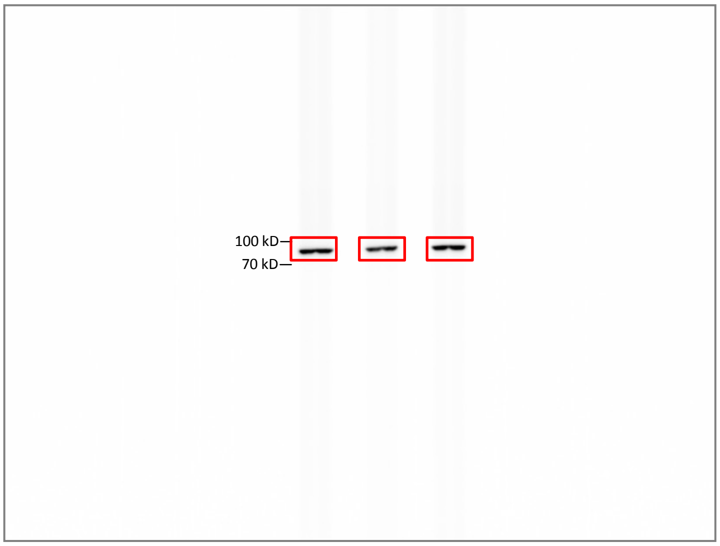

Fig. 1E MEF, BEAS-2B, H838-GAPDH

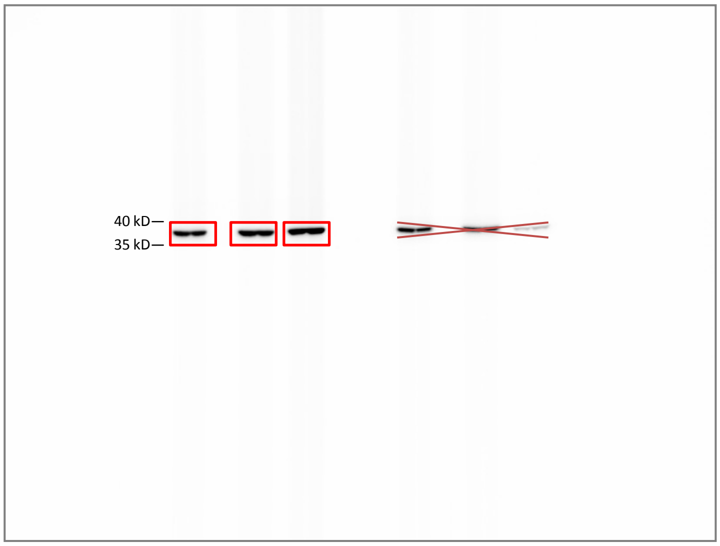

Fig. 2A NRF2

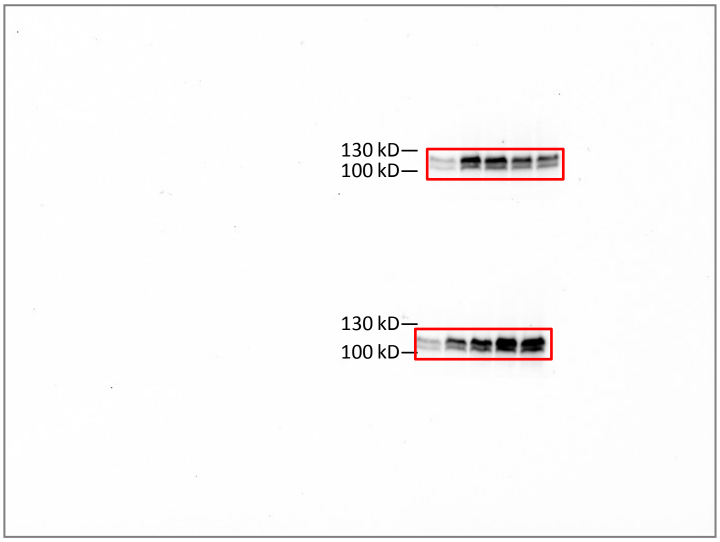

Fig. 2A KEAP1

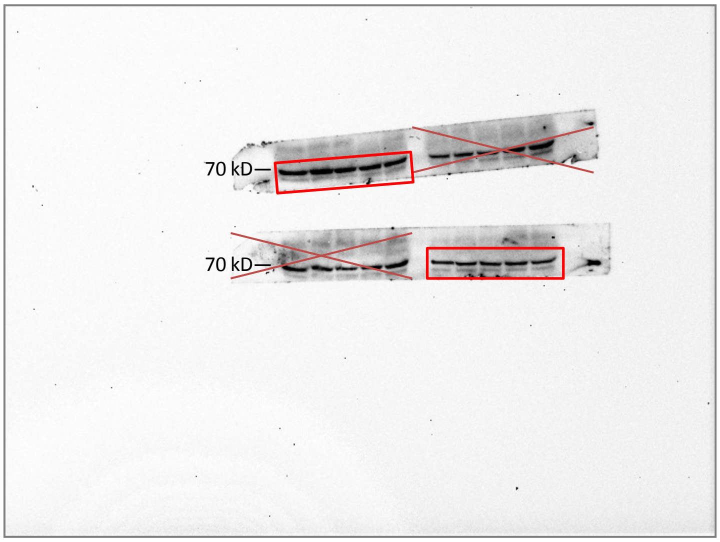

Fig. 2A NQO1

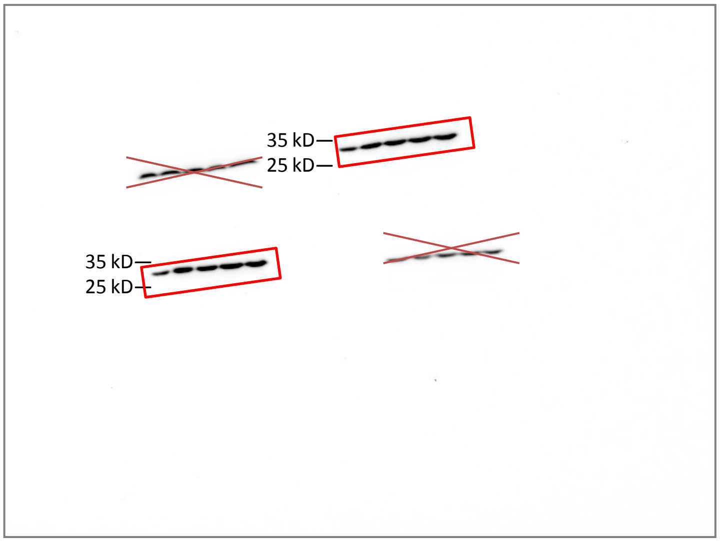

Fig. 2A Ac-Tub

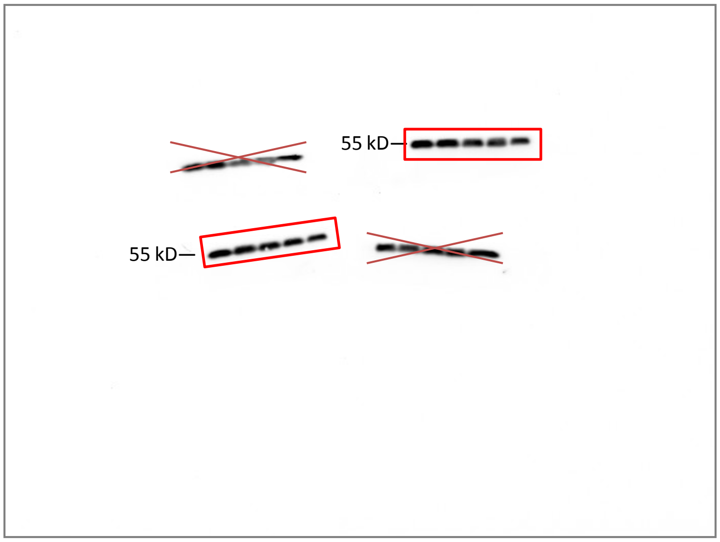

Fig. 2A Bixin-ARL13B

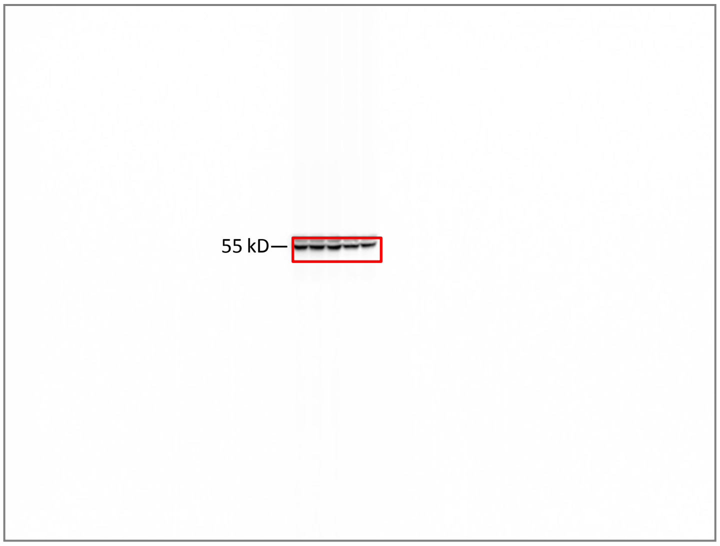

Fig. 2A pCI-NRF2-ARL13B

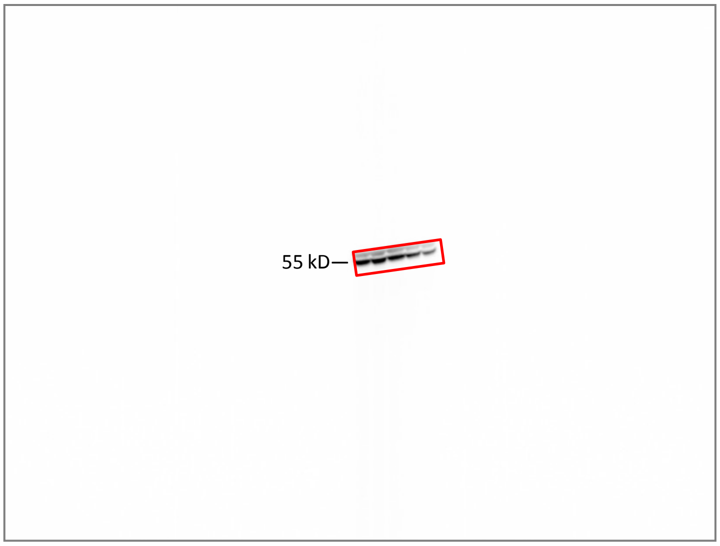

Fig. 2A Bixin-PTCH1

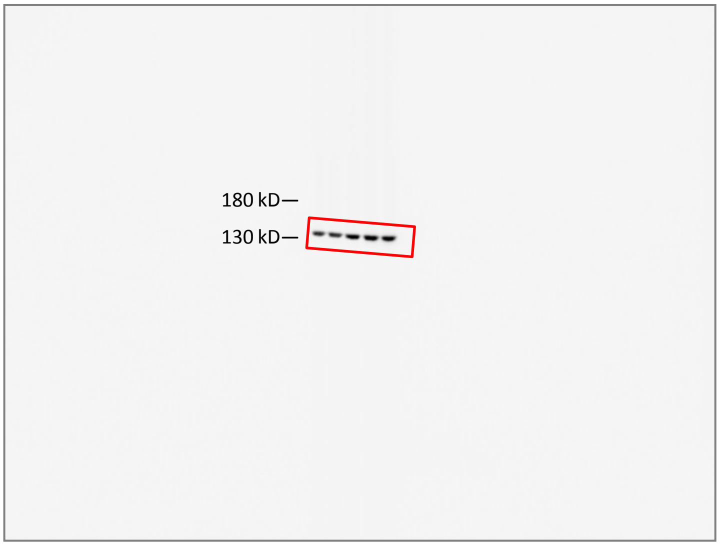

Fig. 2A pCI-NRF2-PTCH1

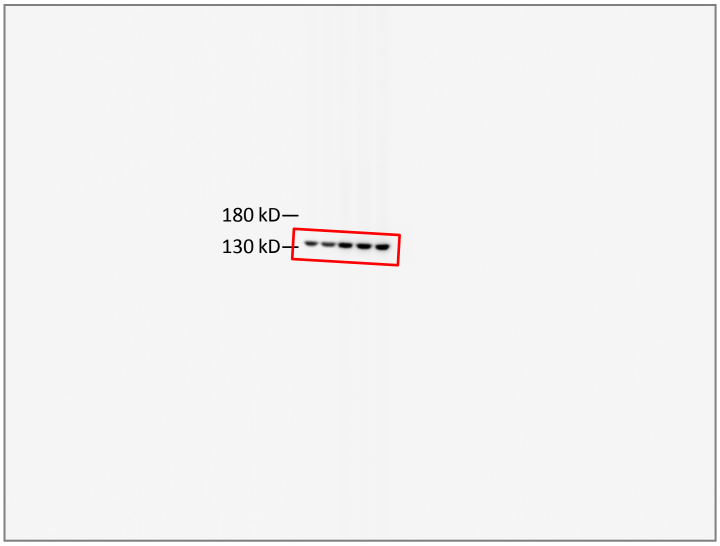

Fig. 2A GAPDH

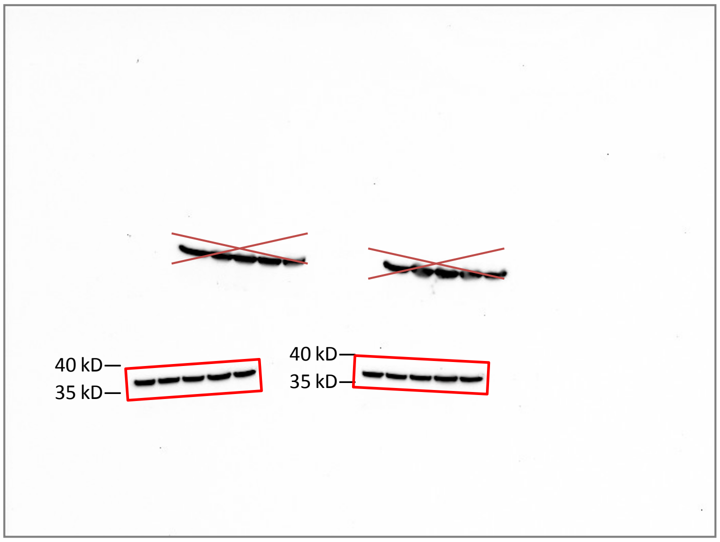

Fig. 2B Bixin-Gli2

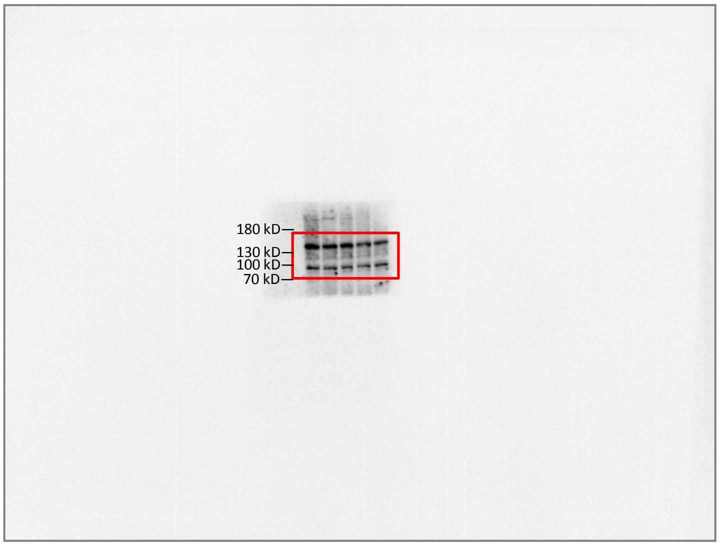

Fig. 2B Bixin-Gli3

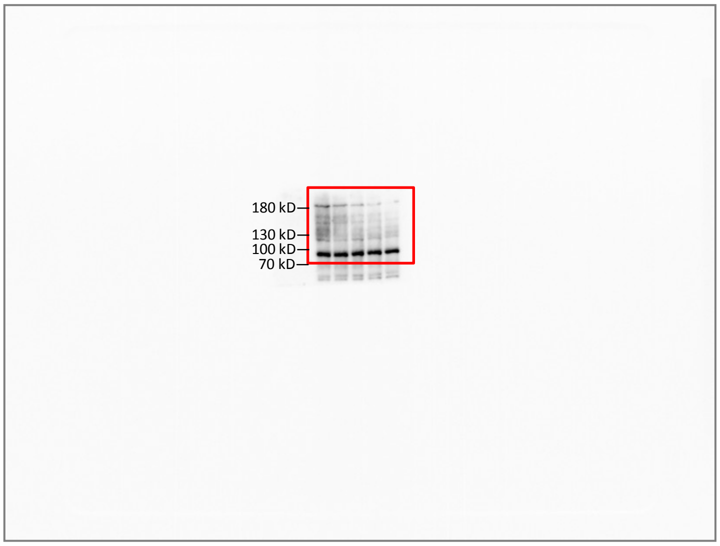

Fig. 2B pCI-NRF2-Gli2

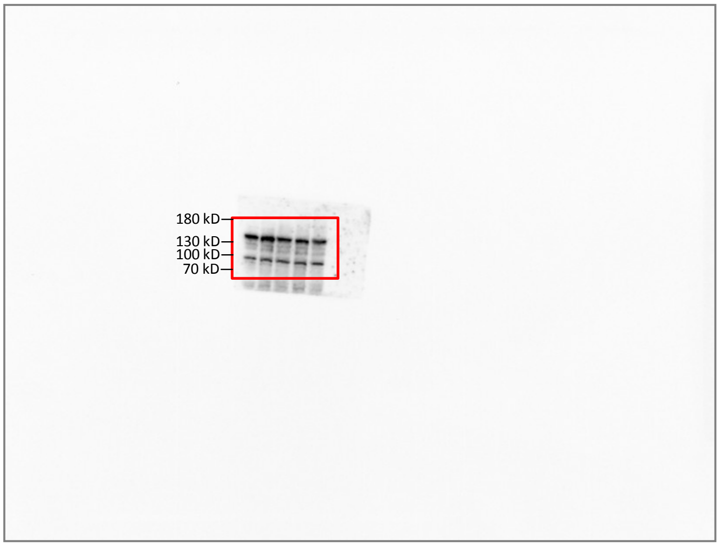

Fig. 2B pCI-NRF2-Gli3

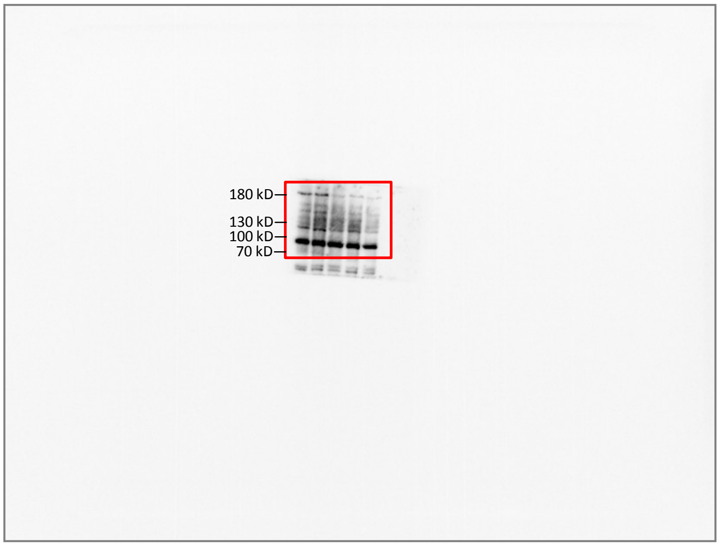

Fig. 2B SMO

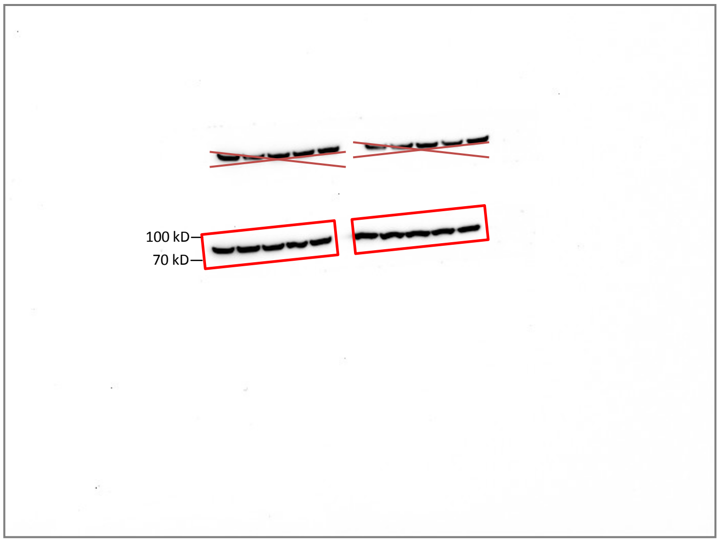

Fig. 2B GAPDH

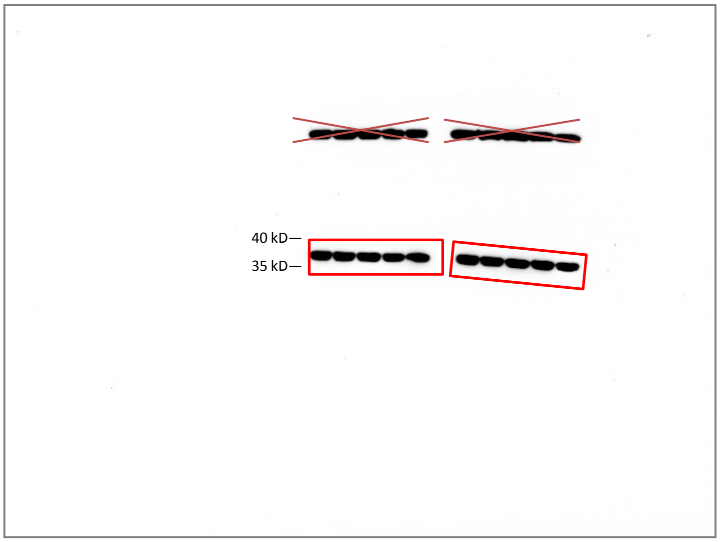

Fig. 3B Total/pull down–NRF2

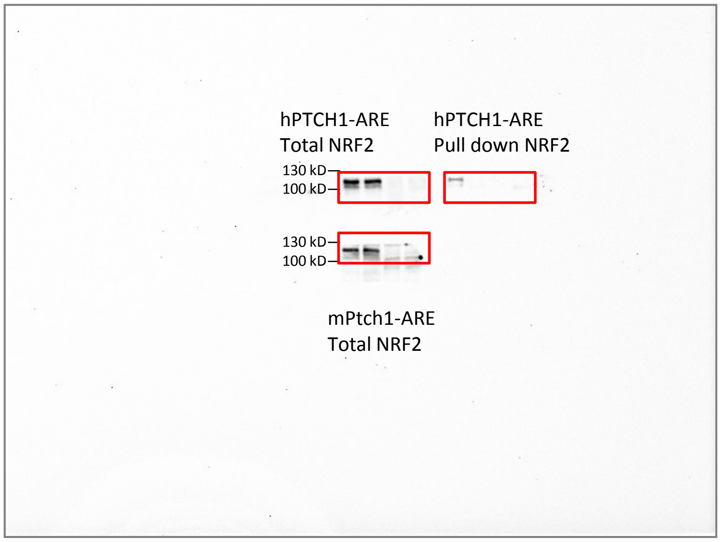

Fig. 3B Total/pull down–sMAF

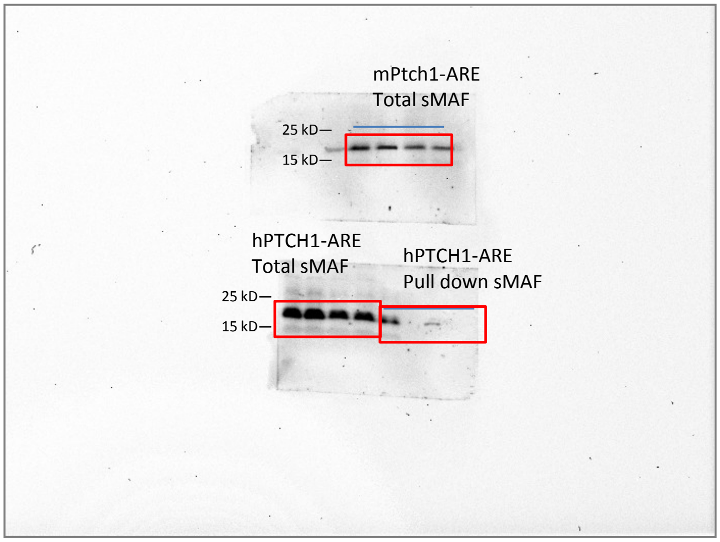

Fig. 3B pull down–NRF2

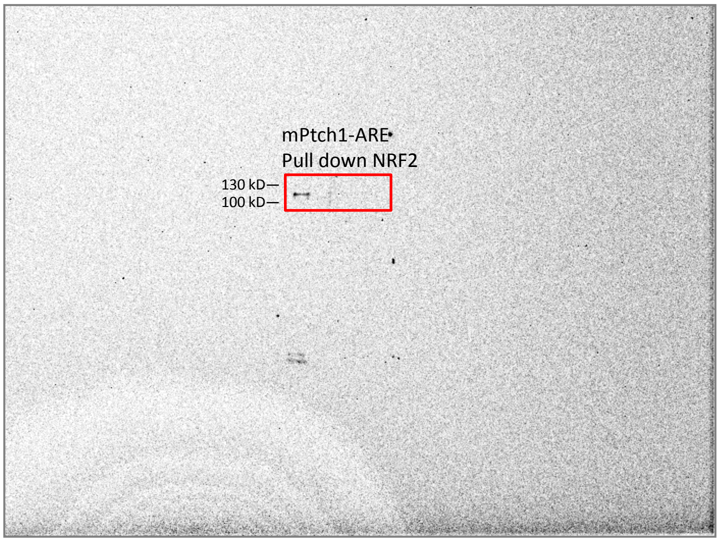

Fig. 3B pull down–sMAF

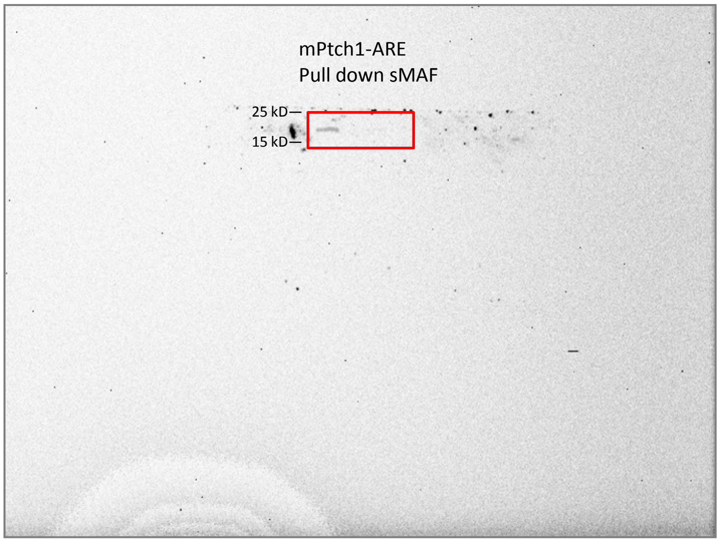

Fig. 3B GAPDH

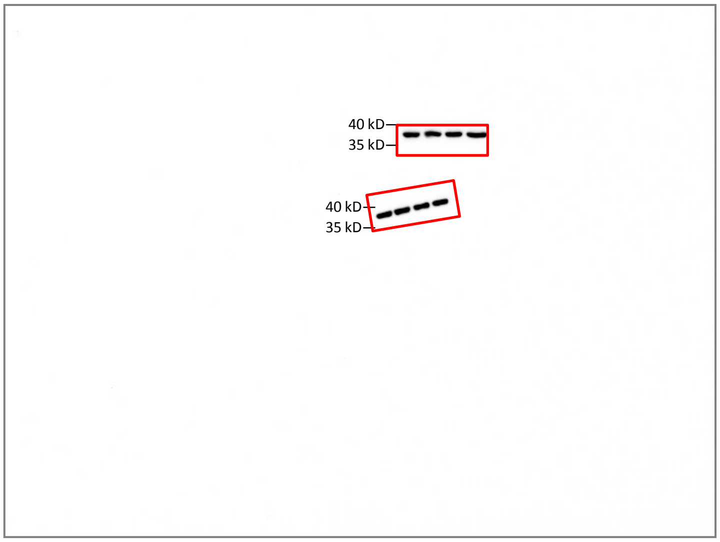

Fig. 3C MEF-PTCH1

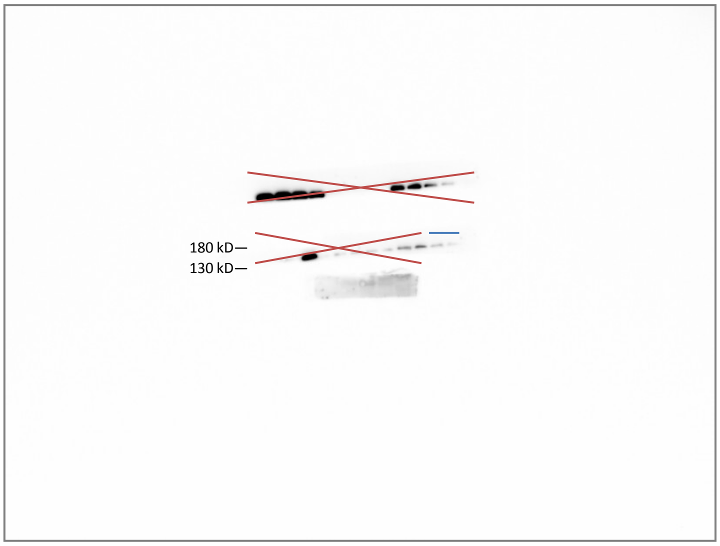

Fig. 3C H838-PTCH1

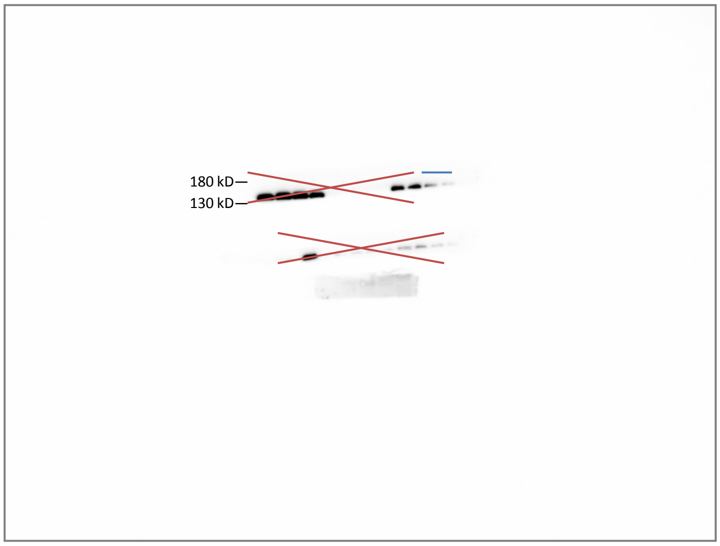

Fig. 3C MEF-GAPDH

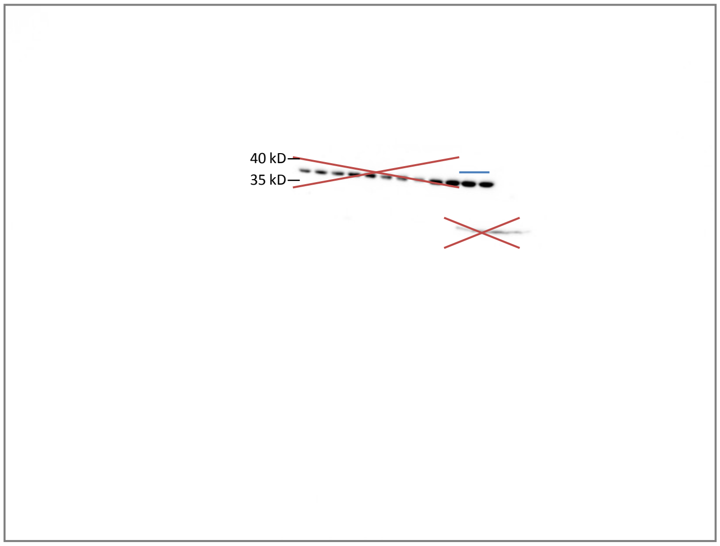

Fig. 3C H838-GAPDH

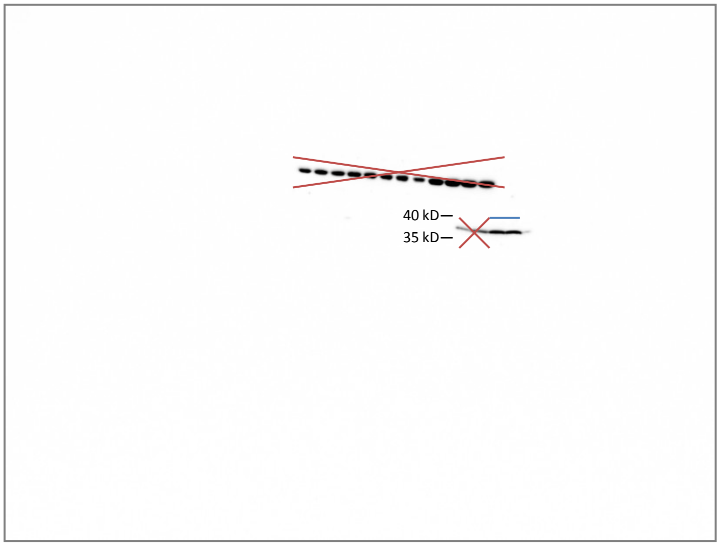

Fig. 3D NRF2

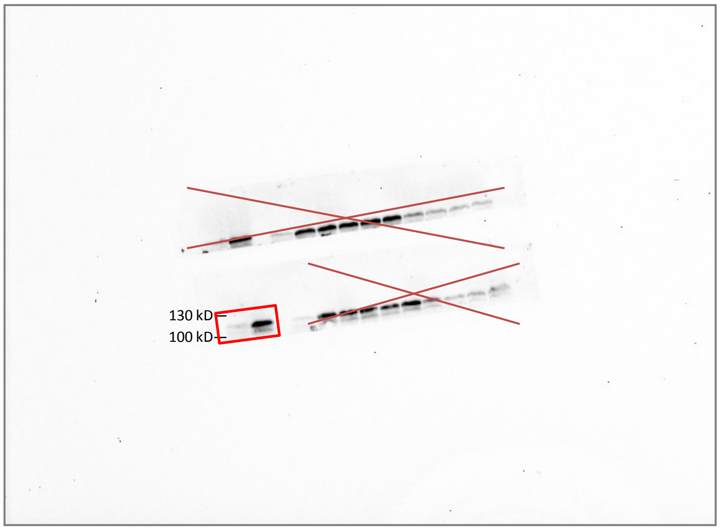

Fig. 3D PTCH1

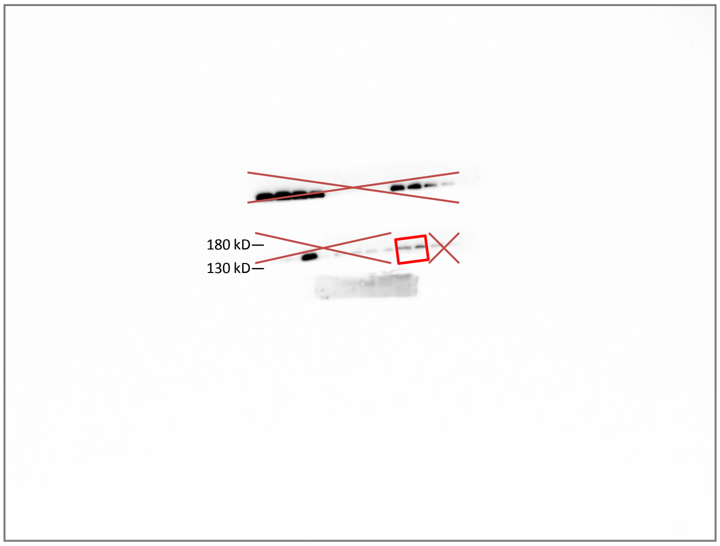

Fig. 3D KEAP1

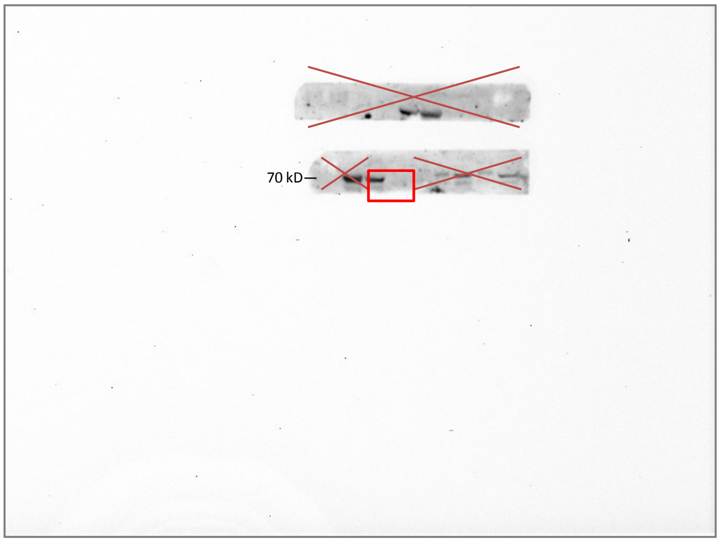

Fig. 3D NQO1

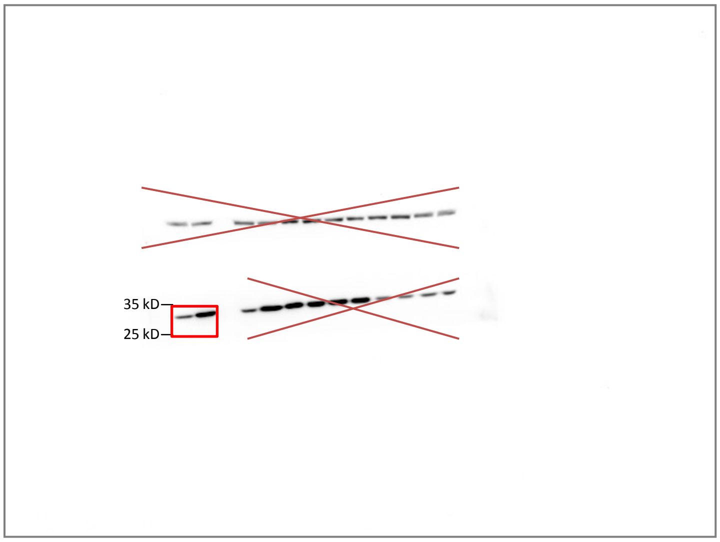

Fig. 3D GCLM

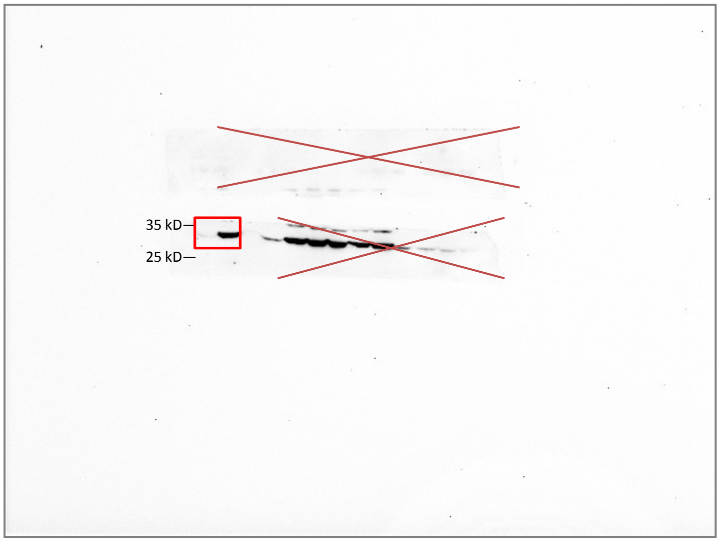

Fig. 3D Ac-Tub

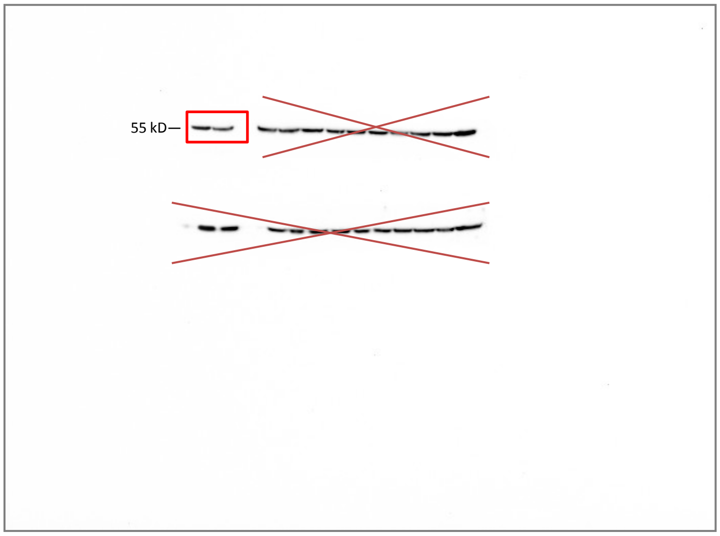

Fig. 3D ARL13B

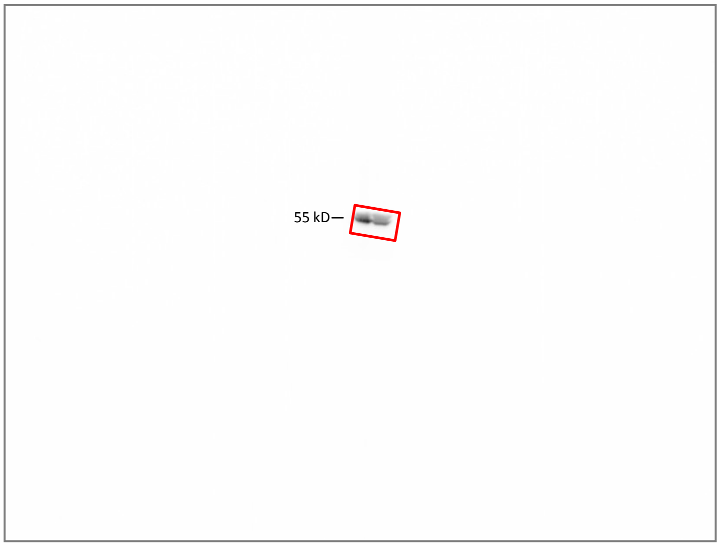

Fig. 3D GAPDH

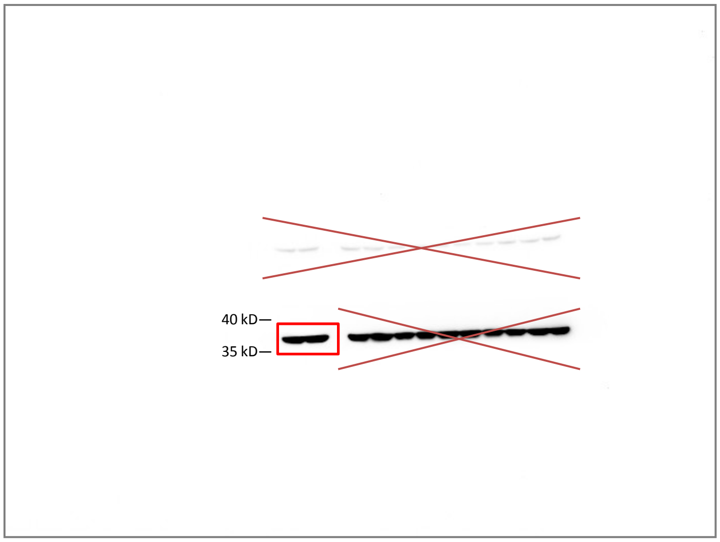

Fig. 4A PTCH1

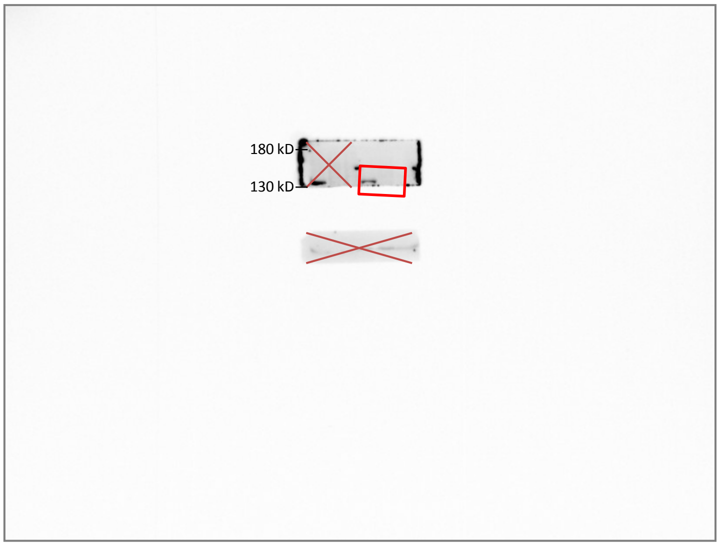

Fig. 4A GAPDH

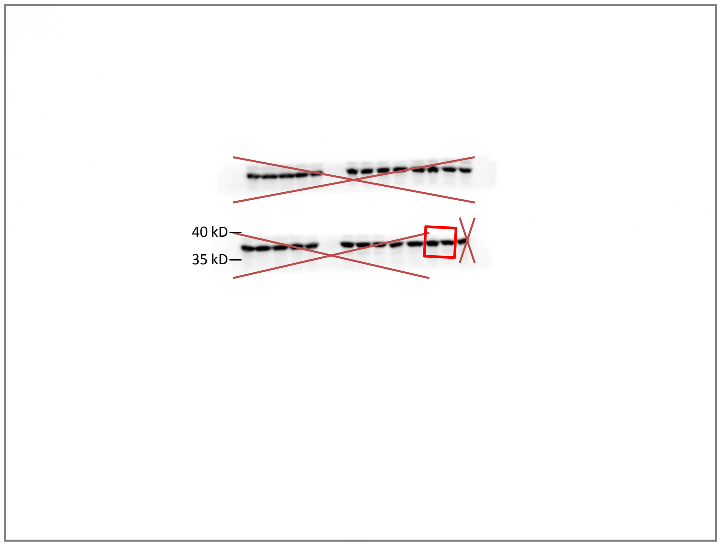

Fig. 4A GLI2

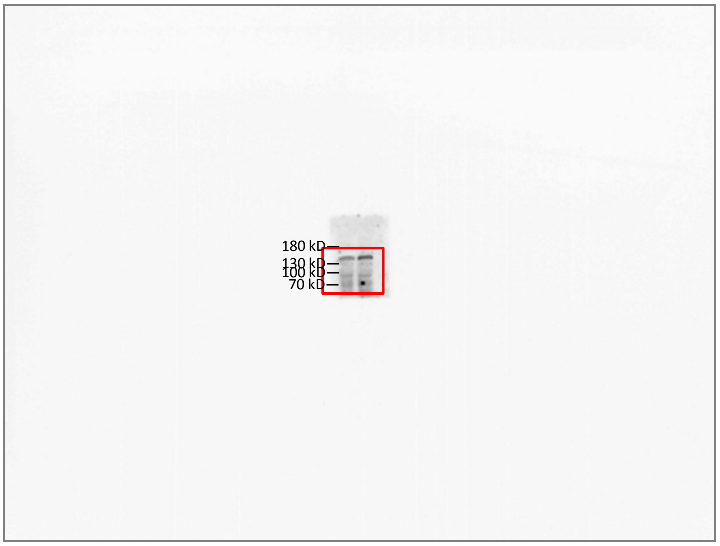

Fig. 4A GLI3

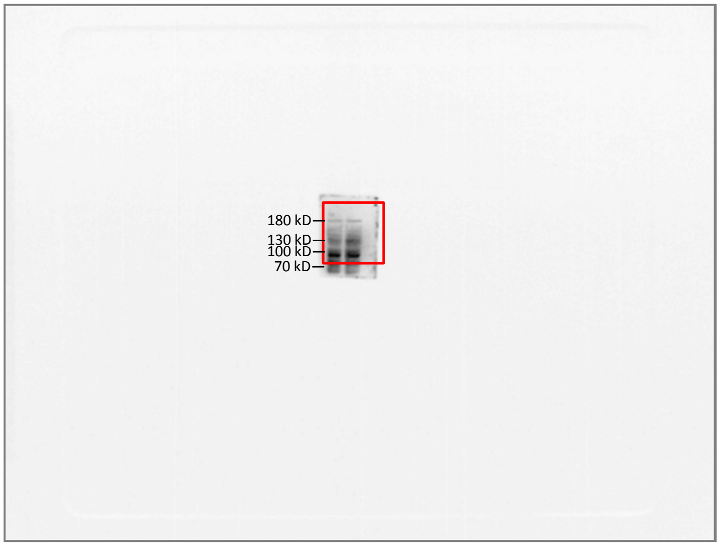

Fig. 4C NRF2

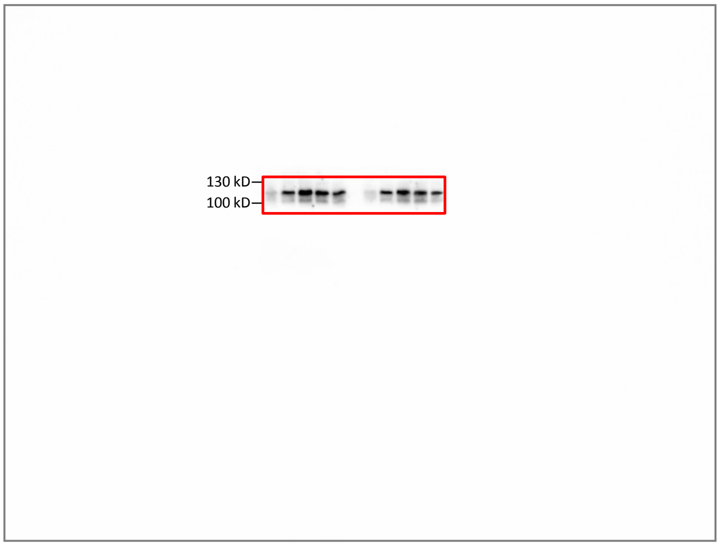

Fig. 4C PTCH1

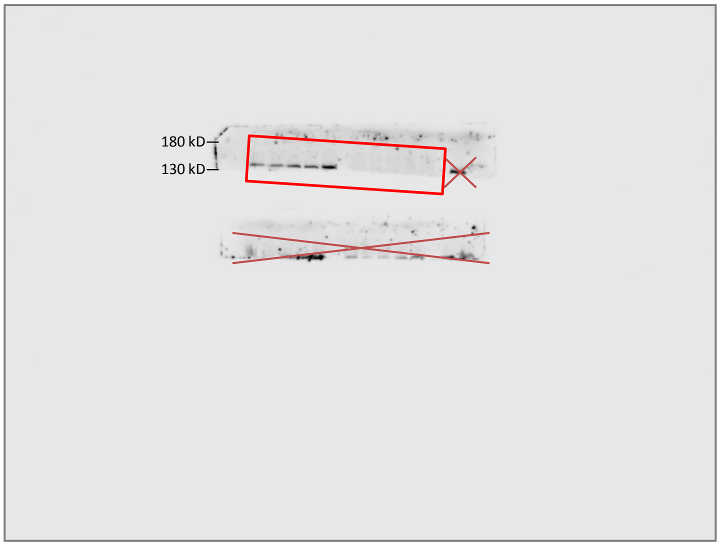

Fig. 4C Ac-Tub

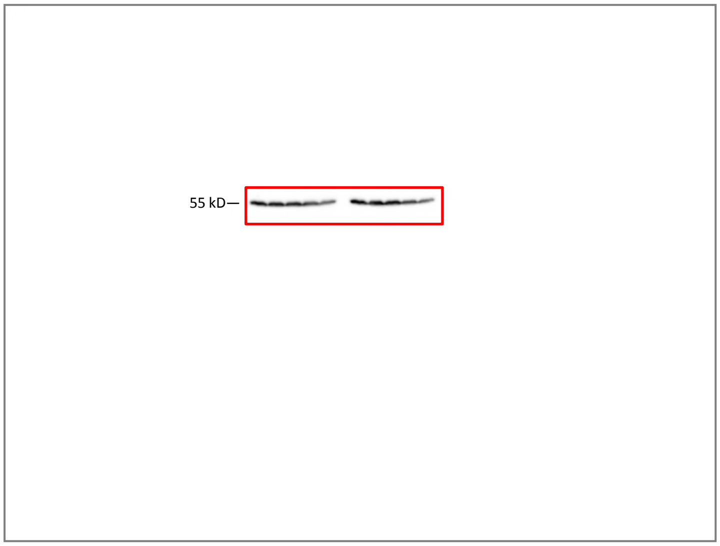

Fig. 4C ARL13B

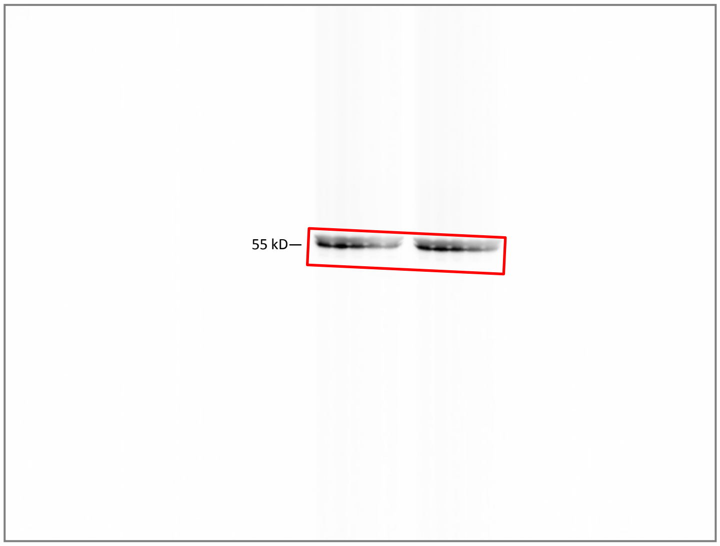

Fig. 4C NQO1

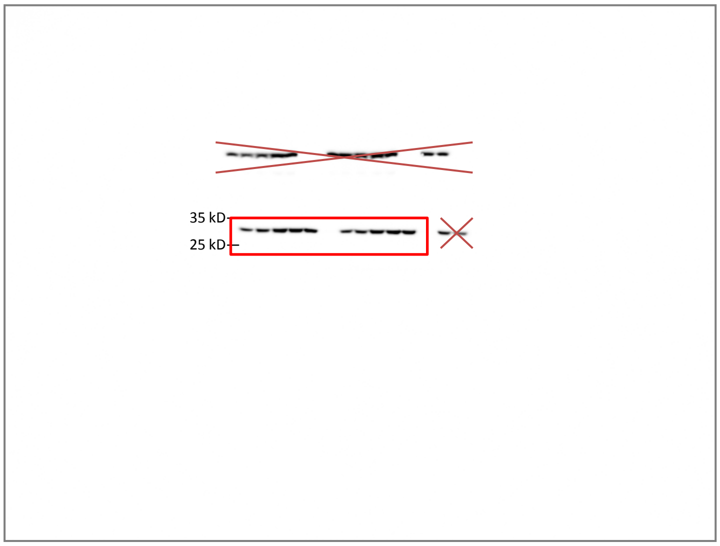

Fig. 4C GAPDH

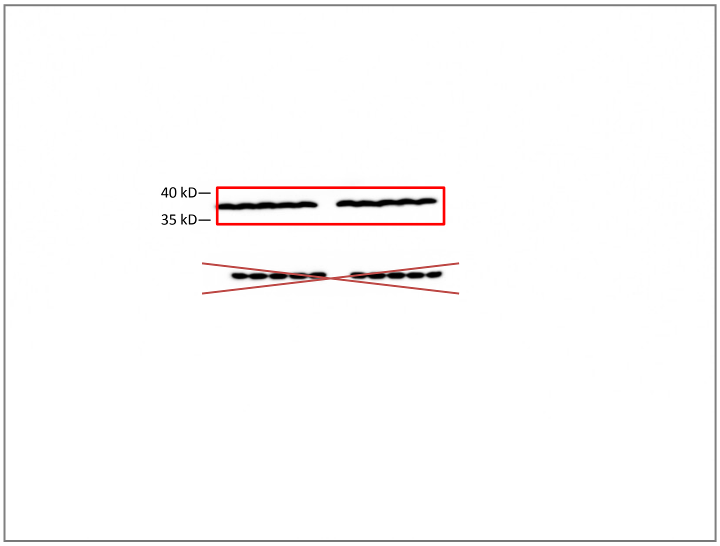

Fig. 4D $PTCH1^{+/+}$ GLI2

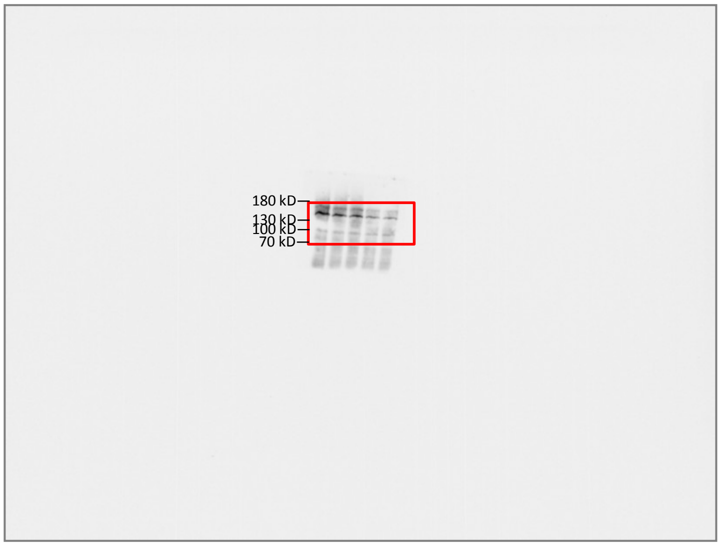

Fig. 4D $PTCH1^{-/-}$ GLI2

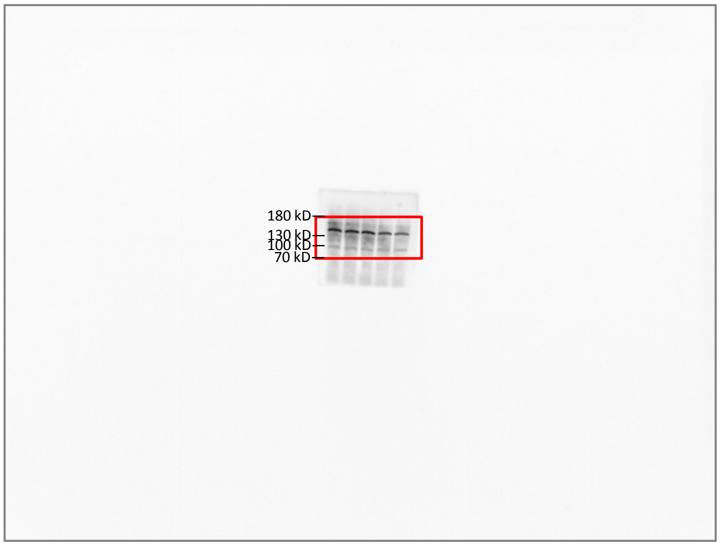

Fig. 4D $PTCH1^{+/+}$ GLI3

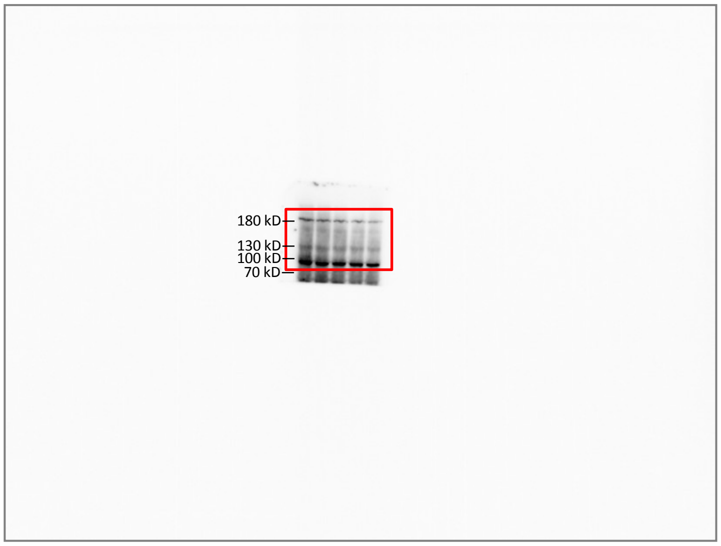

Fig. 4D $PTCH1^{-/-}$ GLI3

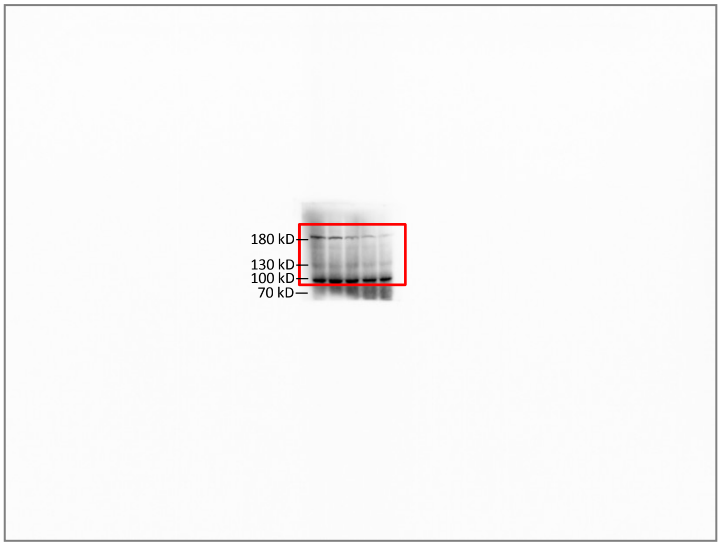

Fig. 4D $PTCH1^{+/+}$, $PTCH1^{-/-}$ SMO

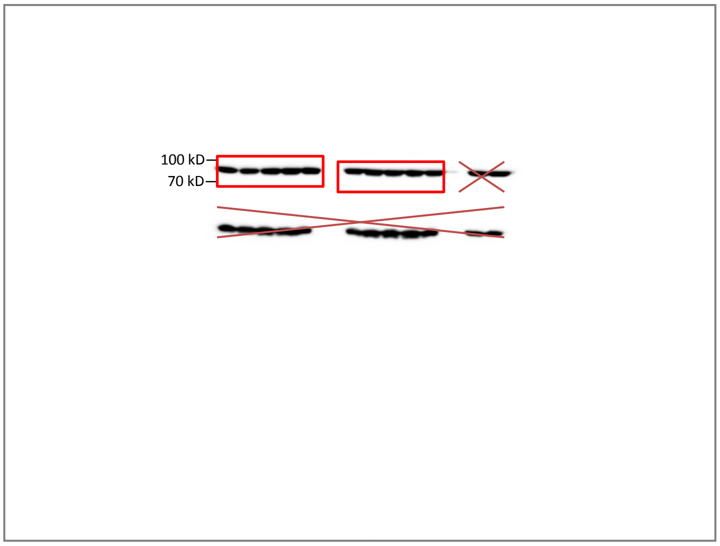

Fig. 4D $PTCH1^{+/+}$, $PTCH1^{-/-}$ GAPDH

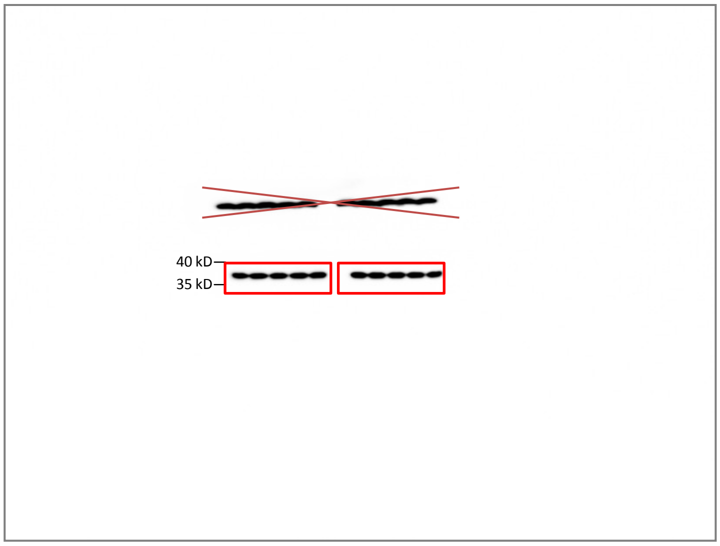

Fig. 5A NRF2

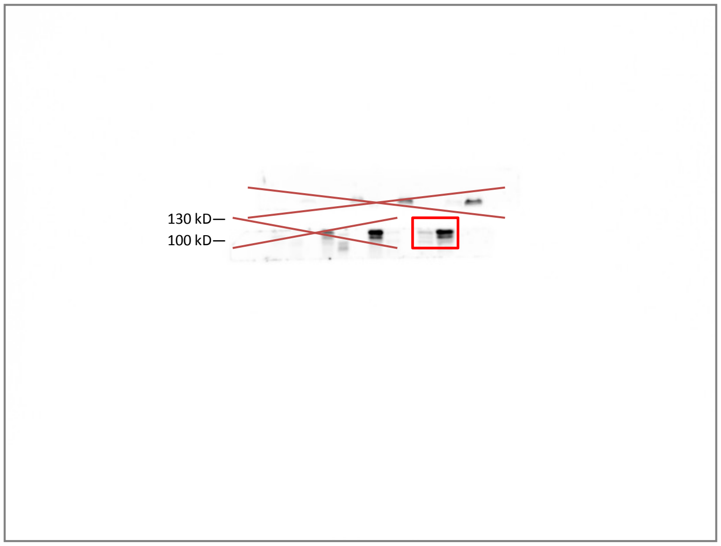

Fig. 5A p62

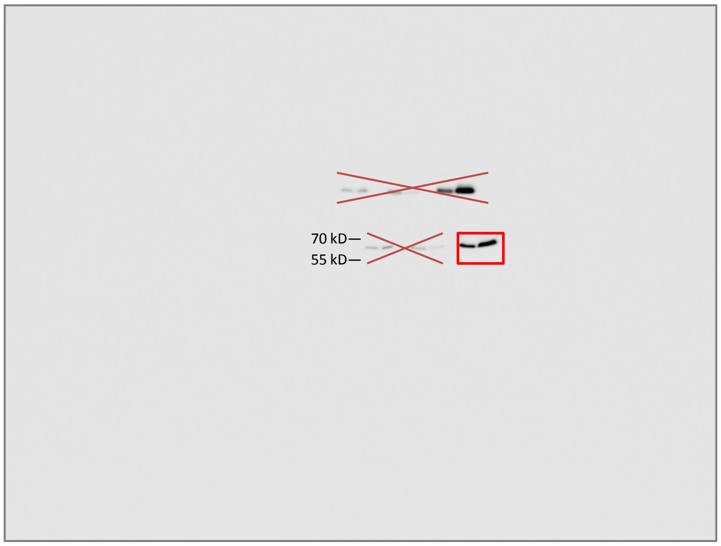

Fig. 5A LC3

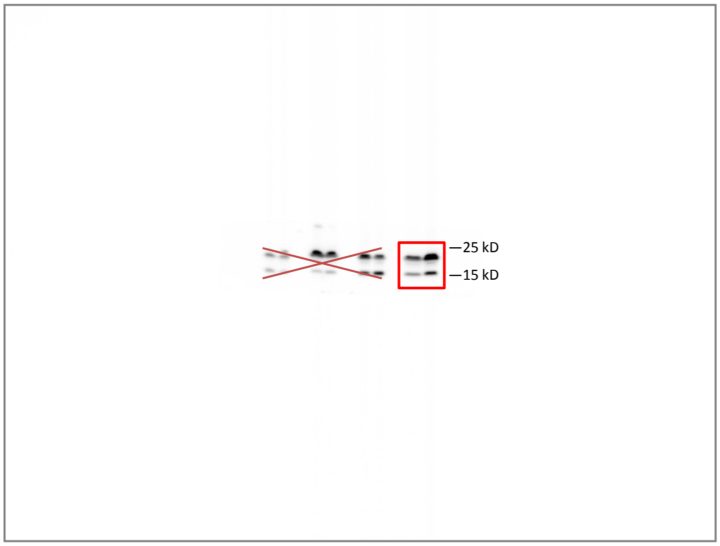

Fig. 5A OFD1

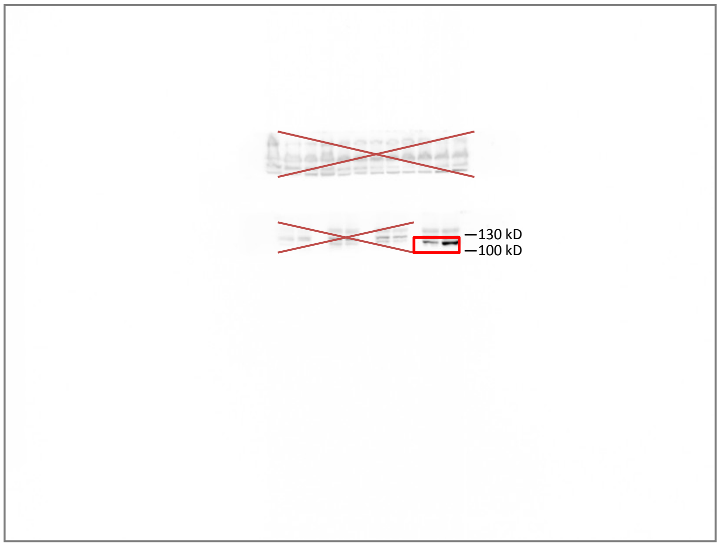

Fig. 5A BBS4

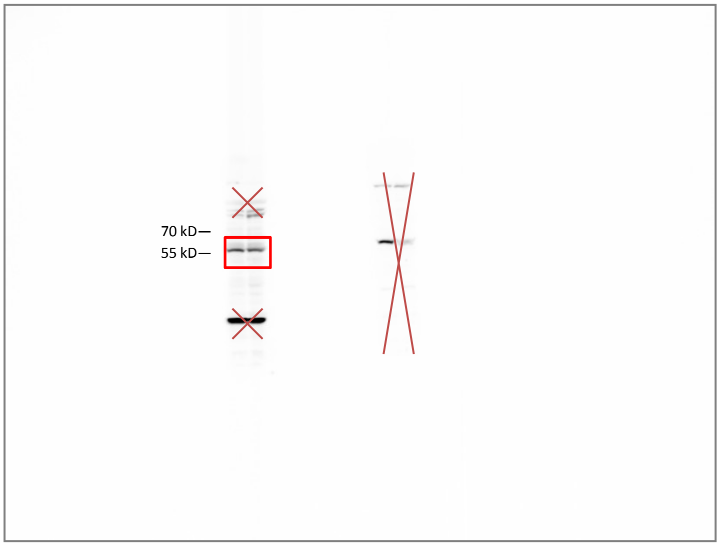

Fig. 5A GAPDH

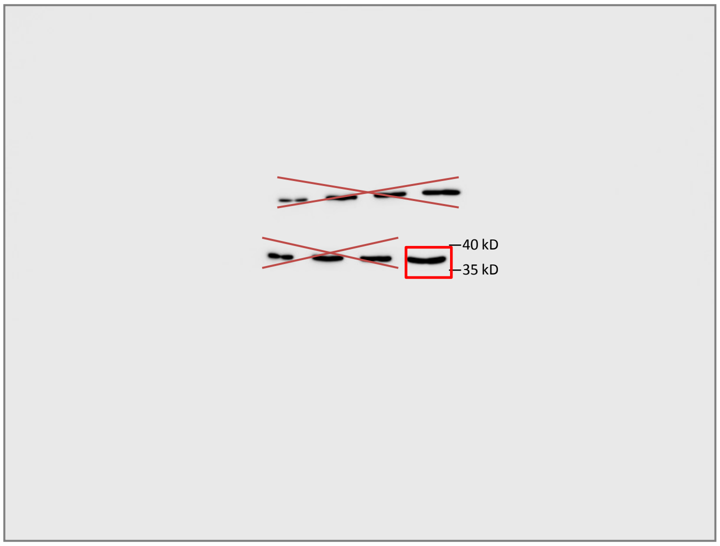

Fig. 5B PTCH1

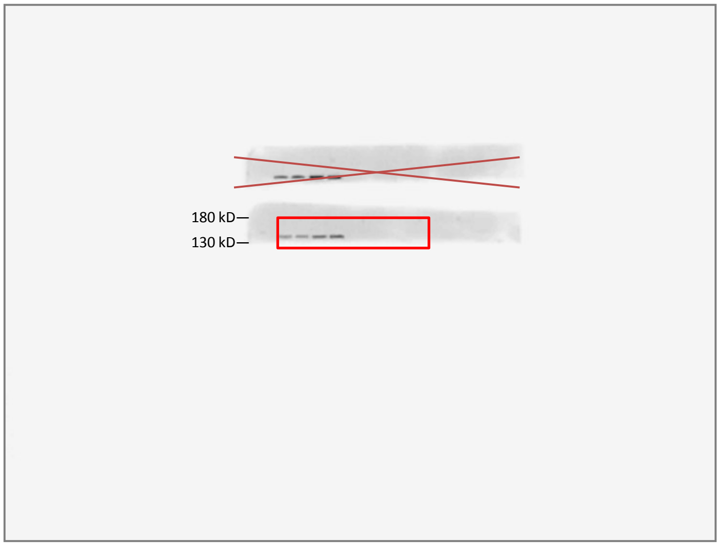

Fig. 5B NRF2

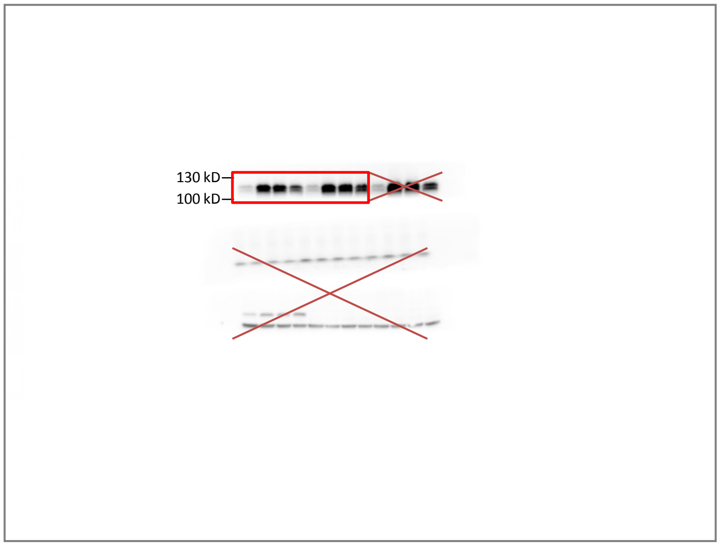

Fig. 5B p62

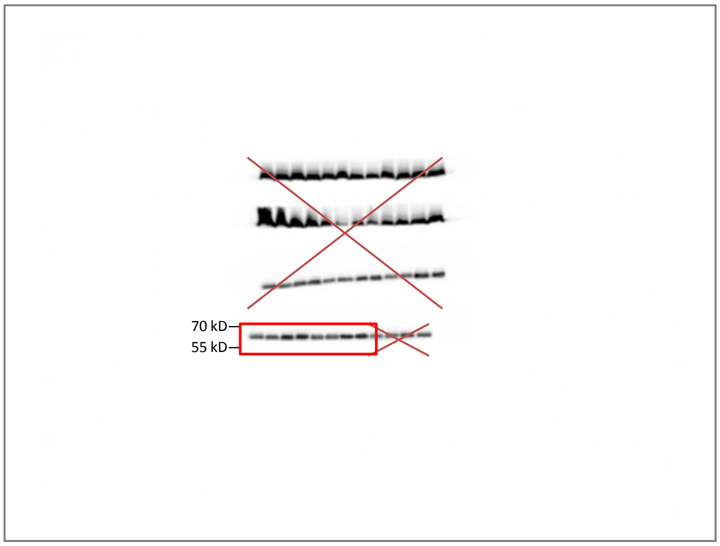

Fig. 5B Ac-Tub

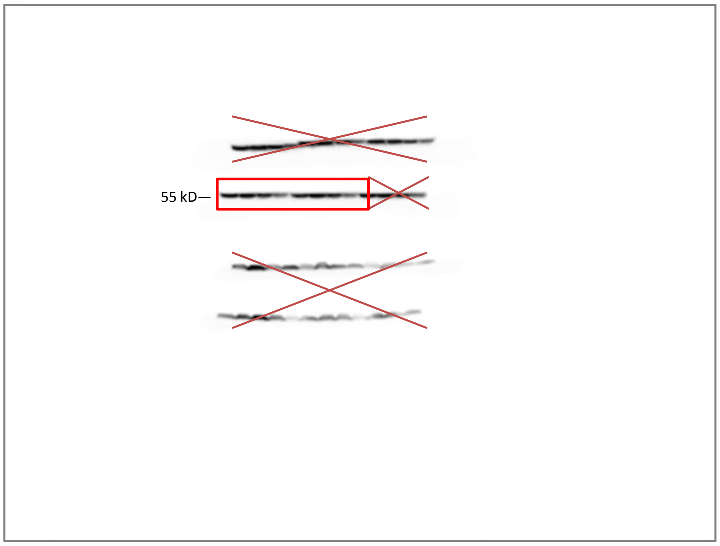

Fig. 5B ARL13B

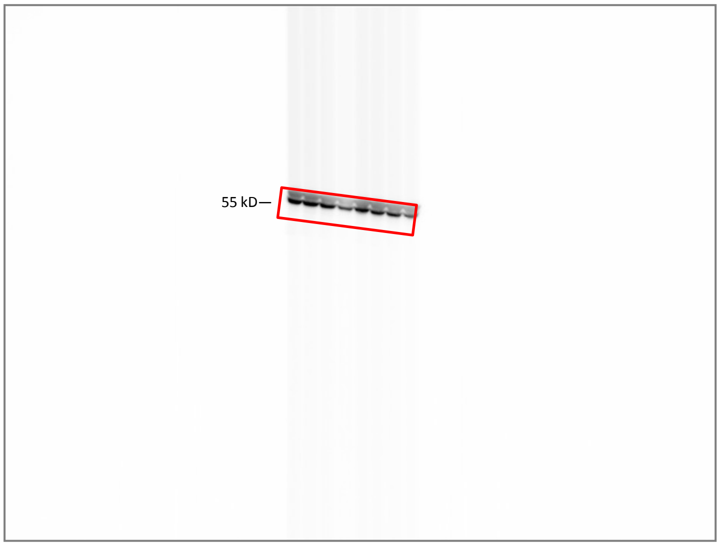

Fig. 5B LC3

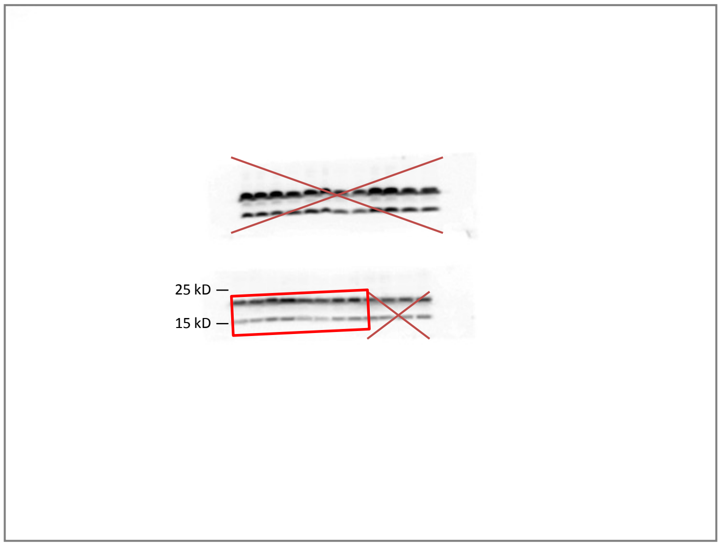

Fig. 5B OFD1

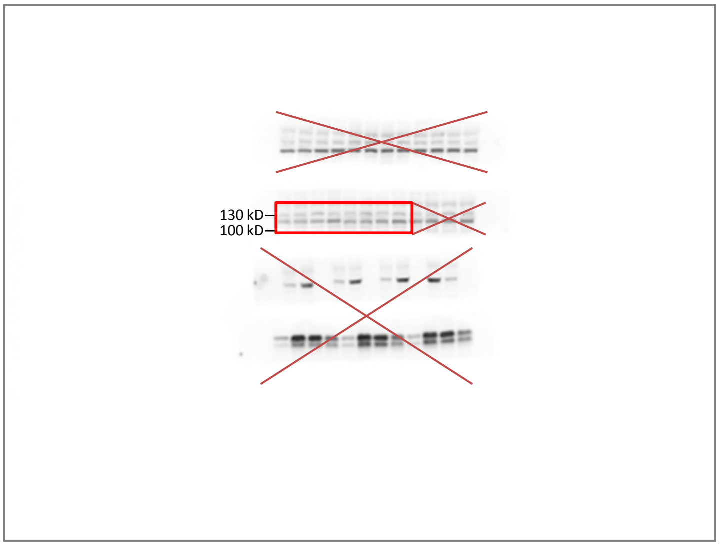

Fig. 5B BBS4

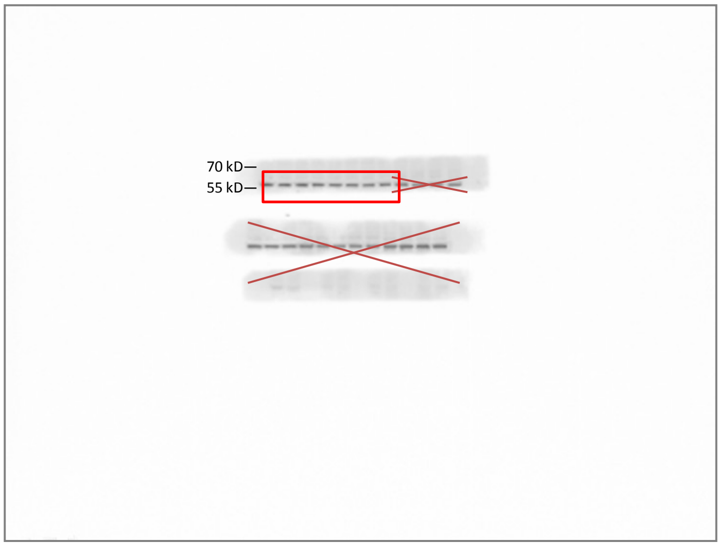

Fig. 5B GAPDH

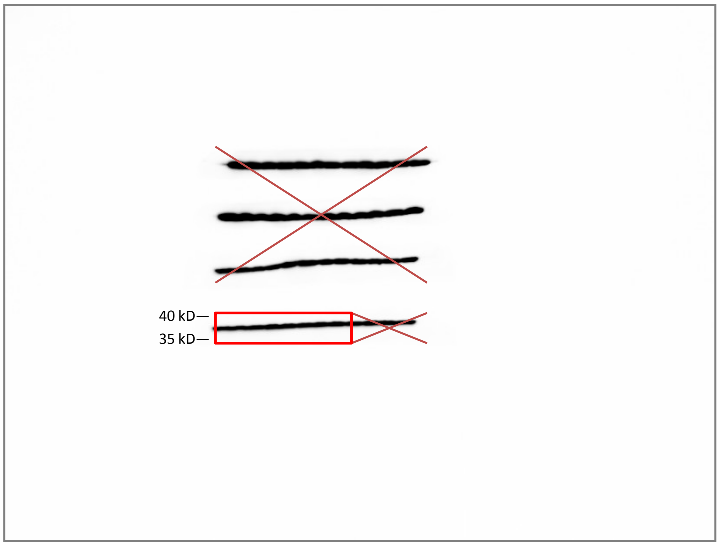

Fig. 5C PTCH1

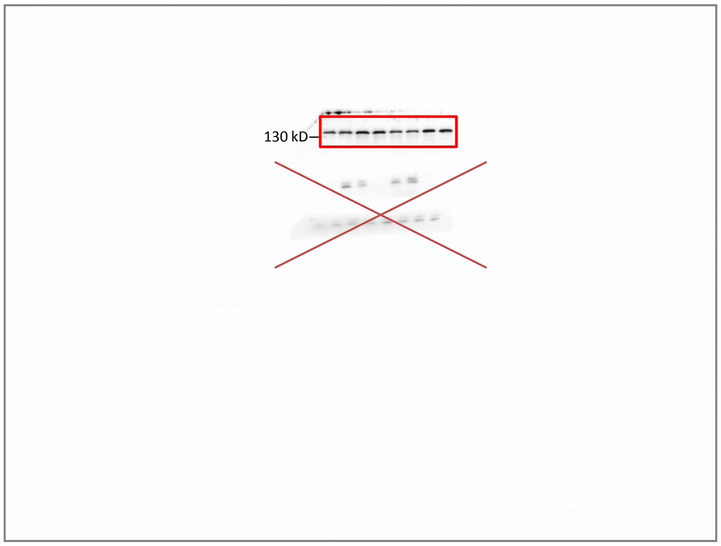

Fig. 5C NRF2

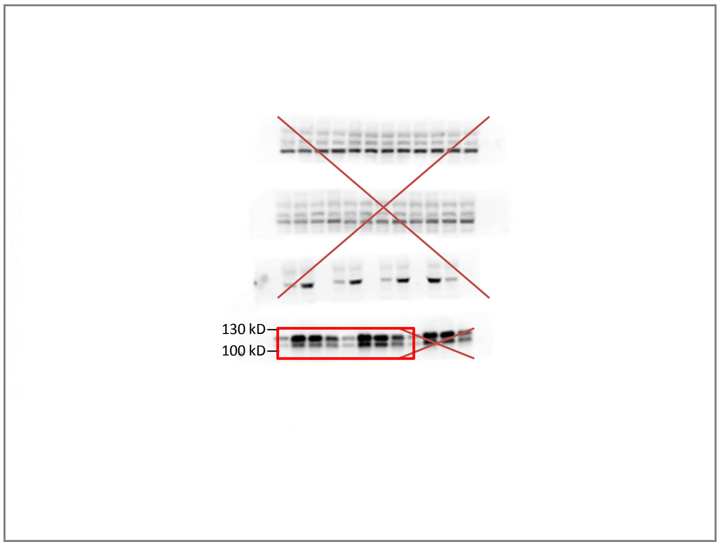

Fig. 5C Ac-Tub

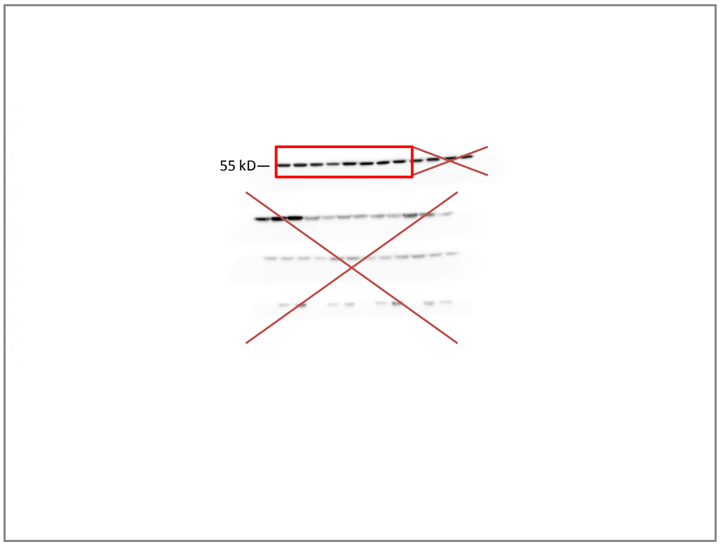

Fig. 5C p62

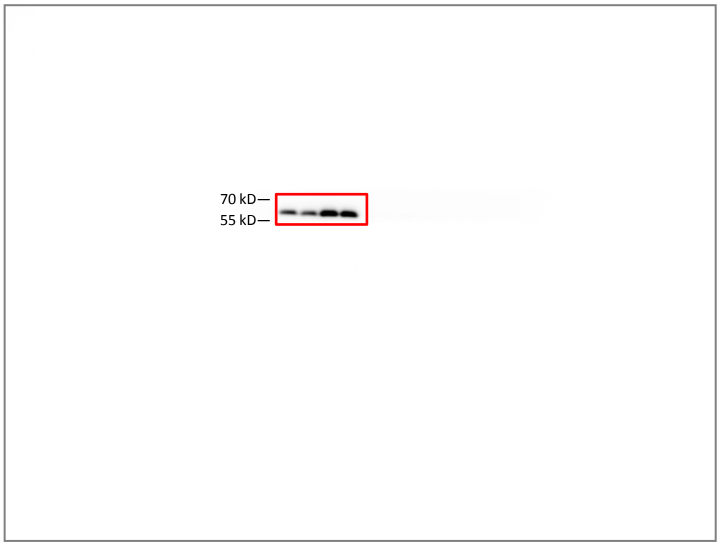

Fig. 5C ARL13B

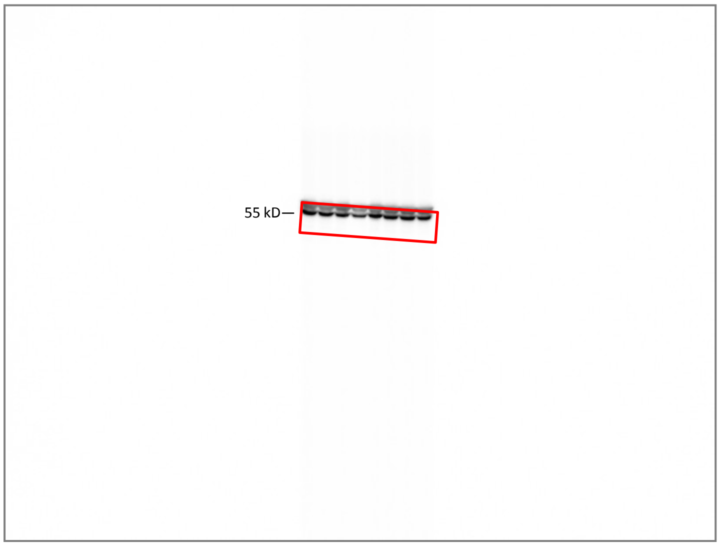

Fig. 5C LC3

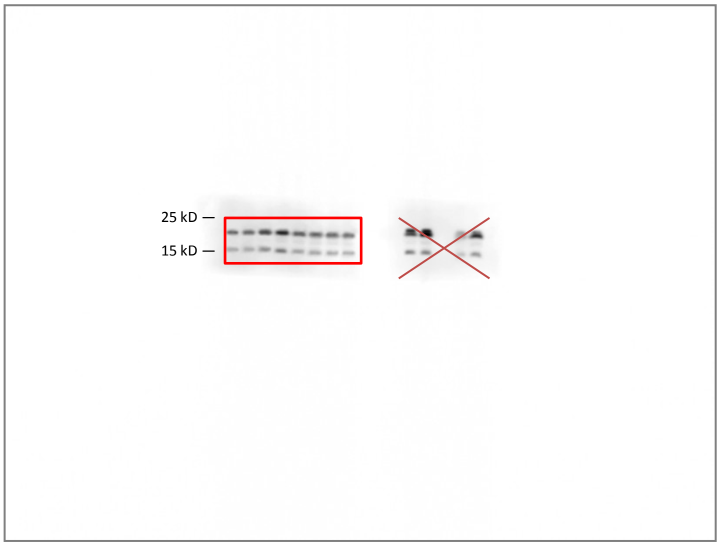

Fig. 5C BBS4

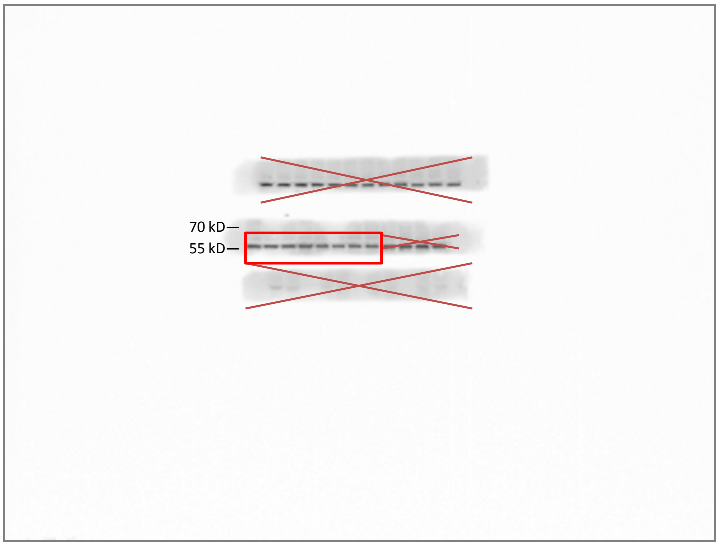

Fig. 5C OFD1

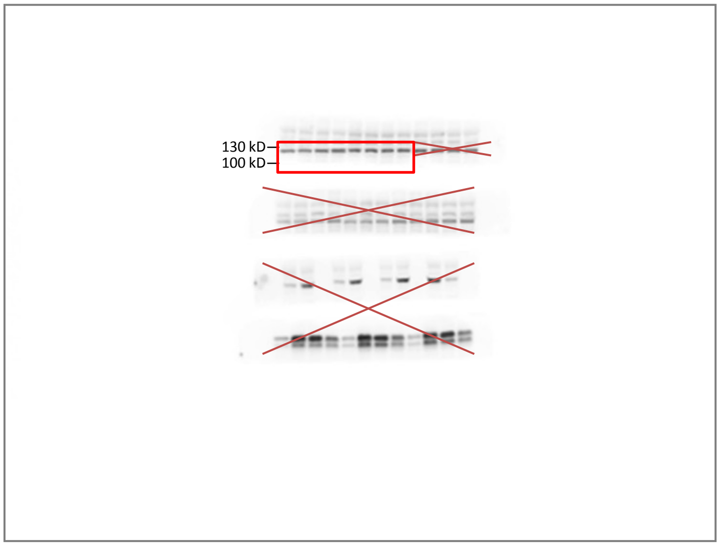

Fig. 5C GAPDH

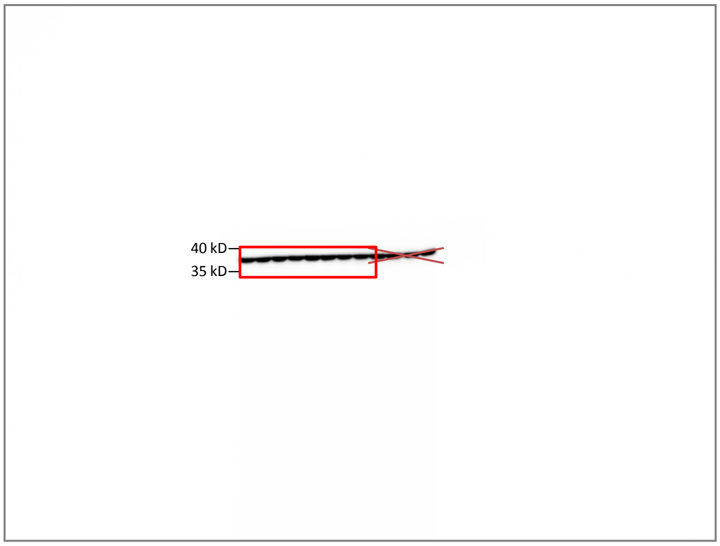

Fig. 5E GLI2

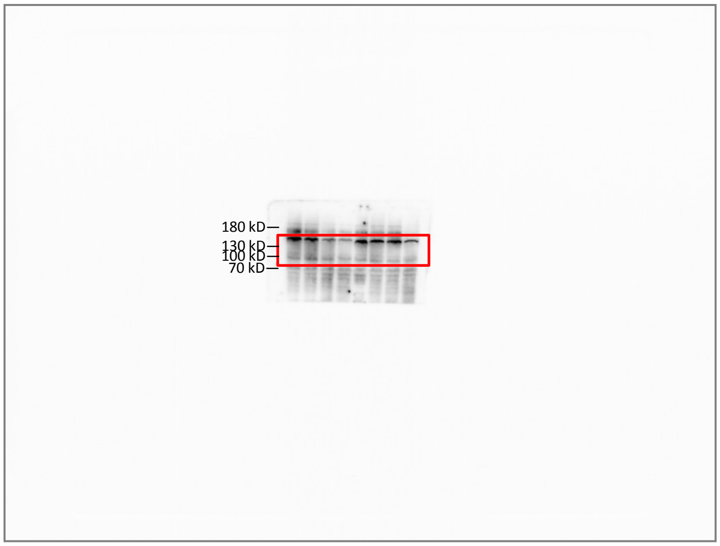

Fig. 5E GLI3

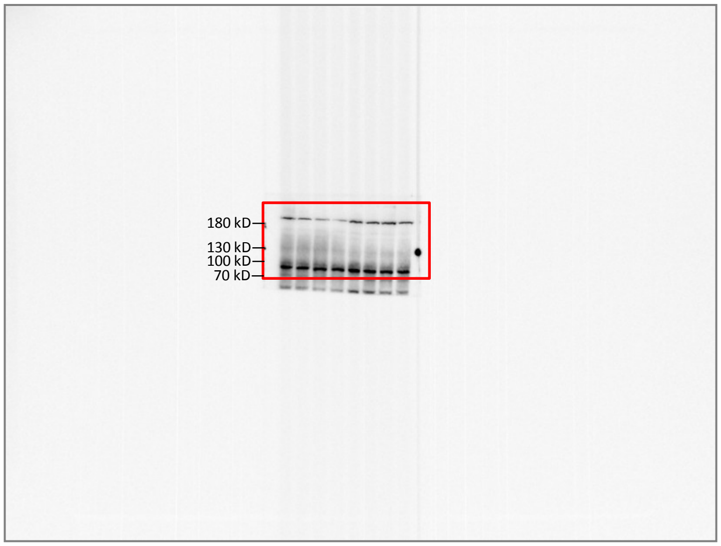

Fig. 5E SMO and GAPDH

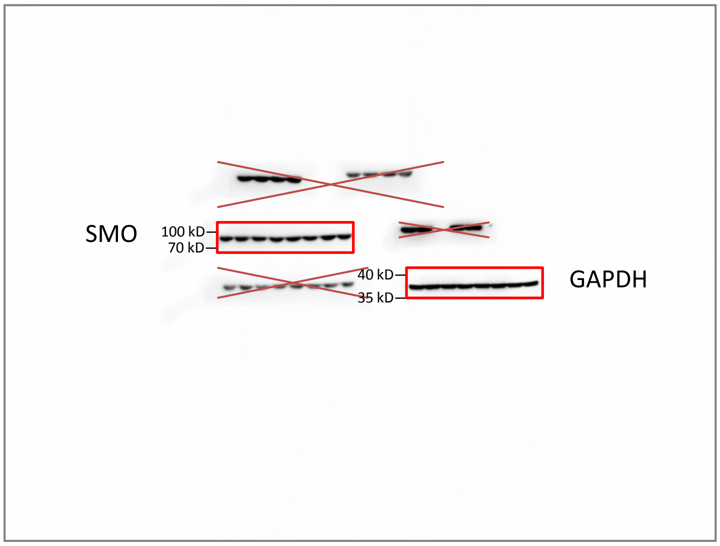

Fig. 6A PTCH1

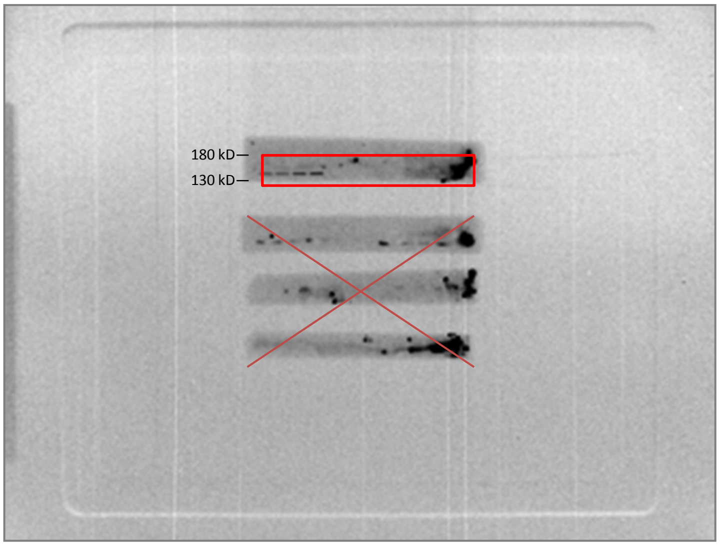

Fig. 6A NRF2

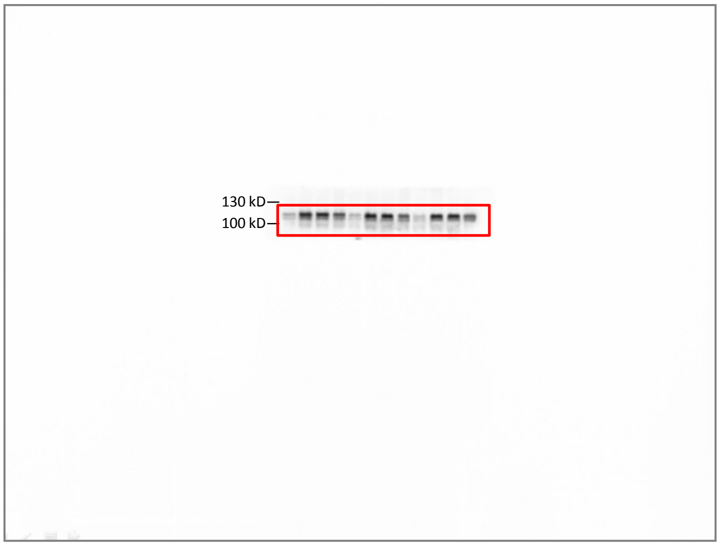

Fig. 6A p62

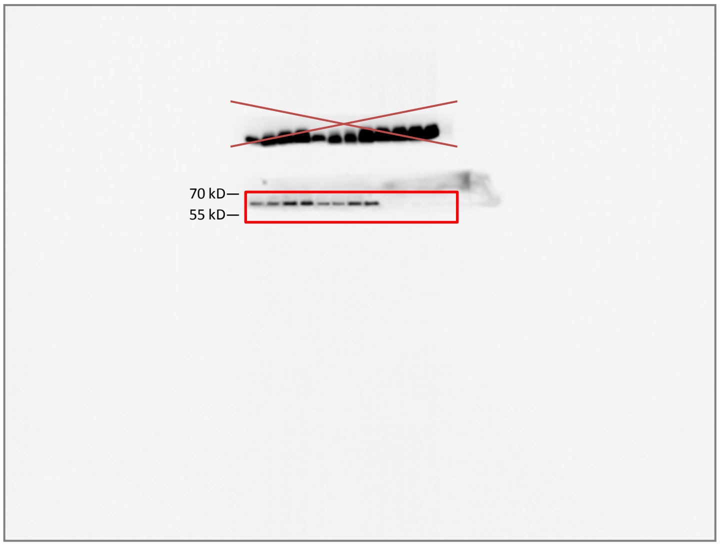

Fig. 6A Ac-Tub

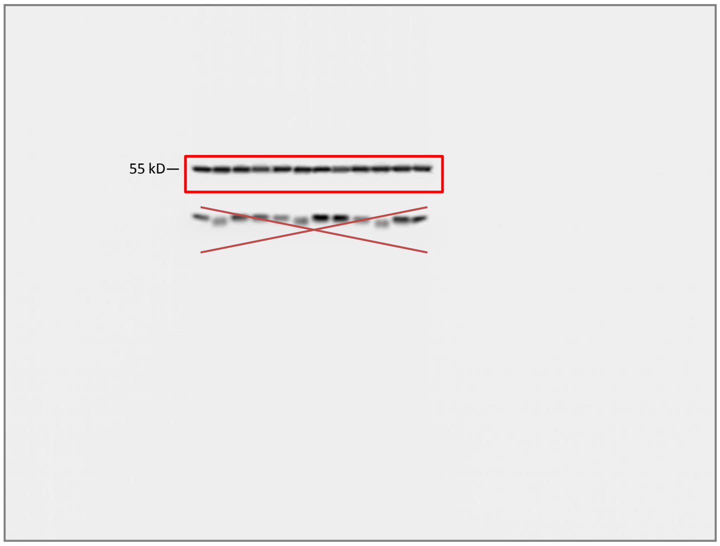

Fig. 6A ARL13B

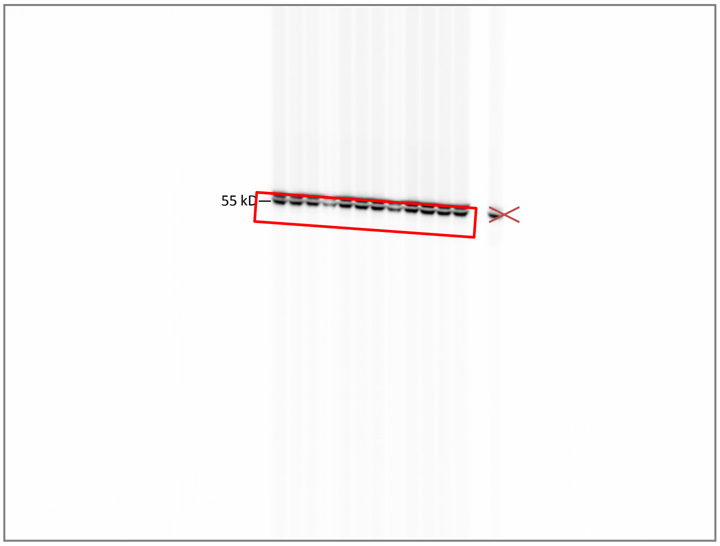

Fig. LC3

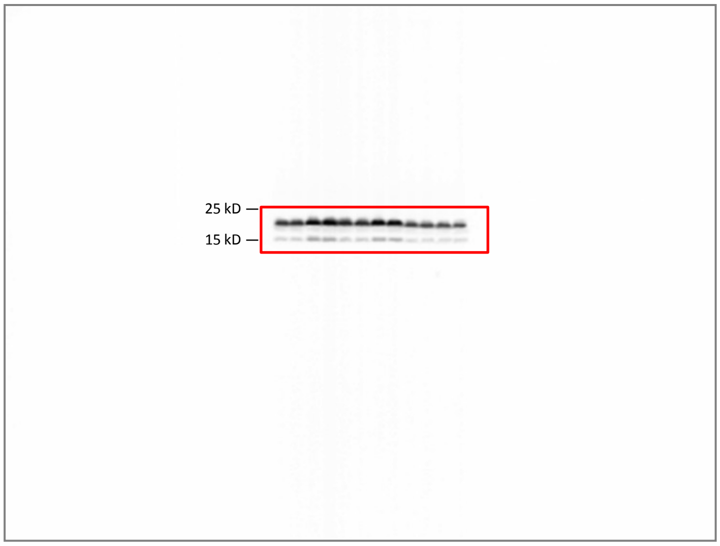

Fig. 6A BBS4

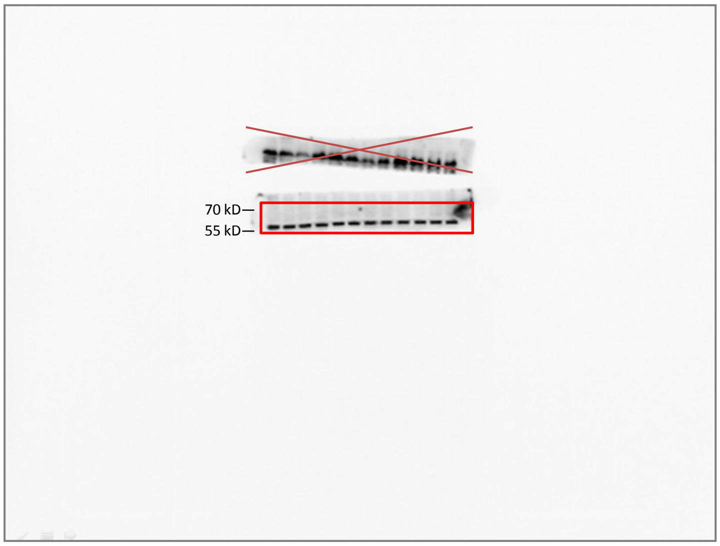

Fig. 6A OFD1

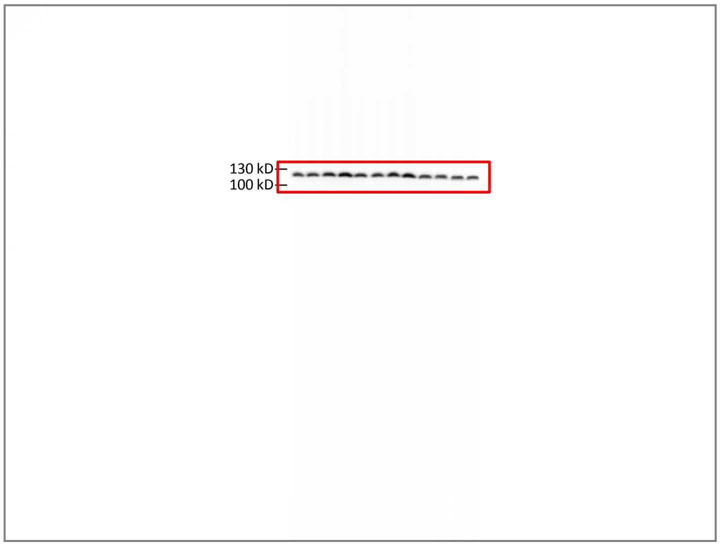

Fig. 6A GAPDH

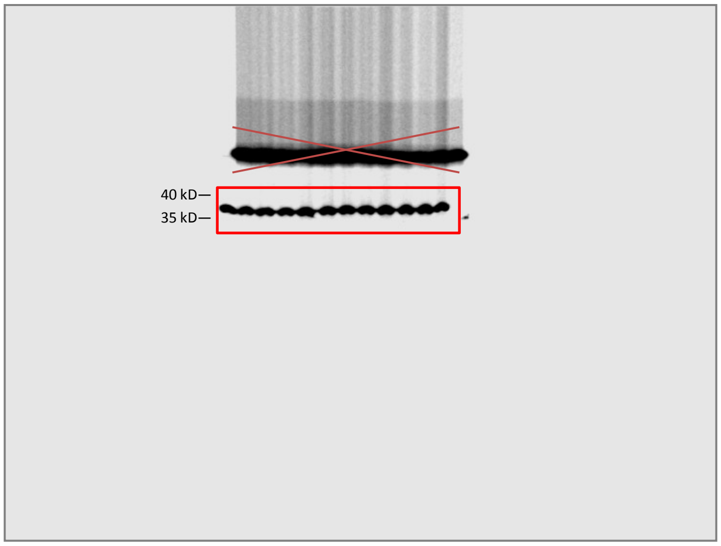

Fig. 6B GLI2

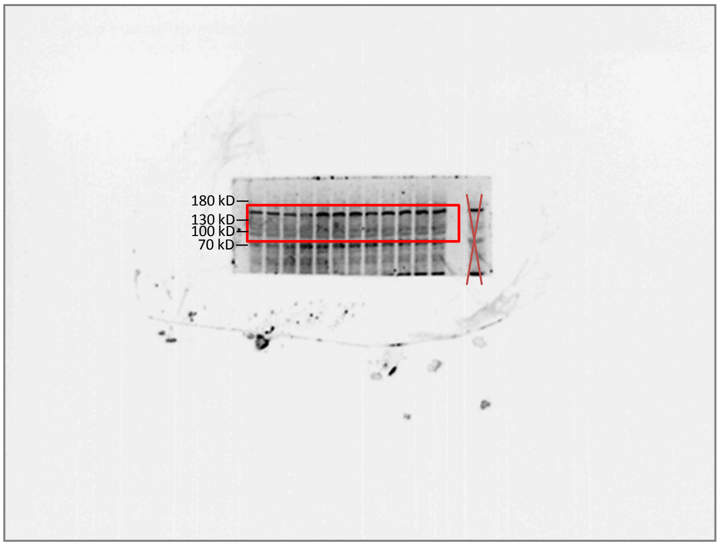

Fig. 6B GLI3

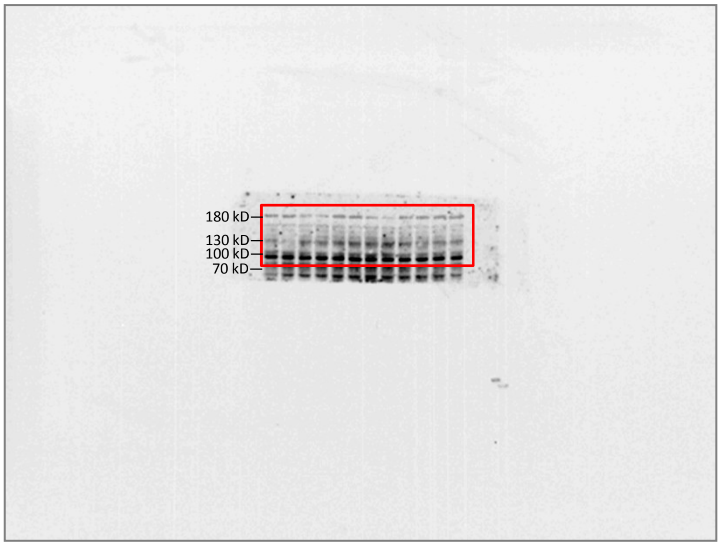

Fig. 6B SMO

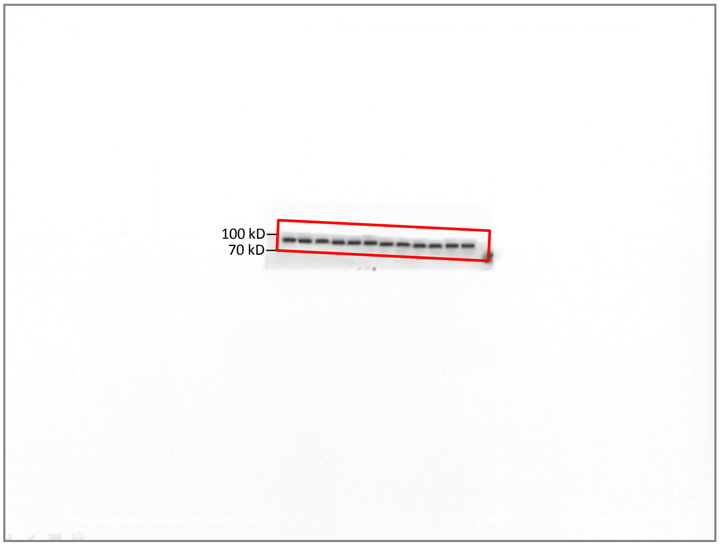

Fig. 6B GAPDH

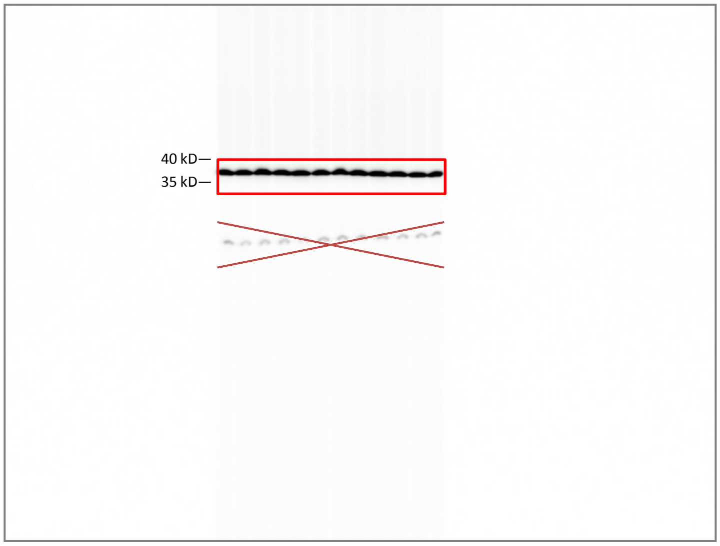

Fig. 7B MEF-NRF2

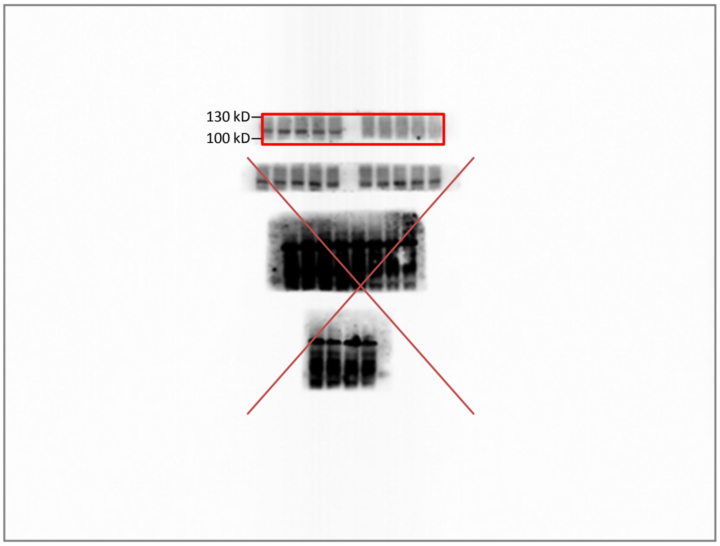

Fig. 7B H838 NRF2

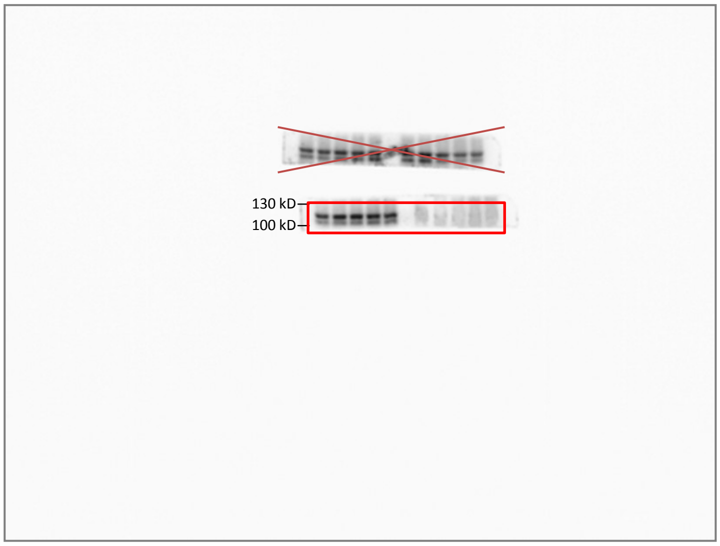

Fig. 7B MEF, H838-Ac-Tub

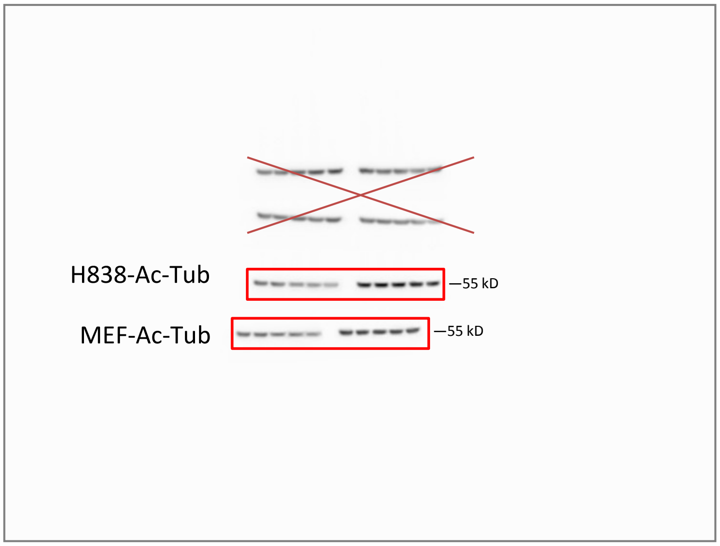

Fig. 7B MEF-ARL13B

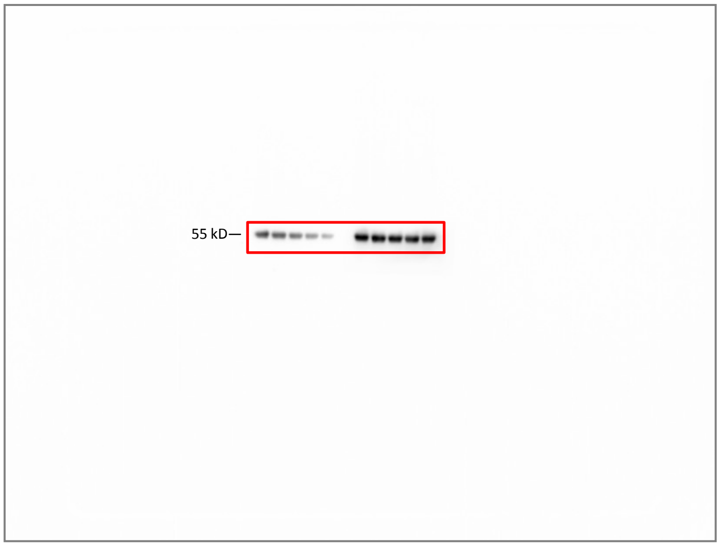

Fig. 7B H838-ARL13B

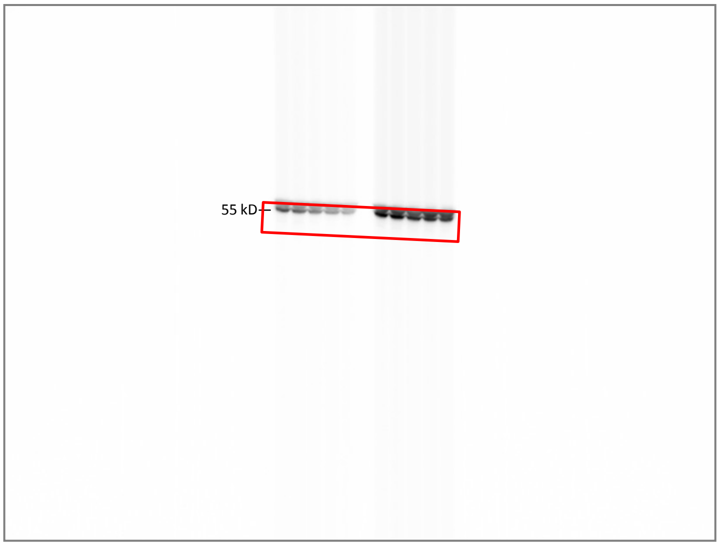

Fig. 7B MEF-GLI2

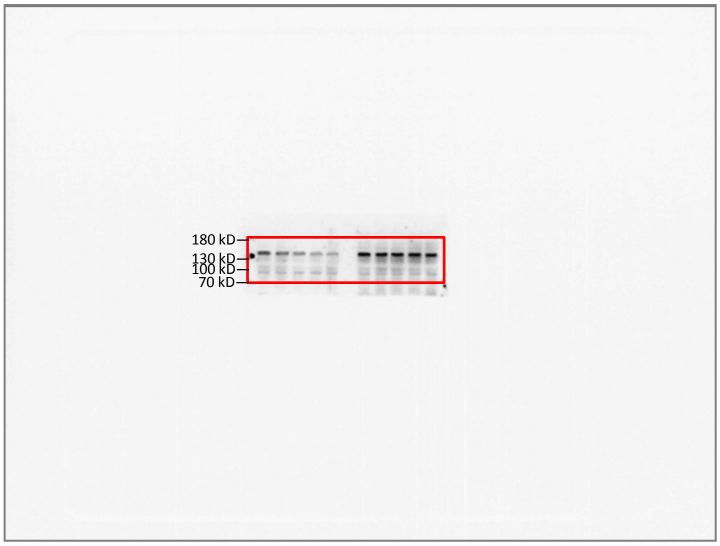

Fig. 7B H838-GLI2

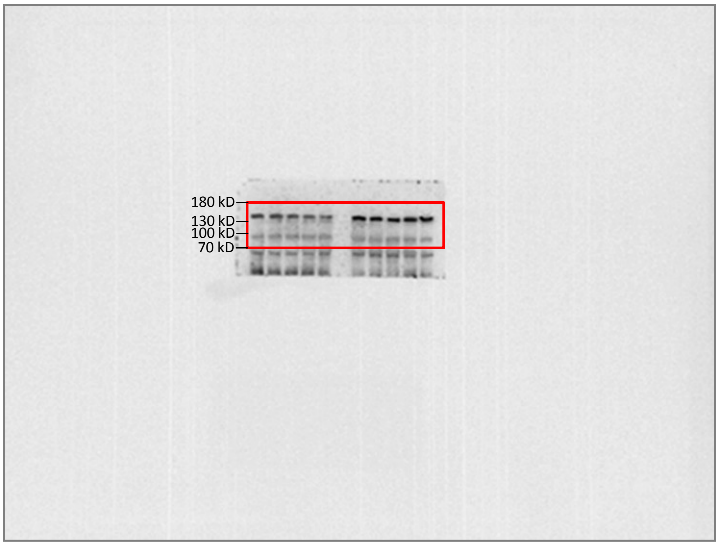

Fig. 7B MEF-GLI3

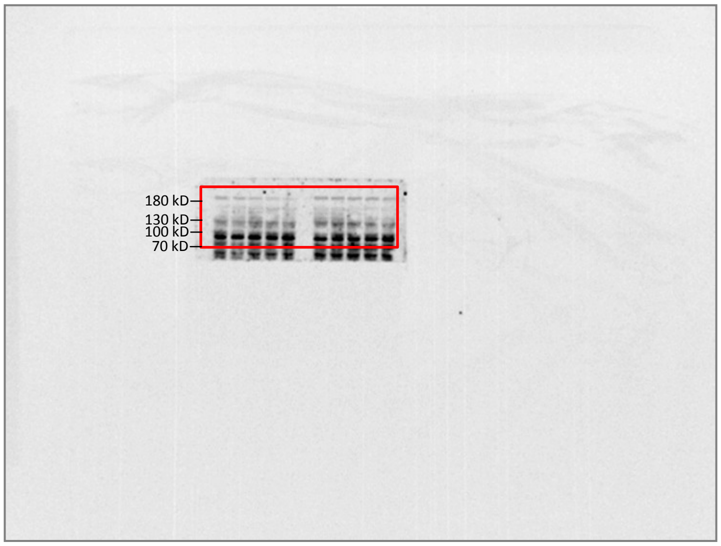

Fig. 7B H838-GLI3

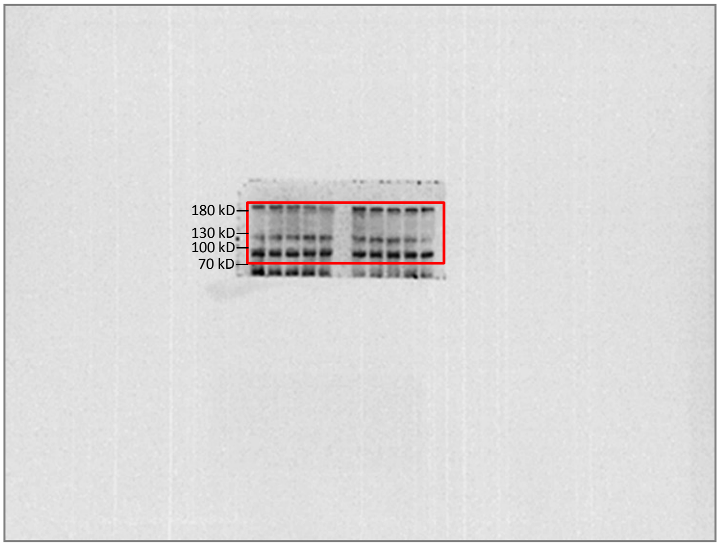

Fig. 7B SMO, GAPDH

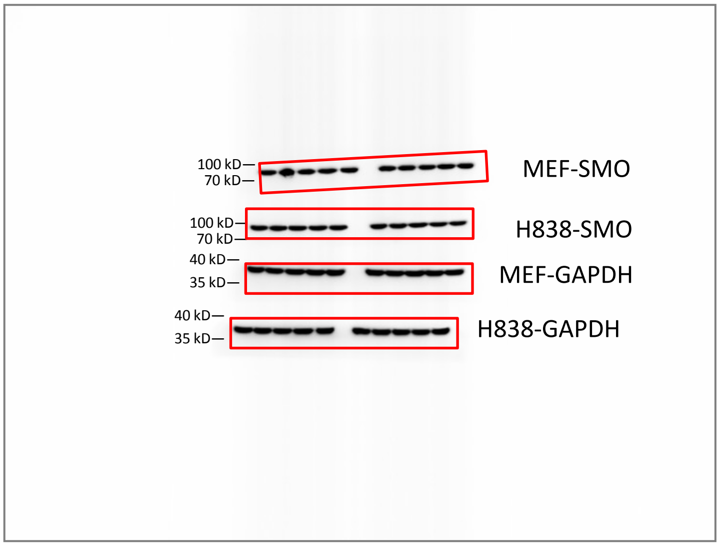

Fig. S5A NRF2

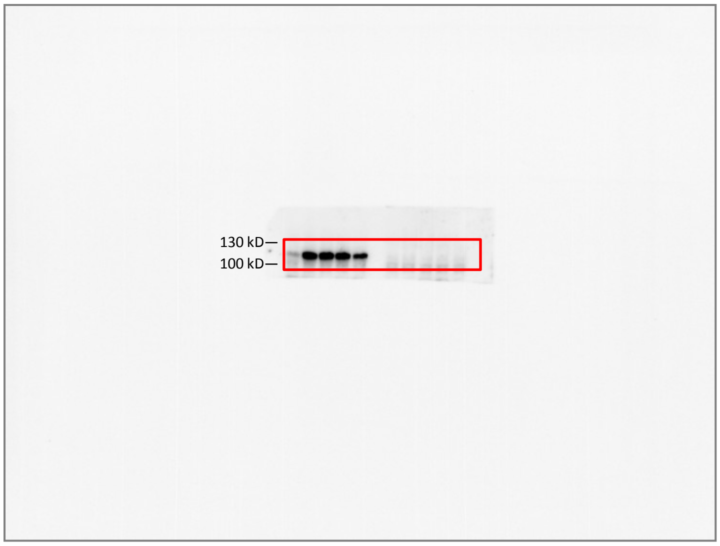

Fig. S5A KEAP1

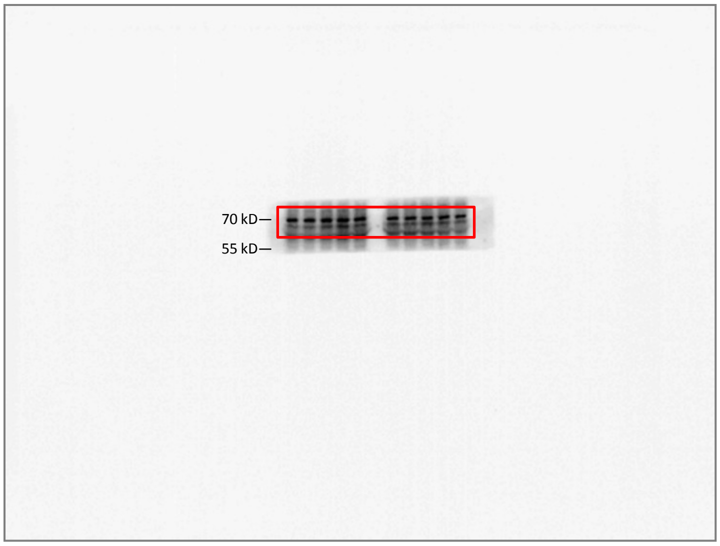

Fig. S5A ARL13B

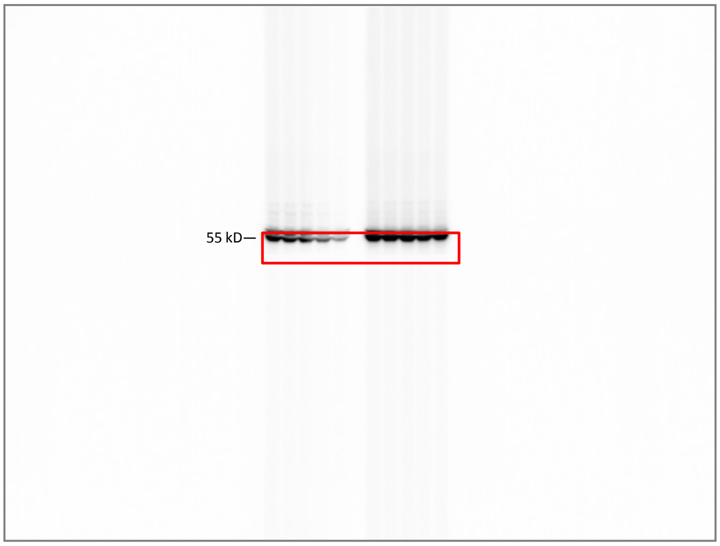

Fig. S5A PTCH1

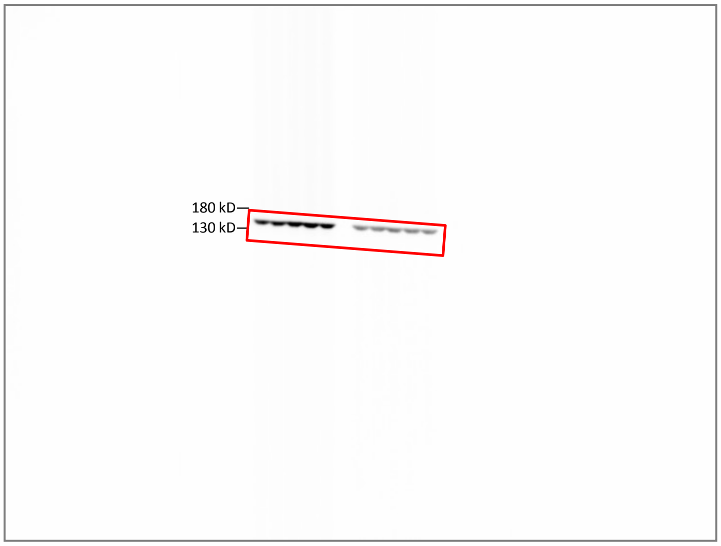

Fig. S5A NQO1

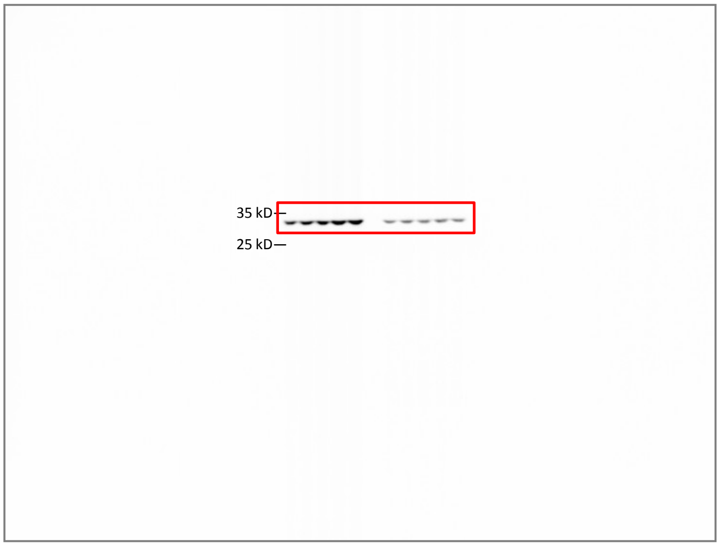

Fig. S5A GAPDH

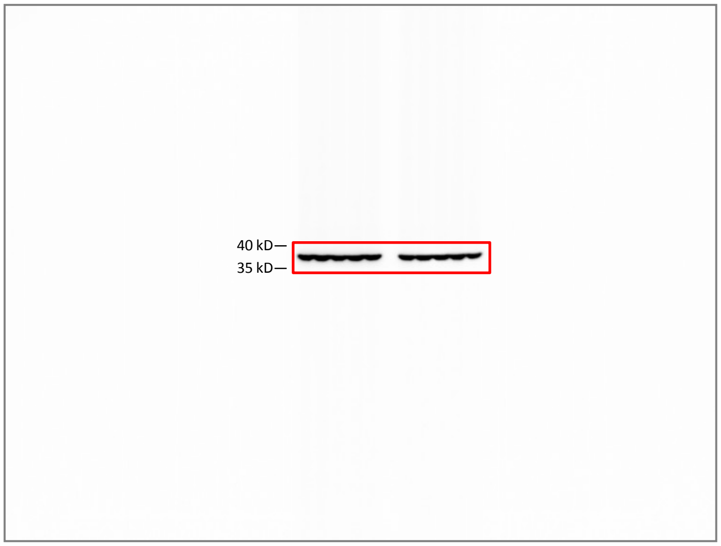

Fig. S5B GLI3

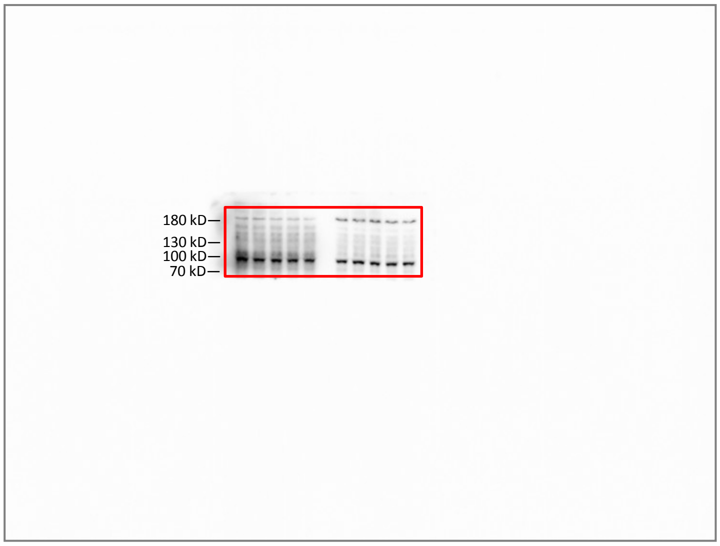

Fig. S5B GLI2

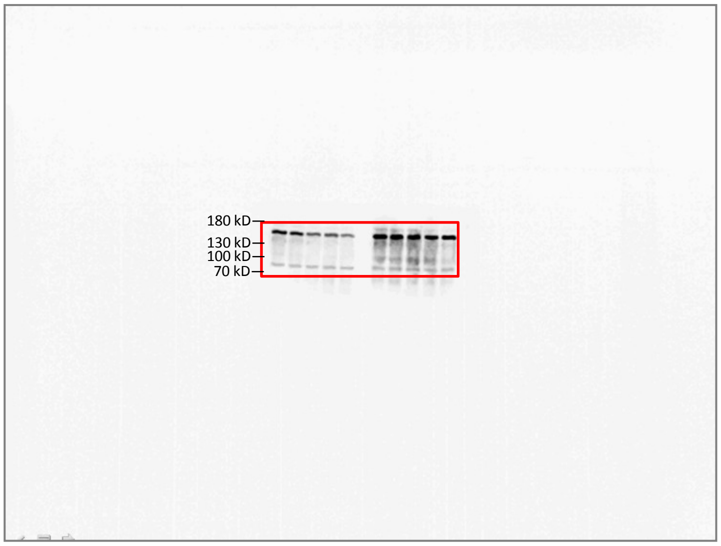

Fig. S5B GAPDH

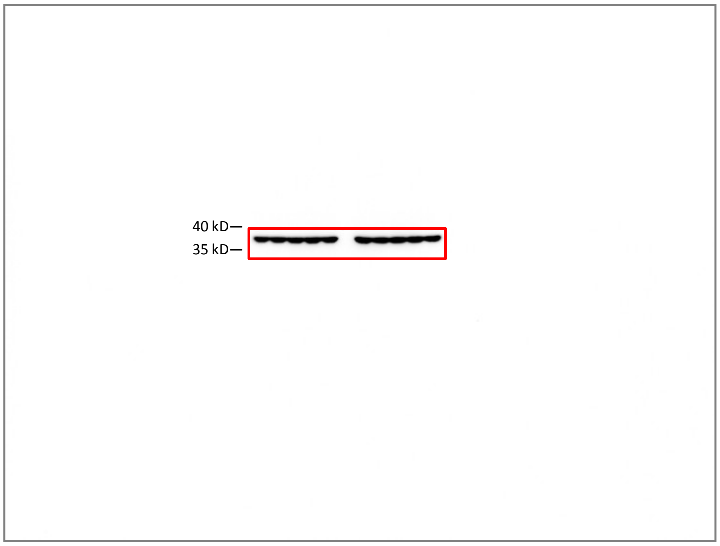

Fig. S5B SMO

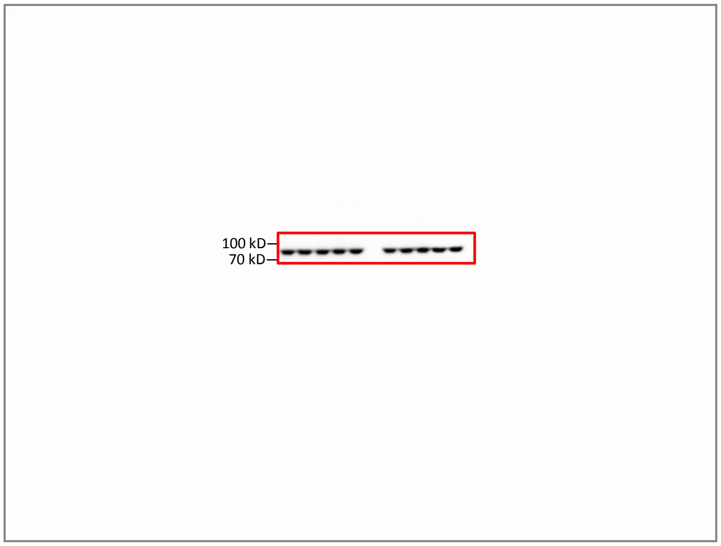

Fig. S8D PTCH1

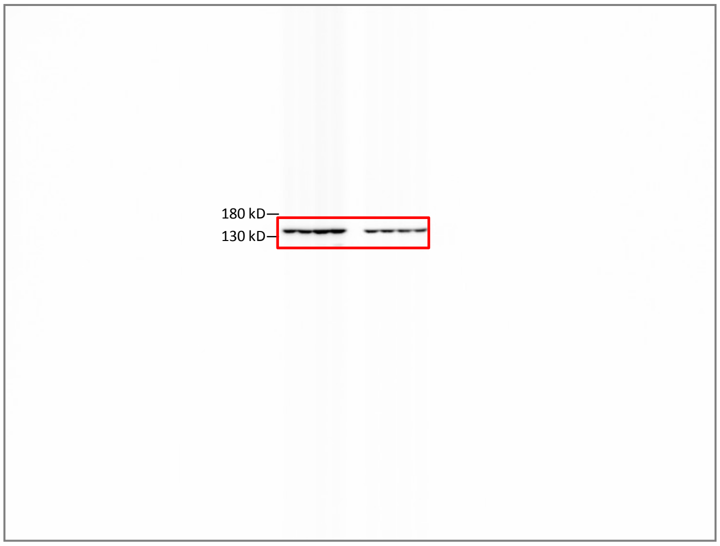

Fig. S8D NRF2

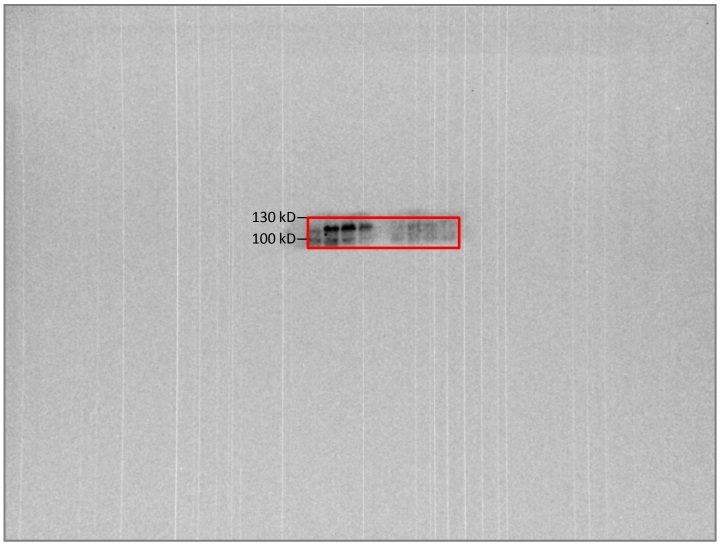

Fig. S8D p62

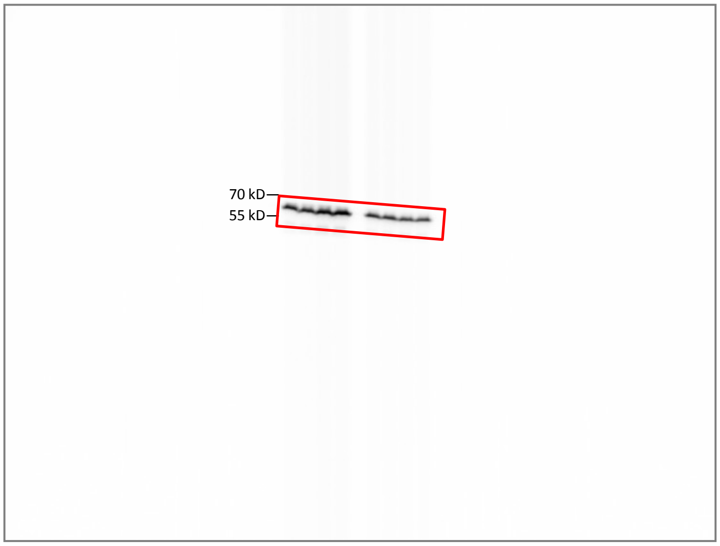

Fig. S8D Ac-Tub

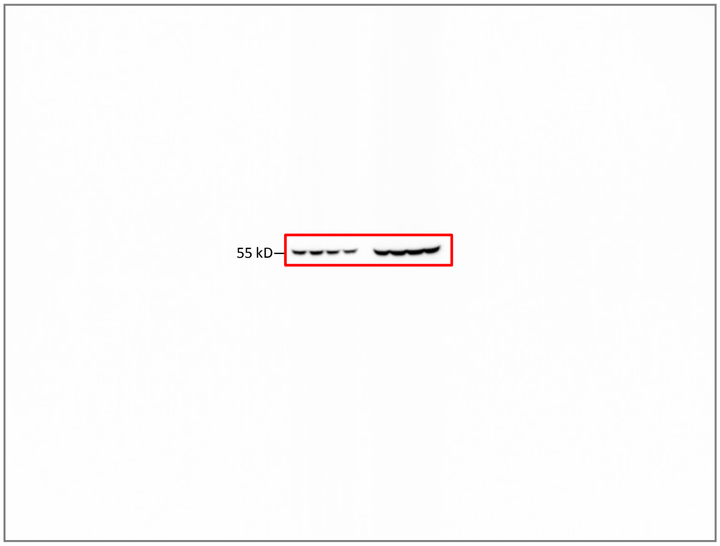

Fig. S8D LC3

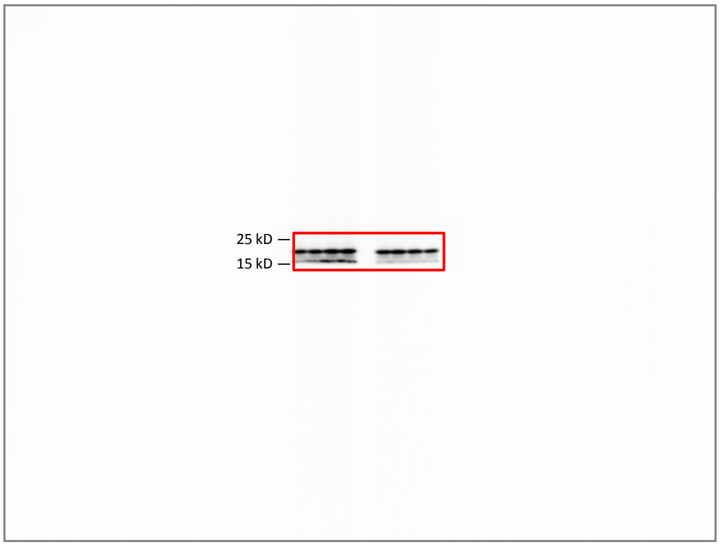

Fig. S8D OFD1

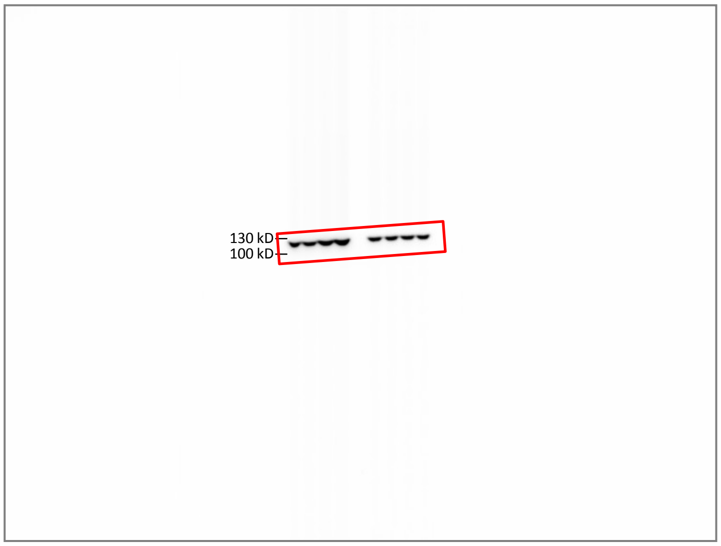

Fig. S8D BBS4

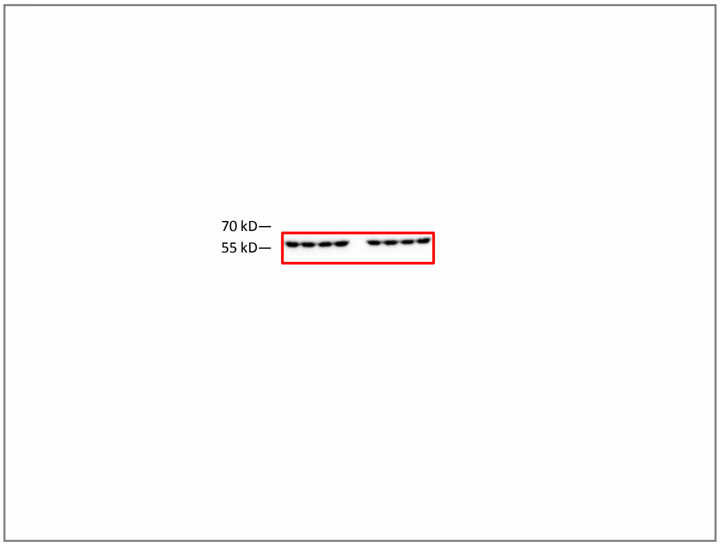

Fig. S8D GAPDH

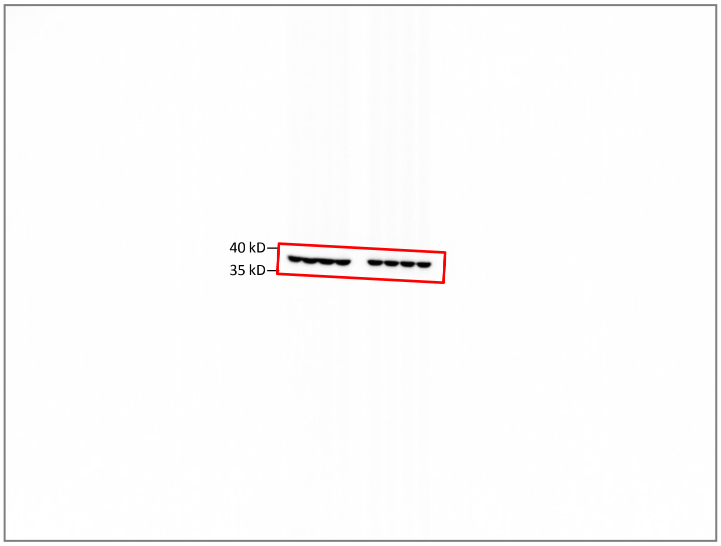

Fig. S11A NRF2

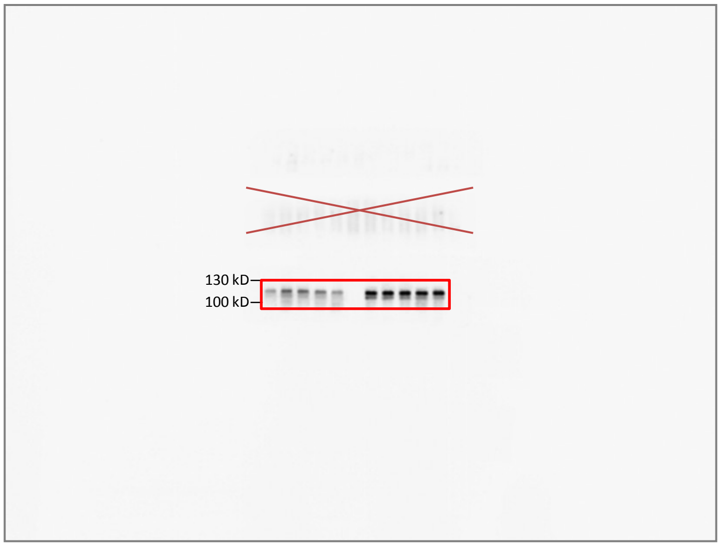

Fig. S11A KEAP1

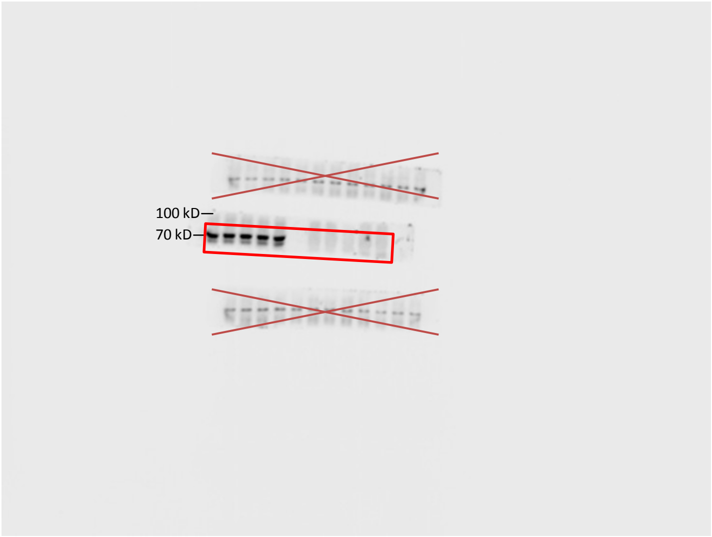

Fig. S11A Ac-Tub

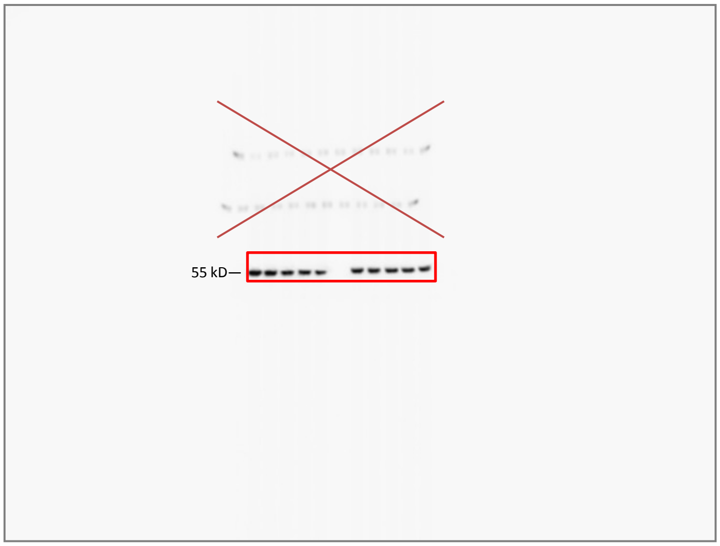

Fig. S11A NQO1

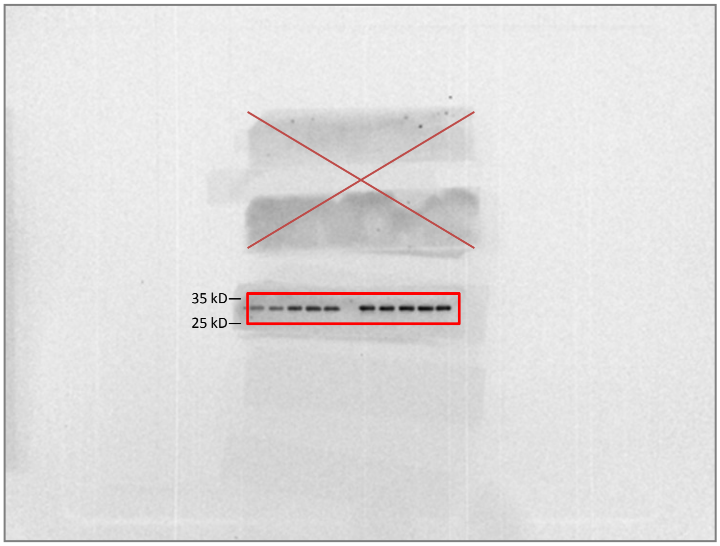

Fig. S11A GAPDH

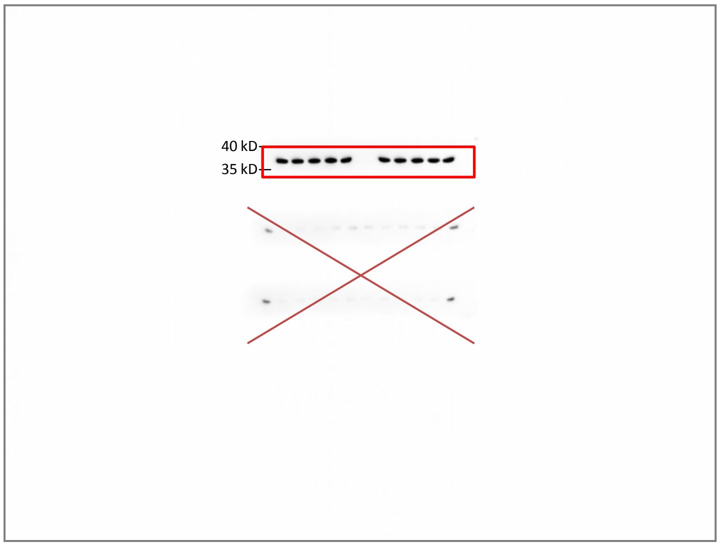

Fig. S11B NRF2

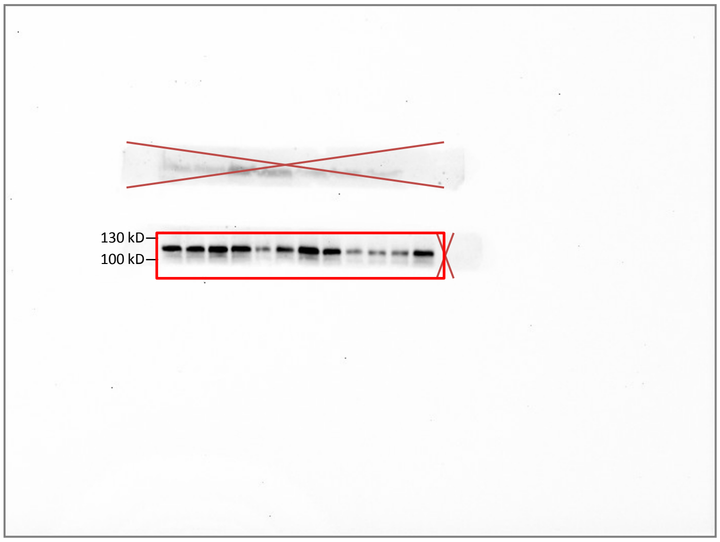

Fig. S11B KEAP1

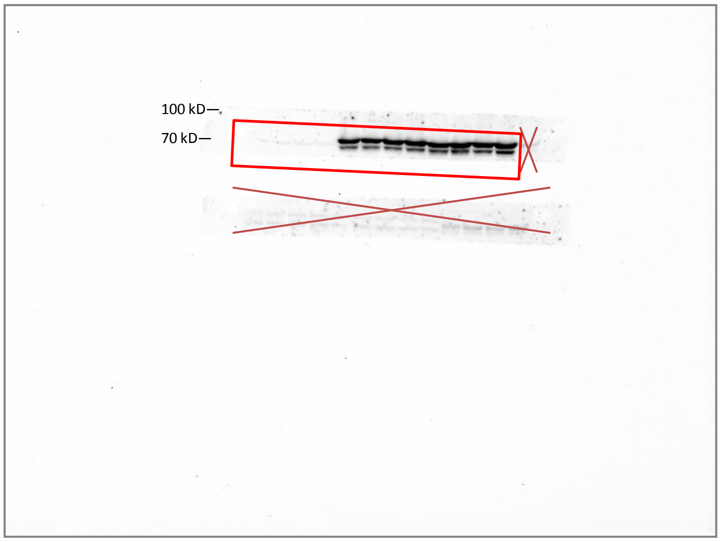

Fig. S11B NQO1

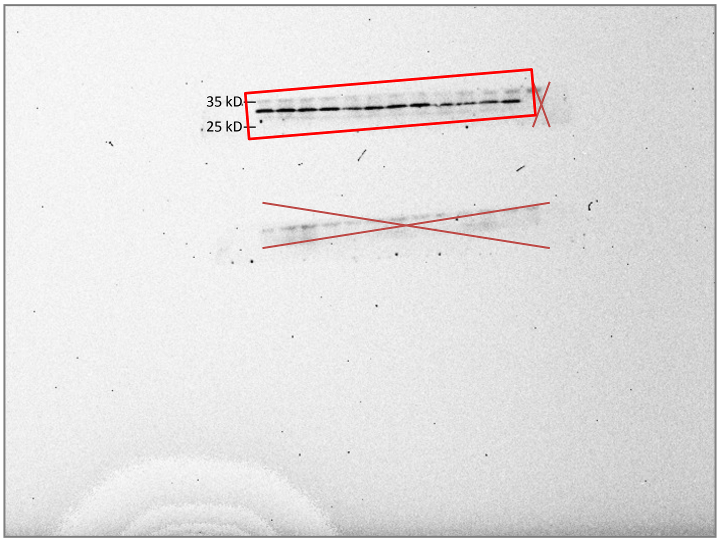

Fig. S11B GAPDH

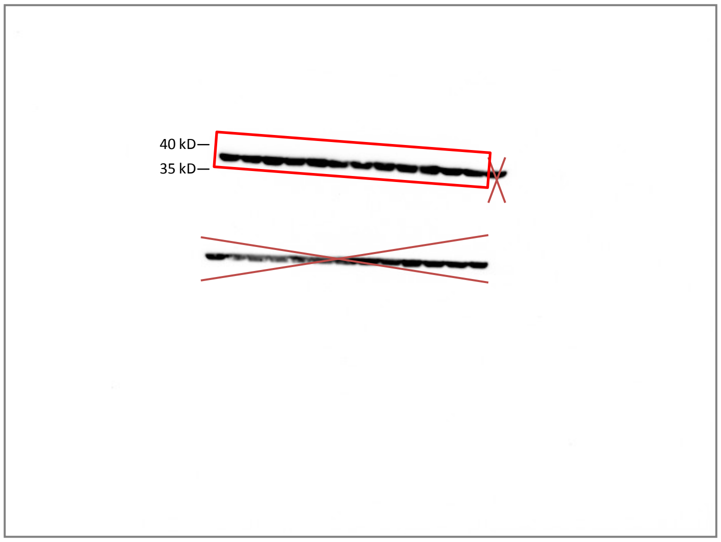

Supplement: S1 Raw Images — (PDF) [file pbio.3000620.s013.pdf]
